# New vectors in northern Sarawak, Malaysian Borneo, for the zoonotic malaria parasite, *Plasmodium knowlesi*

**DOI:** 10.1186/s13071-020-04345-2

**Published:** 2020-09-15

**Authors:** Joshua X. D. Ang, Khamisah A. Kadir, Dayang S. A. Mohamad, Asmad Matusop, Paul C. S. Divis, Khatijah Yaman, Balbir Singh

**Affiliations:** 1 Malaria Research Centre, Faculty of Medicine & Health Sciences, Universiti Malaysia Sarawak, Kuching, Sarawak Malaysia; 2 Sarawak Department of Health, Kuching, Sarawak Malaysia

**Keywords:** Zoonosis, Malaria, *Plasmodium knowlesi*, Vector, *Anopheles balabacensis*, *Anopheles donaldi*

## Abstract

**Background:**

*Plasmodium knowlesi* is a significant cause of human malaria in Sarawak, Malaysian Borneo. Only one study has been previously undertaken in Sarawak to identify vectors of *P. knowlesi*, where *Anopheles latens* was incriminated as the vector in Kapit, central Sarawak. A study was therefore undertaken to identify malaria vectors in a different location in Sarawak.

**Methods:**

Mosquitoes found landing on humans and resting on leaves over a 5-day period at two sites in the Lawas District of northern Sarawak were collected and identified. DNA samples extracted from salivary glands of *Anopheles* mosquitoes were subjected to nested PCR malaria-detection assays. The small subunit ribosomal RNA (*SSU* rRNA) gene of *Plasmodium* was sequenced, and the internal transcribed spacer 2 (ITS2) and mitochondrial cytochrome *c* oxidase subunit 1 (*cox*1) gene of the mosquitoes were sequenced from the *Plasmodium*-positive samples for phylogenetic analysis.

**Results:**

Totals of 65 anophelines and 127 culicines were collected. By PCR, 6 *An. balabacensis* and 5 *An. donaldi* were found to have single *P. knowlesi* infections while 3 other *An. balabacensis* had either single, double or triple infections with *P. inui*, *P. fieldi*, *P. cynomolgi* and *P. knowlesi*. Phylogenetic analysis of the *Plasmodium SSU* rRNA gene confirmed 3 *An. donaldi* and 3 *An. balabacensis* with single *P. knowlesi* infections, while 3 other *An. balabacensis* had two or more *Plasmodium* species of *P. inui*, *P. knowlesi*, *P. cynomolgi* and some species of *Plasmodium* that could not be conclusively identified. Phylogenies inferred from the ITS2 and/or *cox*1 sequences of *An. balabacensis* and *An. donaldi* indicate that they are genetically indistinguishable from *An. balabacensis* and *An. donaldi*, respectively, found in Sabah, Malaysian Borneo.

**Conclusions:**

Previously *An. latens* was identified as the vector for *P. knowlesi* in Kapit, central Sarawak, Malaysian Borneo, and now *An. balabacensis* and *An. donaldi* have been incriminated as vectors for zoonotic malaria in Lawas, northern Sarawak. 
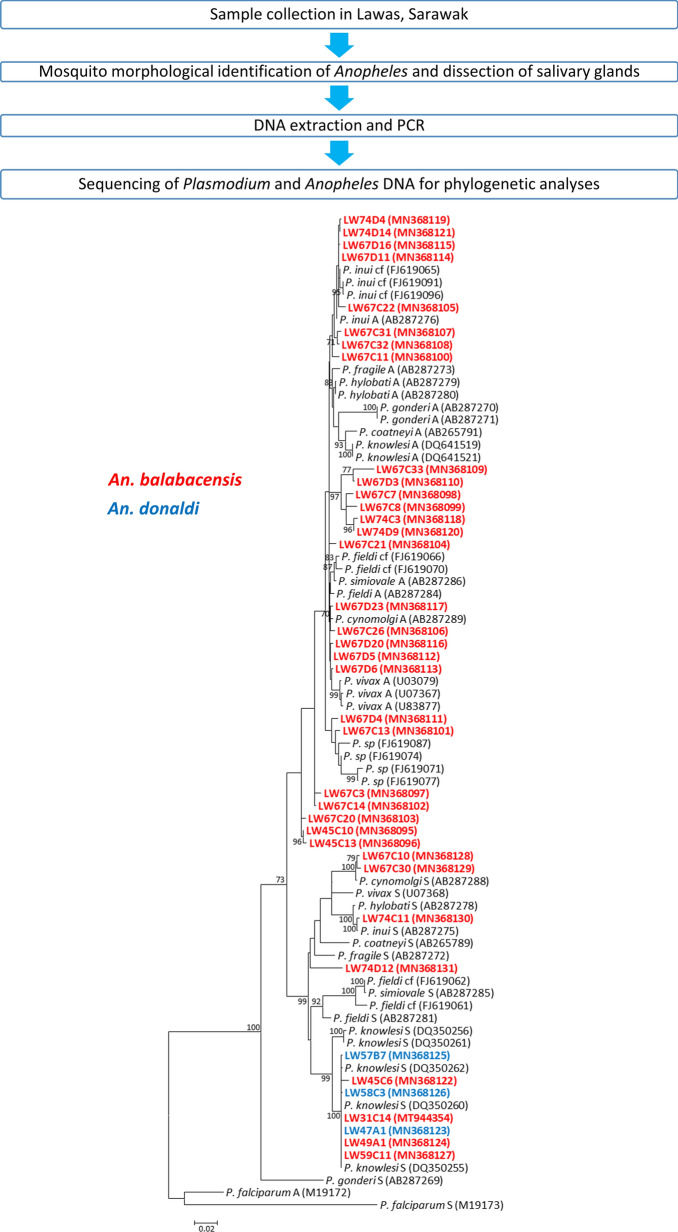

## Background

Human infections with *Plasmodium knowlesi*, a malaria parasite of long-tailed and pig-tailed macaques [1], were thought to be extremely rare until a large number of human cases were described in the Kapit Division of Sarawak State, Malaysian Borneo [2]. Knowlesi malaria cases have been reported from different locations in Malaysia [2–7], in almost all countries in Southeast Asia [8–17] except Timor Leste, and also in the Nicobar and Andaman Islands of India [18]. This simian malaria parasite is now the most common cause for malaria admissions to hospitals in Malaysia, where in the years 2017–2019, 10,968 cases were reported, with 87% occurring in the states of Sabah and Sarawak in Malaysian Borneo (unpublished data, Ministry of Health Malaysia) [19]. Despite now being recognised as a significant cause of malaria in humans and an additional challenge to malaria elimination in Southeast Asia [20–23], there remains a paucity of information regarding the vectors of *P. knowlesi* in nature.

In the period between 1951 and 1997, malaria vectors in Sarawak, Malaysian Borneo were determined by examining dissected salivary glands for the presence of sporozoites. Information about vectors of human malaria parasites was first reported by McArthur [24] in his 1951 review regarding the importance of the *Leucosphyrus* Group of *Anopheles* in malaria transmission. Dissection of various species of *Anopheles* mosquitoes was carried out in the Kuching General Hospital and oocysts were found in the midguts of *An. latens*. Later, in 1956 and 1995, *An. latens*, *An. barbirostris* (*s.l*.) and *An. donaldi* were incriminated as the vectors for human malaria parasites in Upper Baram [25, 26]. In Miri, Chang et al. [27] discovered malaria sporozoites in the salivary glands of *An. donaldi* and *An. letifer* in an oil palm plantation in Miri in 1997. However, the sporozoites of *Plasmodium* spp. harboured by these vectors could not be determined accurately since molecular tools were not available when these studies were undertaken. Despite this, intravenous inoculation of sporozoites isolated from the simiophilic *An. hackeri* in 1961 into an uninfected rhesus monkey demonstrated that *An. hackeri* was the natural vector of *P. knowlesi* in Peninsular Malaysia [28]. The parasite was morphologically identified from the blood of the rhesus monkey inoculated with the sporozoites. It was only in 1999, in Belaga, Sarawak that an ELISA was used for detection of sporozoites of *Plasmodium* spp. in mosquitoes, but the ELISA could only detect two species of *Plasmodium*, *P. falciparum* and *P. vivax* [29].

The first and only entomological study in Sarawak, Malaysian Borneo that identified *Plasmodium* species using molecular methods was conducted in 2005–2006. Using nested PCR assays on DNA extracted from salivary glands of mosquitoes in the Kapit District, *An. latens* was incriminated as the vector of *P. knowlesi* in this region [30]. Subsequently, similar studies utilising molecular tools have incriminated *An. cracens* as the vector of *P. knowlesi* in Kuala Lipis, Peninsular Malaysia [31] while *An. balabacensis* was found to harbour sporozoites of *P. coatneyi*, *P. cynomolgi*, *P. inui, P. knowlesi*, and an unidentified *Plasmodium* species in Sabah, Malaysian Borneo [32]. *Anopheles introlatus* was hypothesised to be a vector in Selangor, Peninsular Malaysia, as Vythilingam et al. [33] discovered one out of 55 *An. introlatus* to be infected with only oocysts, but not sporozoites of *P. knowlesi*. In southern Vietnam where co-infection of *P. vivax* and *P. knowlesi* was predominant in humans and mosquitoes, *An. dirus* was shown to be the vector of *P. falciparum*, *P. vivax* and *P. knowlesi* using molecular methods [15]. Information derived from these studies was undeniably imperative for vector control but similar knowledge of vectors of *P. knowlesi* is lacking due to restricted sampling only in the Kapit District in Sarawak [28].

Molecular studies of malaria vectors have indicated that morphological keys cannot differentiate sibling species within a species complex and have revealed extensive cryptic speciation, with most nominal species in Southeast Asia being found to comprise species complexes [34–36]. The *Dirus* Complex alone, for example, consists of at least eight species [37, 38]. For the purpose of accurate identification, DNA barcodes such as the second internal transcribed spacer (ITS2) within the ribosomal DNA, mitochondrial cytochrome *c* oxidase subunits 1 and 2 (*cox*1 and *cox*2), and NADH dehydrogenase subunit 6 (*nad*6) were previously used to study the phylogeny of closely-related mosquito species [35, 39–45]. Accurate identification of vectors is especially important to inform malaria vector control programmes in Southeast Asia, where most *Anopheles* malaria vectors are comprised of species complexes [23, 36], and where malaria elimination is on the agenda [46]. It is therefore critical to utilise available molecular methods to precisely identify *Anopheles* mosquitoes found to be vectors for zoonotic malaria parasites.

The present study was aimed at incriminating the vector(s) of *P. knowlesi* and other malaria parasites in northern Sarawak (Lawas District), as well as providing an accurate identity of the incriminated vector(s). Lawas District was selected as the study site since 173 patients with knowlesi malaria had been admitted to the Lawas Hospital three years prior to the commencement of the study in 2014 (A. Ting, Lawas Hospital, personal communication).

## Methods

### Study site

**Fig. 1:**
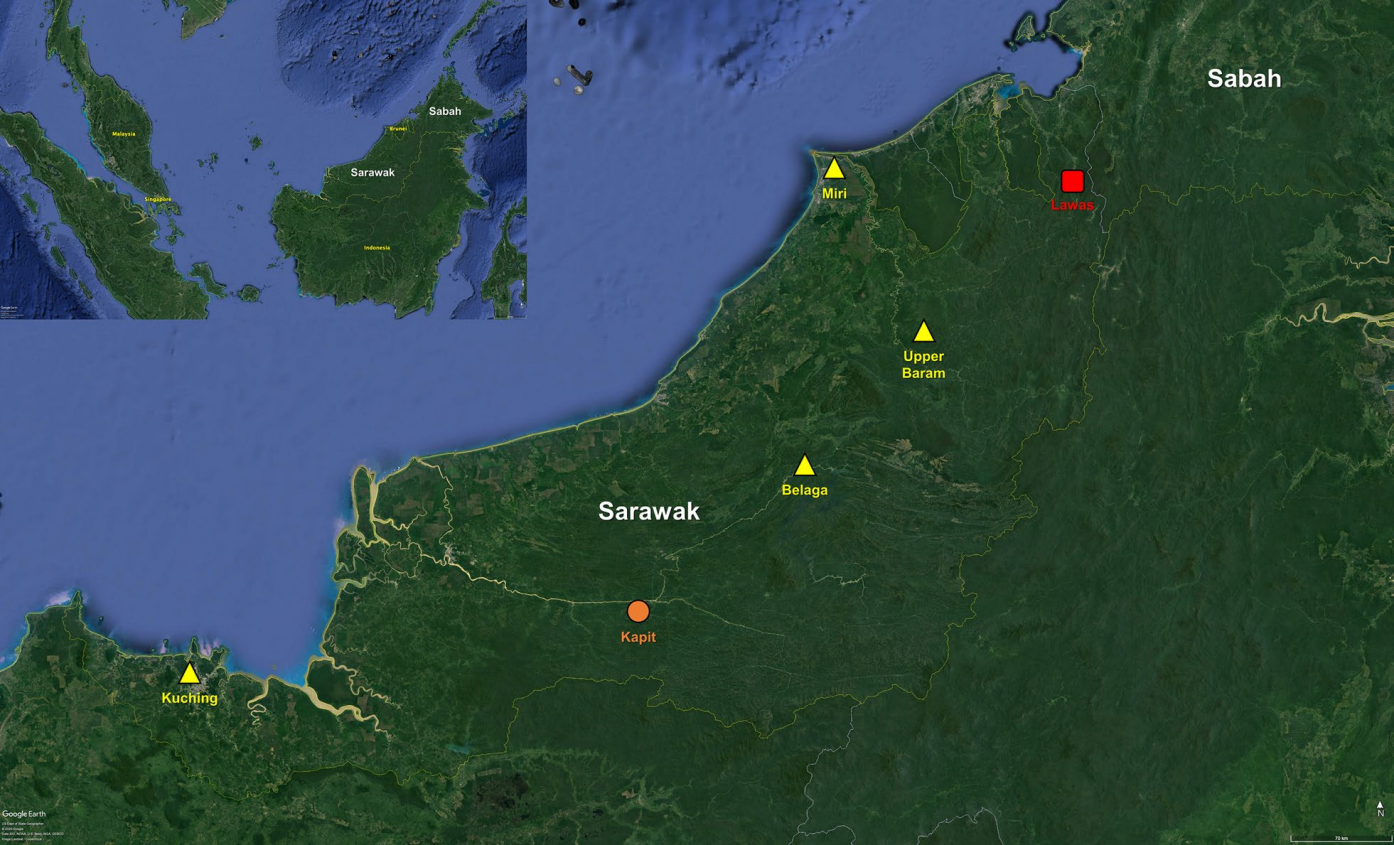
Locations of entomological surveys conducted previously and for the present study [74]. Human malaria sporozoites were discovered from dissected mosquitoes from these sites between 1951–1999 [24–27, 29] (triangles)*. Plasmodium knowlesi* and other simian malaria sporozoites were discovered from dissected mosquitoes and by nested PCR assays from this site between 2005–2006 [30] (circle). The study site for the present study is indicated by a square

The study was carried out in the Lawas District of northern Sarawak, Malaysian Borneo (Fig. 1). Lawas District is bordered by Brunei on its west, Sabah on its east, North Kalimantan on its south, and Labuan Federal Territory on its north. The first mosquito collection site was close to Long Tengoa village (DMS: 4° 37′ 5′′ N, 115° 20′ 23′′ E) which had 3 recent human cases of knowlesi malaria prior to mosquito collection in September 2014. The collection site is situated in a forested area *c.* 500 m eastward from the village at an elevation of about 100 m above sea level. The second collection was conducted in an abandoned army camp (4° 16′ 18′′ N, 115° 31′ 49′′ E) close to Long Luping, approximately 40 km southward from Long Tengoa. This camp is situated at an elevation of *c.* 650 m above sea level right beside a stream. The three patients who were admitted to Lawas Hospital with knowlesi malaria within three months prior to mosquito collection in May 2015 had spent time hunting near this camp site.

### Mosquito collection, identification and dissection

The first mosquito collection was carried out by 6 collectors for a period of 3 days in September 2014 in Long Tengoa village. The second collection at Long Luping was carried out by 5 collectors for 2 days in May 2015. All mosquitoes collected in Long Tengoa (18:00–23:00 h) were collected using the human landing catch method while those collected in Long Luping (day 1: 16:00–22:00 h; Day 2: 16:00–21:00 h) were collected using both the human landing catch method and the resting catch method. For the human landing catch method, all mosquitoes landing/biting on human bait were caught using a cylindrical specimen tube (18 mm in diameter × 50 mm in height) with moist tissue covering the bottom of the tube. Once a mosquito was caught, the opening of the tube was plugged with cotton wool and labelled according to the time of collection. The procedures for resting catch method were similar to that of the human landing catch method except that mosquitoes found resting under leaves were collected. The mosquitoes were then brought to the field laboratory for morphological identification. *Anopheles* mosquitoes were identified to the species/group level using taxonomic keys while non-anophelines were identified to the generic level [47–49]. Salivary glands of individual *Anopheles* mosquitoes were then carefully dissected and preserved in 1.5-ml microcentrifuge tubes (1 specimen/tube) containing 0.5 ml absolute ethanol. The dissection pins were wiped with dispensable 70% ethanol swabs after every dissection to prevent cross-contamination. Preserved salivary glands were transported to the Malaria Research Centre, Universiti Malaysia Sarawak, for further molecular analysis.

### DNA extraction and detection of *Plasmodium* species

Absolute ethanol preserving the salivary glands was first dried prior to DNA extraction. Genomic DNA of the dried salivary glands was extracted using DNeasy^®^ Blood and Tissue Kit (Qiagen, Hilden, Germany) according to the manufacturer’s protocol. The eluted samples were kept at 4 °C until required. Extracted DNA samples were initially subjected to nested PCR assays for the detection of *Plasmodium* DNA based on the *SSU* rRNA genes using the *Plasmodium*-specific primers rPLU3 and rPLU4 [50]. Subsequently, *Plasmodium*-positive samples were tested using a species-specific PCR assays to identify *P. coatneyi*, *P. cynomolgi* and *P. knowlesi* [1]. Additionally, new PCR primers were developed for *P. fieldi* (PfldF3: 5′-GAT CTT TTT TTG TTT CGG CAT TGA A-3′; PfldR3: 5′-AAG GCA CTG AAG GAA GCA ATC TAA GAG TTT-3′) and *P. inui* (PinF5: 5′-GTA TCG ACT TTG TGC GCA TTT TTC TAC-3′; INAR3: 5′-GCA ATC TAA GAG TTT TAA CTC CTC-3′) with optimum annealing temperatures at 60 °C and 62 °C, respectively. The new primers were found to have higher specificity (data not shown) compared to the *P. fieldi-*specific and *P. inui-*specific primers developed previously [1]. The processes of genomic DNA extraction, PCR mastermix preparation, pipetting of template for primary PCR, and pipetting of template for nested PCR were each conducted in a different room, using filtered pipette tips and micropipettes dedicated to each room to prevent cross-contamination. These PCR assays were repeated for samples that were positive for the initial nested PCR assays and only samples which were consistently positive on both occasions were subjected to sequencing of *Plasmodium SSU* rRNA genes.

### Generating *Plasmodium SSU* rRNA gene amplicons

The *SSU* rRNA gene fragments were amplified by semi-nested PCR assays in order to increase the DNA yield prior to cloning. Nest 1 PCR amplification was performed using primers rPLU1 and rPLU5 as previously described [50]. Then, PCR product from Nest 1 was used as DNA template for the Nest 2 PCR amplification using the Phusion High-Fidelity DNA Polymerase (Thermo Fisher Scientific, Waltham, USA). PCR primers rPLU3 and rPLU5 [50] were used with an annealing temperature of 68 °C.

### Generating *Anopheles cox*1 and ITS2 region amplicons

Both the *cox*1 and ITS2 regions of the vectors were amplified by PCR assays using the Phusion High-Fidelity DNA Polymerase (Thermo Fisher Scientific) according to the manufacturer’s protocol. To amplify a 748 bp-long *cox*1 region, PCR primers AnCOX1F (5′-GGA ATG GAT GTW GAT ACW CGA GC-3′) and AnCOX1R (5′-CCT AAA TTT GCT CAT GTT GCC-3′) were designed with the annealing temperature of 65 °C. For the ITS2 region, DNA amplification was performed using the PCR primers 5.8SF and 28SR as previously described by Paredes-Esquivel et al. [35].

### Cloning and sequencing

Generated amplicons were ligated with pCR^®^-Blunt Vector (Invitrogen, Carlsbad, USA) and transformed into One Shot^®^ TOP10 Chemically Competent *E. coli* (Invitrogen), according to the Zero Blunt^®^ PCR Cloning Kit (Invitrogen) protocol. *Escherichia coli* transformed colonies were screened with PCR using M13 primers, for the presence of target DNA insert and plasmids containing the inserts were purified using the PureLink^®^ Quick Plasmid Miniprep Kit (Thermo Fisher Scientific). DNA sequencing of plasmid DNA was conducted according to the BigDye^®^ Terminator v3.1 Cycle Sequencing Kit (Thermo Fisher Scientific) protocol, using M13 primers, internal primers rPLU2 and rPLU6 for the *Plasmodium SSU* rRNA inserts, and internal primers JIBF (5′-CTA GTG TGC TTC CCA TGG AGA TAG-3′) and JIBR (5′-CAC ACC CAA CCC AAT AAA AAT TG-3′) for ITS2 inserts.

### Phylogenetic analysis

For the ITS2 region, the central repeat region contained within it was identified using the Tandem Repeats Finder software (https://tandem.bu.edu/trf/trf.html) as described previously and trimmed [35, 51]. The trimmed sequences were then aligned for phylogenetic analysis. No trimming was done on the *SSU* rDNA and *cox*1 sequences prior to alignment. Reference sequences obtained from GenBank [35, 40, 52–54] are listed in Additional file 1: Table S1 and Additional file 2: Table S2. Multiple sequence alignments were performed using the default parameters of ClustalW within the LaserGene 7.1 programme (DNASTAR; Lasergene, Inc., Madison, WI, USA). The best nucleotide substitution models were calculated using MEGA 7.0.21 and the models with the lowest Bayesian information criterion (BIC) were selected [55, 56]. Subsequently, phylogenetic trees were constructed by the Maximum Likelihood (ML) method using MEGA 7.0.21 with bootstrap values calculated from 1000 replicates [57].

## Results

### Species composition of collected mosquitoes

A total of 192 mosquitoes were collected from Long Tengoa (*n* = 86) and Long Luping (*n* = 106) (Table 1). Based on morphological characteristics, mosquitoes of the genus *Anopheles* were the most abundant (*n* = 65; 34%), followed by *Culex* spp. (*n* = 41; 21%), *Aedes* spp. (*n* = 40; 21%), *Armigeres* spp. (*n* = 31; 16%), *Mansonia* spp. (*n* =  7; 4%), *Malaya* spp. (*n* = 6; 3%) and *Uranotaenia* spp. (*n* = 2; 1%).

**Table 1:** Genera/species of mosquitoes collected and identified morphologically in Lawas District, Malaysia

Genera/species	Number of mosquitoes		
	Long Tengoa	Long Luping	Total
*An. balabacensis*	0	31	31
*An. latens*	1	1	2
*An. barbirostris* (*s.l*.)	0	18	18
*An. donaldi*	3	0	3
*An. letifer*	5	0	5
*An. roperi*	1	0	1
*An. umbrosus*	4	0	4
*An. tessellatus*	0	1	1
*Aedes* spp.	9	31	40
*Armigeres* spp.	31	0	31
*Culex* spp.	31	10	41
*Malaya* spp.	0	6	6
*Mansonia* spp.	1	6	7
*Uranotaenia* spp.	0	2	2
Total	86	106	192

A higher proportion of culicines was found in Long Tengoa (*n* = 72; 84%), while *Anopheles* mosquitoes predominated in Long Luping (*n* = 51; 48%). Most *An. balabacensis* (*n* = 31) and *An. barbirostris* (*s.l*.) (*n* = 18) mosquitoes were caught at Long Luping while all specimens of the Umbrosus Group (*An. letifer*, *An. roperi* and *An. umbrosus*) were collected in Long Tengoa, and these were not collected in Long Luping (Table 1).

### *Plasmodium* species in *Anopheles* mosquitoes

DNA extracted from the salivary glands of 65 *Anopheles* mosquitoes was subjected to nested PCR assays and *Plasmodium* DNA was only detected in samples derived from 10 *An. balabacensis* and 6 *An. barbirostris* (*s.l*.) collected in Long Luping. Of 31 *An. balabacensis*, 9 (29.0 %) were found to carry *P. knowlesi* and other simian malaria parasites while 5 (27.8 %) *An. barbirostris* (*s.l*.) were found to be infected with *P. knowlesi* only (Table 2). The identity of the species of *Plasmodium* could not be determined for 2 of the *Plasmodium*-positive samples by using the species-specific PCR primers for *P. coatneyi*, *P. cynomolgi*, *P. inui*, *P. fieldi* and *P. knowlesi*.

**Table 2:** Summary of results of PCR assays for *Plasmodium*-positive samples from Long Luping

Species	No. of samples analysed by PCR assays	No. of positive samples		Sample ID
		Genus-specific PCR assay	Species-specific PCR assays^a^	
*An. balabacensis*	30	10	1: No species identified	LW101
			6: *Pk*	LW31, LW32, LW49, LW50, LW51, LW59
			1: *Pcy* + *Pk*	LW45
			1: *Pcy* + *Pfld* + *Pin*	LW67
			1: *Pin*	LW74
*An. barbirostris* (*s.l*.)	18	6	1: No species identified	LW38
			5: *Pk*	LW44, LW47, LW48, LW57, LW58

^a^*Pcy-*, *P. cynomolgi*; *Pfld-*, *P. fieldi*; *Pin-*, *P. inui*; *Pk-*, *P. knowlesi*

**Fig. 2:**
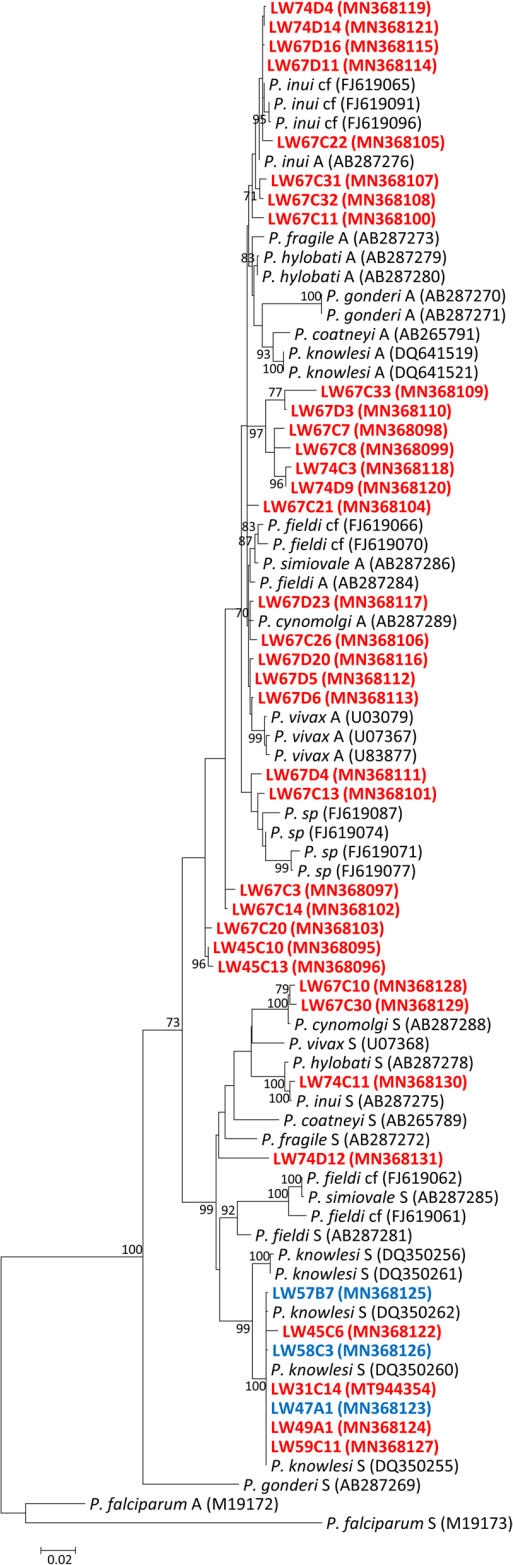
Phylogenetic tree for *Plasmodium* spp. based on the *SSU* rRNA genes using the ML method. The analysis was based in the Tamura 3-parameter + G + I substitution model. GenBank accession numbers are given in parentheses; letters ‘A’ and ‘S’ represent the different isoforms of the *SSU* rRNA gene. Only bootstrap values > 70% are shown at the nodes. Blue and red colours represent parasites obtained from *An. barbirostris (s.l.)* and *An. balabacensis*, respectively

All 14 samples which tested positive for at least one simian malaria parasite were subjected to another round of semi-nested PCR to obtain a longer fragment of the *SSU* rRNA for sequencing. Amplicons could not be generated for 5 (3 *An. balabacensis* and 2 *An. barbirostris* (*s.l*.)) of the samples infected with *P. knowlesi* only. For the 9 samples where amplicons were successfully obtained, cloned and transformed into chemically competent *E. coli*, colony PCR was conducted on at least 10 colonies. Colonies originating from the same transformation which produced amplicons of slightly different sizes (1500–2000 bp) were subjected to sequencing. As a result, a total of 38 *SSU* rRNA fragments were cloned and sequenced from these *Anopheles* mosquitoes (6 *An. balabacensis* and 3 *An. barbirostris* (*s.l*.) which were *Plasmodium*-positive by nested PCR assays. The phylogenetic tree (Fig. 2) constructed using the *Plasmodium* A-type and S-type *SSU* rRNA sequences showed that *An. balabacensis* were harbouring sporozoites of *P. cynomolgi* (LW67), *P. inui* (LW67 and LW74) and *P. knowlesi* (LW45, LW59, LW31 and LW49) in their salivary glands, while *An. barbirostris* (*s.l*.) carried only *P. knowlesi* (LW47, LW57 and LW58). Three *An. balabacensis* (LW45, LW67 and LW74) were shown to carry more than 1 species of *Plasmodium* while the other 2 *An. balabacensis* (LW31 and LW49) were infected with *P. knowlesi* only. Multiple *SSU* rRNA sequences derived from the salivary glands of mosquitoes LW45, LW67 and LW74 formed several distinct clades instead of grouping with any of the clades formed by the reference sequences (Fig. 2). This indicates that *An. balabacensis* is not only harbouring sporozoites of *P. cynomolgi*, *P. inui* and *P. knowlesi*, but probably sporozoites of unknown species of *Plasmodium*.

**Fig. 3:**
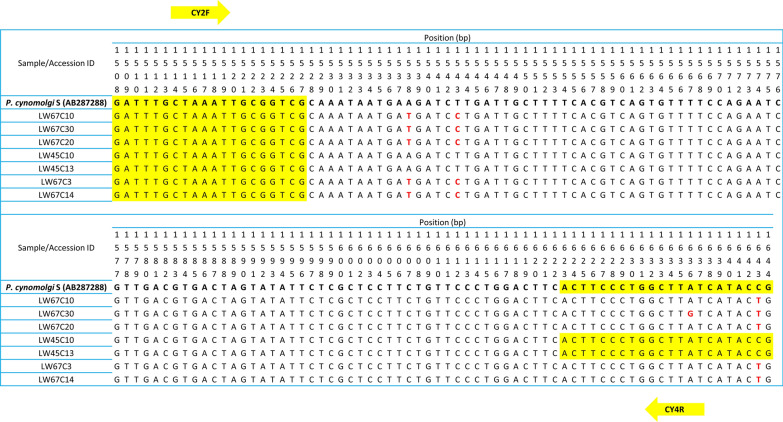
Alignment of clones which could potentially be detected by PCR with primers CY2F and CY4R. Primer-binding sites are highlighted in yellow and SNPs are represented in red

Despite detecting *P. cynomolgi* and *P. fieldi* by PCR in LW45 and LW67, respectively, we were unable to obtain *SSU* rRNA sequences of these two species of *Plasmodium* from the clones that were sequenced (Fig. 2). Binding sites for *P. cynomolgi–*specific and -*P. fieldi-*specific primers were subsequently searched for in sequences isolated from LW45 and LW67 to confirm their specificity in detecting the species they were developed to identify. Clones LW45C10 and LW45C13 were found to have fully complementary binding sites for the *P. cynomolgi*-specific primers (CY2F + CY4R) although they did not form a clade with *P. cynomolgi* in the phylogenetic tree (Fig. 2). CY2F binding site was also discovered to be fully conserved in several other clones isolated from LW67 (C3, C10, C14, C20 and C30) while one and/or two SNPs were present for primer CY4R (Fig. 3). As the SNPs were not near to the 3′-end of the primer binding site, CY2F + CY4R could potentially amplify a 137-bp fragment from these aligned clones in a PCR. On the other hand, *P. fieldi*-specific primer sequences were not conserved in any of the clones isolated from LW67. This suggests that the *P. fieldi* detected by PCR was not recovered among the 23 clones sequenced.

### Molecular characterisation of *Anopheles* mosquitoes

**Fig. 4:**
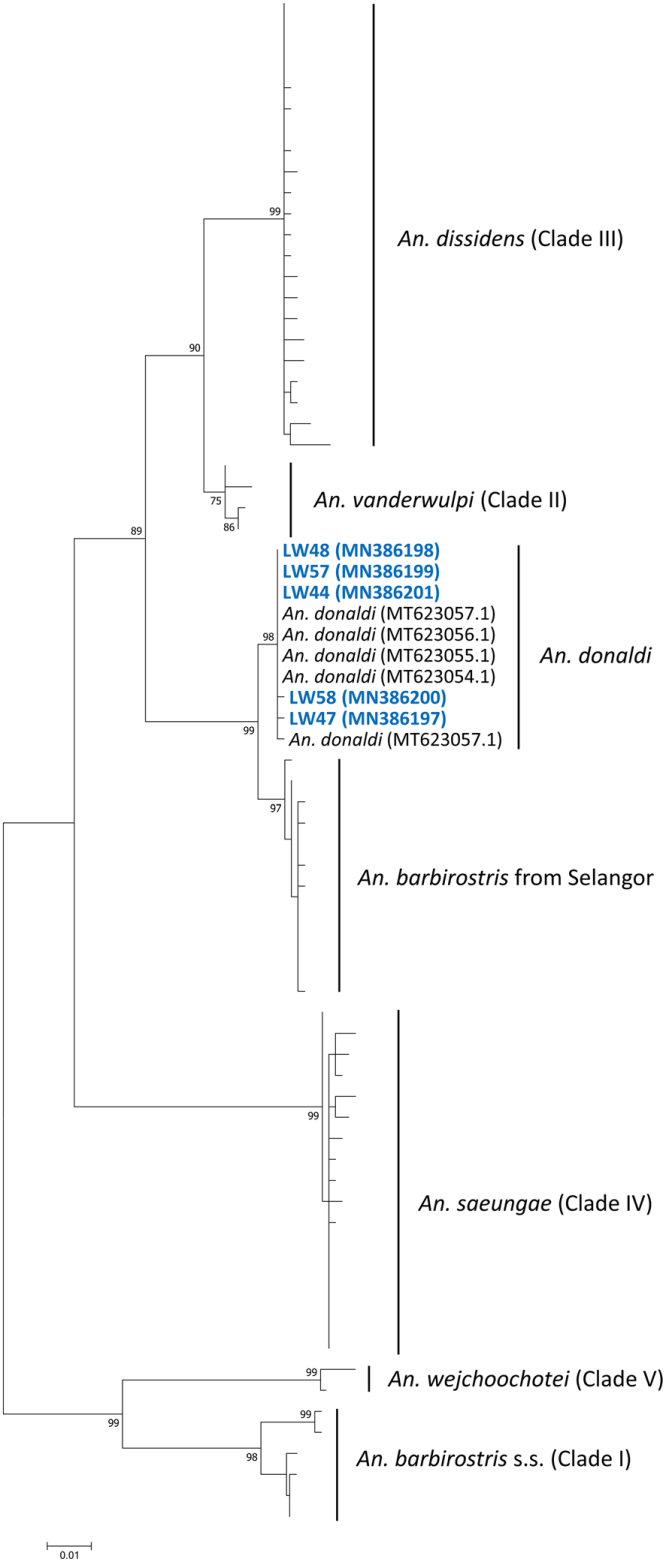
Phylogenetic tree for *Anopheles* spp. based on the ITS2 region using the ML method. The analysis was based in the Kimura 2-parameter + G substitution model. Only bootstrap values > 70% are shown on the nodes. GenBank accession numbers are given in parentheses. Blue colour represents *An. barbirostris* (*s.l*.) collected in Lawas District

**Fig. 5:**
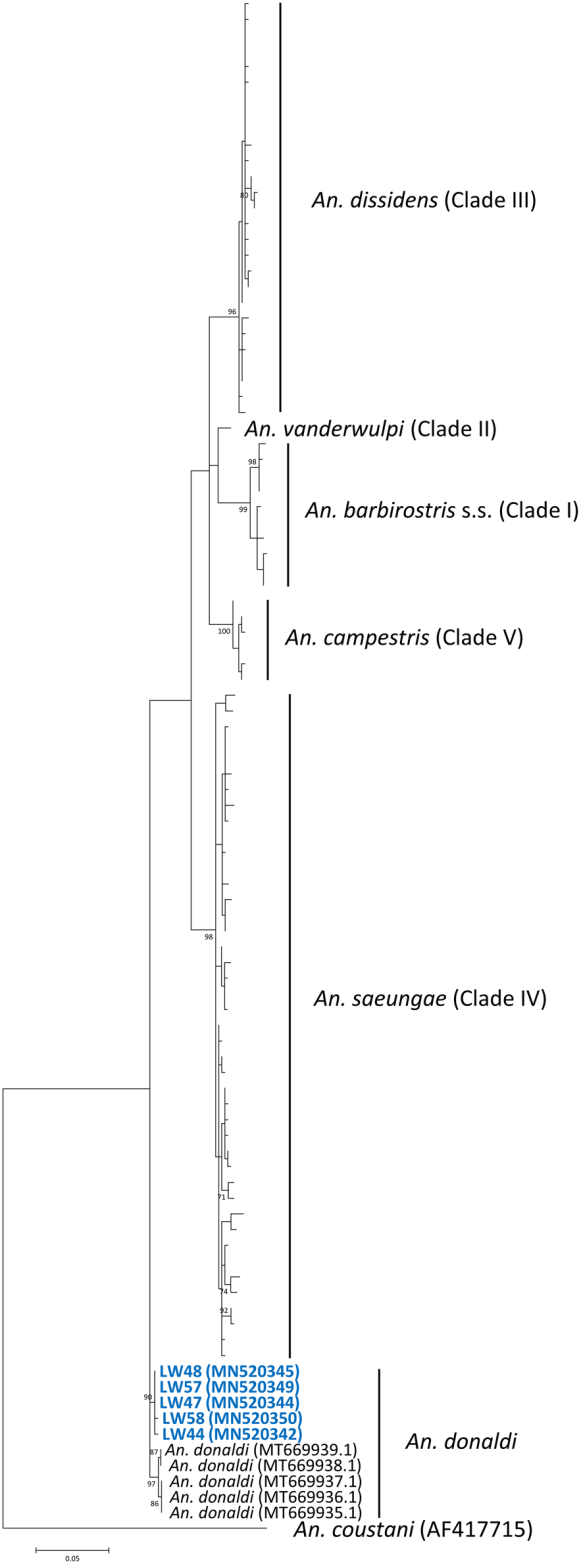
Phylogenetic tree for *Anopheles* spp. based on the *cox*1 gene using the ML method. The analysis was based in the Tamura 3-parameter + G + I substitution model. Only bootstrap values > 70% are shown on the nodes. GenBank accession numbers are given parentheses. Blue colour represents *An. barbirostris* (*s.l*.) collected in Lawas District

The ITS2 region and *cox*1 gene (for multiple sequence alignment of *cox*1 see Additional file 3: Alignment S1, Additional file 4: Alignment S2) of *Anopheles* mosquitoes carrying simian malaria parasites were further sequenced to confirm their identities. Phylogenetic analysis of the ITS2 region and *cox*1 gene of *An. barbirostris* (*s.l*.) from Thailand, West Sumatra, West Java, South Kalimantan, and Sabah showed 6 distinct clades (Figs. 4, 5). The phylogenetic trees constructed using the ML method showed that sequences derived from *An. barbirostris* (*s.l*.) collected from the Lawas District, Sarawak, clustered together with *An. donaldi* (Figs. 4, 5) from Sabah and *An. barbirostris* (*s.l*.) (Fig. 4) from Selangor, Peninsular Malaysia. This indicates that the *P. knowlesi-*positive *An. barbirostris* (*s.l*.) collected in the present study are *An. donaldi.*

**Fig. 6:**
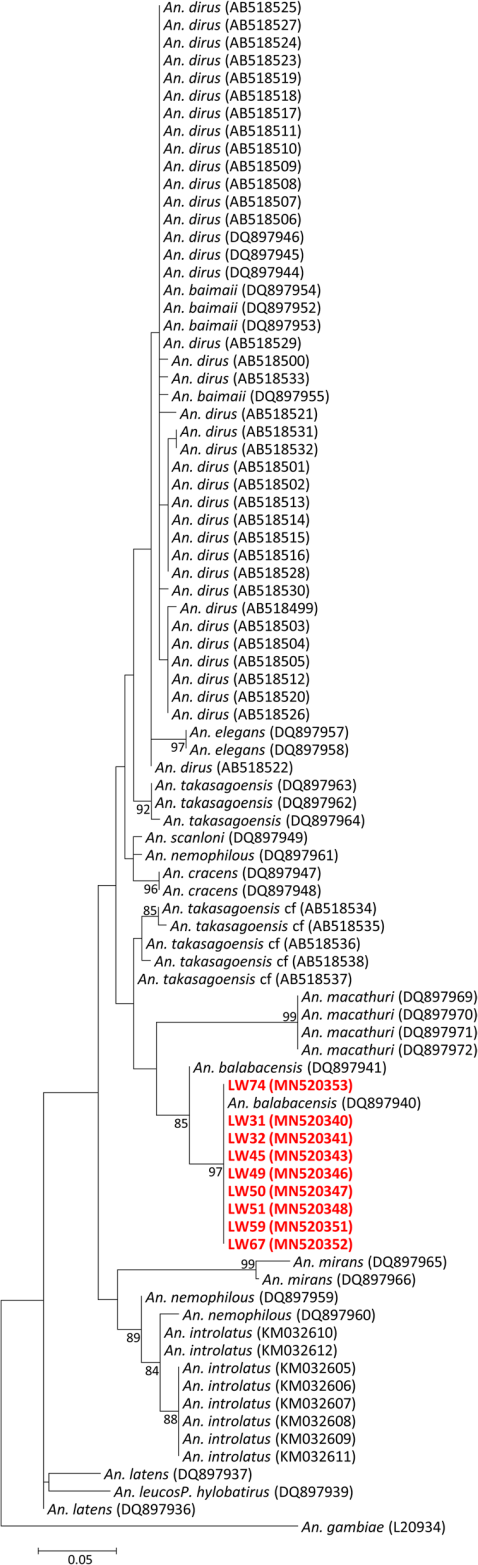
Phylogenetic tree for *Anopheles* spp. based on the *cox*1 gene using the ML method. The analysis was based in the Tamura 3-parameter + G substitution model. Only bootstrap values > 70% are shown on the nodes. GenBank accession number are given in parentheses. Red colour represents *An. balabacensis* collected in Lawas District

The *cox*1 sequences of *An. balabacensis* obtained in this study showed close phylogenetic relationships (Fig. 6) with the other *An. balabacensis* sequences. Those collected from this study in Lawas District, Sarawak, clustered together with the *An. balabacensis* in eastern Sabah, Malaysian Borneo (GenBank: DQ897940), forming a sister clade to the *An. balabacensis* from South Kalimantan, Indonesian Borneo (GenBank: DQ897941).

## Discussion

### Identification of *Plasmodium*

Sporozoites of *P. knowlesi* and other simian malaria parasites were identified by nested PCR assays in *An. balabacensis* (*n* = 9) and *An. barbirostris* (*s.l.*) (*n* = 5) (subsequently identified following molecular characterisation as *An. donaldi*). The specimens of *An. balabacensis* and *An. donaldi* examined had sporozoite infection rates of 29.0% and 27.8% respectively, which are exceedingly high compared to other vector studies in Malaysia. In the neighbouring Malaysian Borneo state of Sabah, *An. balabacensis* was identified as the vector for *P. knowlesi* with sporozoite rates ranging from 1.03 to 3.42% at three different sites [32]. Infection rates of < 2.00% were reported among vectors in central Sarawak and among *An. cracens* in Peninsular Malaysia [29, 31, 58]. The only other study known to have found a comparable sporozoite rate was a study done in Palawan, Philippines, where 29.4% of the examined *An. balabacensis* had sprorozoites [59]. The high sporozoite rates of the vectors could be attributed to the site of collection as well, which is consistent with the finding of the previous study involving *An. latens* in Kapit, Sarawak [58], where it was found that the sporozoite rate was highest in the forest, followed by the rates in the farm at the forest-fringe, and in the long house. Long Luping is an abandoned army camp which is far away from any human settlement. It is potentially a foraging site for the macaques due to the banana trees that grow around its perimeter. No vector control activities have been carried out since the army camp was abandoned and macaques were sighted at the site during the entomological surveys, suggesting that macaques and mosquitoes could forage and breed, respectively, undisturbed in this site.

The high infection rates could also be due to the way sampling was conducted compared to other reported studies [4, 30–32, 60, 61]. First, the present method recovers both sides of the salivary glands without rupturing any to check for sporozoites, increasing the yield of extracted *Plasmodium* DNA. Secondly, the present method does not retain the head of the specimens which might contain inhibitors which would disrupt PCR assays. PCR inhibitors were previously found to be present in the heads of *Culex pipiens* and *An. punctipennis* which caused false negative results in detection of *Wolbachia pipientis* in samples tested [62]. The efficiency of the PCR assay was later restored when specimens were decapitated prior to DNA extraction. Although we had high sporozoite infection rates by nested PCR assays, we failed to generate the longer (1500–2000 bp) *Plasmodium SSU* rDNA amplicons from five samples that were malaria-positive by nested PCR assays. This could be caused by the very low number of sporozoites present in the salivary glands of the mosquitoes and the low sensitivity of the PCR assay to amplify long fragments from a sample with low template concentration.

### Diversity and density of *Plasmodium* infection in vectors

In line with the discovery of at least seven *Plasmodium* species infecting long-tailed macaques in Sarawak [63], it is unsurprising that multiple unidentified *Plasmodium* species were recovered from *An. balabacensis* in this study (Fig. 2). However, the low quantity of DNA extracted from the salivary glands prevented the sequencing of another gene, such as the mitochondrial genome, which would have been necessary to determine whether these are indeed novel species of *Plasmodium*. It is highly likely that unidentified *Plasmodium* species co-infected the vectors when they fed on macaques which are known to host a diverse range of species of *Plasmodium* [1, 4, 63, 64].

Accurate identification of *Plasmodium* by PCR might also be impeded when uncharacterised *Plasmodium*, such as those found in sample LW45 (C10 and C13), could be detected by *P. cynomolgi*-specific primers (Fig. 3). This demonstrates the need for the sequencing of a considerable genomic locus length for proper identification of the species of *Plasmodium*. On the other hand, the unsuccessful attempts to sequence *P. fieldi* from sample LW67, and *P. cynomolgi* from LW45, could be due to the low density of *P. fieldi* and *P. cynomolgi*, respectively, among the other *Plasmodium* co-infecting each of these mosquitoes. As the PCR amplification prior to cloning amplifies the *SSU* rRNA genes of all *Plasmodium* species indiscriminately, the scarcity of any species of *Plasmodium* DNA in the sample reduces the chance of its amplicon being produced during PCR amplification. The difficulty in obtaining sequencing data of genes of *Plasmodium* derived from vectors is probably the main reason why previous studies on vectors of knowlesi malaria have only used nested PCR assays [15, 30, 32, 33, 61] and for the paucity of studies describing the diversity and density of *Plasmodium* infection in vectors [31, 60]. With an increasing number of zoonotic malaria infections worldwide [65–67], epidemiological studies of these inadequately studied species within human populations that come into close contact with macaques during activities in the forest and forest-fringe will also be required to monitor potential host-switch events.

### Molecular characterisation of vectors and implications for vector control in Sarawak

Phylogenetic analyses confirmed that the *Plasmodium*-positive mosquitoes from Lawas, Sarawak, identified morphologically as *An. balabacensis* were *An. balabacensis* whereas those identified as *An. barbirostris* (*s.l*.) were *An. donaldi*. *Anopheles balabacensis* has been incriminated as a vector for *P. knowlesi* in Sabah, Malaysian Borneo and belongs to the *Leucosphyrus* Group which has been long thought to be the only species group capable of transmitting *P. knowlesi* in natural settings [4, 15, 30–32, 60, 61]. *Anopheles kochi* from the Kochi Group was suspected as a vector due to its high susceptibility to *P. knowlesi* infection under experimental conditions and its simiophilic biting behaviour but the parasite was never recovered from any *An. kochi* collected in the natural environment [4, 68, 69]. DNA of *P. knowlesi* was detected by PCR assays in 2019 from DNA extracted from the carcasses of *An. donaldi* in Sabah, Malaysian Borneo and from *An. sundaicus* in the Nicobar and Andaman Islands of India, [61, 70] whereas the *Plasmodium* DNA detected in the present study in Sarawak, Malaysian Borneo were recovered from the salivary glands of mosquitoes. In both Sabah and Sarawak, more detailed studies need to be conducted on the bionomics of *An. donaldi* and *An. balabacensis* to provide data for the implementation of appropriate vector control. Species-specific molecular assays should also be designed and utilised in future vector incrimination studies for this species range in order to correctly identify malaria-infective mosquitoes.

The incrimination of *An. balabacensis* and *An. donaldi* as novel vectors for *P. knowlesi* in northern Sarawak calls for re-evaluation of current and future vector control methods in the state. Detailed studies first need to be undertaken to determine the feeding behaviour and host preference of these vectors. The main vector control methods currently adopted by the Sarawak State Health Department for malaria control are the provision of insecticide treated bednets and the regular spraying of residual insecticide on houses in malarious areas. These would be of limited value against *An. latens* in central Sarawak which are mainly exophagic and acrodendrophilic [58]. Further studies need to be undertaken on *An. donaldi* and *An. balabacensis* in Lawas to determine the effectiveness of providing bednets to people. From the aspect of insecticide-based preventative measures, future insecticide resistance surveys should include both *An. balabacensis* and *An. donaldi* to ensure that the insecticide used would still be efficient in killing these vectors. As one of the main vector control methods currently adopted by the Sarawak State Health Department is the regular spraying of residual insecticide on houses in malarious area, spraying could also be considered for uninhabited buildings like the army camp in Long Luping, where human presence is intermittent. Apart from insecticides, clustered regularly interspaced short palindromic repeat (CRISPR)-based gene drive has been recently suggested as one possible method for the control of *P. knowlesi* vector(s) [71]. Gene drive is a biotechnology method used to increase the spread of a genetic trait (e.g. mosquito sterility/mosquito immunity against *Plasmodium* infection) into the wild population. As the system relies on the ability of the nuclease to recognise a specific nucleotide sequence and cause a double-stranded break on the chromosome [72], it is imperative that the genomes of the vectors of *P. knowlesi* are readily available for researchers in the field. Besides *An. dirus* which is a member of the *Leucosphyrus* Group, no other *An. leucosphyrus* (*s.l*.) and *An. barbirostris* (*s.l*.) genomes have been sequenced [73]. Genomic data of vectors of *P. knowlesi* incriminated so far in Peninsular Malaysia, Malaysian Borneo and Vietnam will be crucial in the development of a gene drive since it is an extremely species-specific vector control method [72]. Novel methods of vector control are clearly needed, and while waiting for these to be developed, personal protection and avoidance of being bitten by mosquitoes need to be advocated during public health promotion exercises as methods for the prevention and control of zoonotic malaria.

## Conclusions

The malaria-positive *An. balabacensis* in Lawas were phylogenetically indistinguishable from *An. balabacensis* in eastern Sabah, Malaysian Borneo and south Kalimantan, Indonesian Borneo. Phylogenetic analyses also indicated that the *Plasmodium*-positive mosquitoes identified morphologically as *An. barbirostris* (*s.l*.) in Lawas are *An. donaldi*. *Anopheles balabacensis* has been incriminated as a vector of *P. knowlesi* and other simian malaria parasites, and *An. donaldi* as a vector of *P. knowlesi* in Lawas, northern Sarawak in Malaysian Borneo. Previously *An. latens* had been incriminated as the vector for *P. knowlesi* in Kapit, central Sarawak, so these two species represent novel vectors for zoonotic malaria in Sarawak.

## Supplementary information

**Additional file 1: Table S1.** Plasmodium *SSU* rDNA sequences and their GenBank accession numbers.

**Additional file 2: Table S2.** *cox*1 and ITS2 sequences of *Anopheles* mosquitoes and their GenBank accession numbers.

**Additional file 3: Alignment S1.** Multiple sequence alignment of the *cox*1 gene of *An. barbirostris* (*s.l*.) and an outgroup, *An. coustani*.

**Additional file 4: Alignment S2.** Multiple sequence alignment of the *cox*1 gene of *An. leucosphyrus* (*s.l*.) and an outgroup, *An. gambiae*.

## Data Availability

All data generated or analysed during this study are included in this published article and its additional files. Sequences were deposited in GenBank under the accession numbers listed in Additional files 1 and 2.

## Abbreviations

*SSU* rRNA: Small subunit ribosomal RNA
*cox*1: Cytochrome *c* oxidase subunit 1 gene
ITS2: Internal transcribed spacer 2 region
DNA: Deoxyribonucleic acid
PCR: Polymerase chain reaction
