# Supplementary material for: New vectors in northern Sarawak, Malaysian Borneo, for the zoonotic malaria parasite, Plasmodium knowlesi
Source: Parasit Vectors. 2020 Sep 15;13:472. doi: 10.1186/s13071-020-04345-2 (PMC7490903; doi:10.1186/s13071-020-04345-2)
Supplement: Supplementary file 1 — Additional file 1: Table S1. Plasmodium SSU rDNA sequences and their GenBank accession numbers. [file 13071_2020_4345_MOESM1_ESM.docx]

**Additional file 1: Table S1.** *Plasmodium SSU* rDNA sequences and their GenBank accession numbers.

| Species/Sample no. | GenBank Acc No. | References |
| --- | --- | --- |
| *P. gonderi* | AB287269 | [1] |
|  | AB287270 |  |
|  | AB287271 |  |
| *P. fragile* | AB287272 |  |
|  | AB287273 |  |
| *P. coatneyi* | AB265789 |  |
|  | AB265791 |  |
| *P. inui* | AB287275 |  |
|  | AB287276 |  |
| *P. hylobati* | AB287278 |  |
|  | AB287279 |  |
|  | AB287280 |  |
| *P. fieldi* | AB287281 |  |
|  | AB287284 |  |
| *P. simiovale* | AB287285 |  |
|  | AB287286 |  |
| *P. cynomolgi* | AB287288 |  |
|  | AB287289 |  |
| *P. knowlesi* | DQ641519 | Unpublished |
|  | DQ641521 |  |
|  | DQ350255 |  |
|  | DQ350256 |  |
|  | DQ350260 |  |
|  | DQ350261 |  |
|  | DQ350262 |  |
| *P. fieldi* cf | FJ619062 |  |
|  | FJ619066 |  |
|  | FJ619070 |  |
| *P. sp* | FJ619071 |  |
|  | FJ619074 |  |
|  | FJ619077 |  |
|  | FJ619087 |  |
| *P. inui* cf | FJ619065 |  |
|  | FJ619091 |  |
|  | FJ619096 |  |
| *P. vivax* | U83877 | Unpublished |
|  | U07367 | [2] |
|  | U07368 |  |
|  | X13926 | [3] |
| *P. falciparum* | M19172 | [4] |
|  | M19173 |  |
| LW45C10 | MN368095 | Present study |
| LW45C13 | MN368096 |  |
| LW67C3 | MN368097 |  |
| LW67C7 | MN368098 |  |
| LW67C8 | MN368099 |  |
| LW67C11 | MN368100 |  |
| LW67C13 | MN368101 |  |
| LW67C14 | MN368102 |  |
| LW67C20 | MN368103 |  |
| LW67C21 | MN368104 |  |
| LW67C22 | MN368105 |  |
| LW67C26 | MN368106 |  |
| LW67C31 | MN368107 |  |
| LW67C32 | MN368108 |  |
| LW67C33 | MN368109 |  |
| LW67D3 | MN368110 |  |
| LW67D4 | MN368111 |  |
| LW67D5 | MN368112 |  |
| LW67D6 | MN368113 |  |
| LW67D11 | MN368114 |  |
| LW67D16 | MN368115 |  |
| LW67D20 | MN368116 |  |
| LW67D23 | MN368117 |  |
| LW74C3 | MN368118 |  |
| LW74D4 | MN368119 |  |
| LW74D9 | MN368120 |  |
| LW74D14 | MN368121 |  |
| LW45C6 | MN368122 |  |
| LW47A1 | MN368123 |  |
| LW49A1 | MN368124 |  |
| LW57B7 | MN368125 |  |
| LW58C3 | MN368126 |  |
| LW59C11 | MN368127 |  |
| LW67C10 | MN368128 |  |
| LW67C30 | MN368129 |  |
| LW74C11 | MN368130 |  |
| LW74D12 | MN368131 |  |
| LW31C14 | MT944354 |  |

**References**

1. Nishimoto Y, Arisue N, Kawai S, Escalante AA, Horii T, Tanabe K, et al. Evolution and phylogeny of the heterogeneous cytosolic *SSU* rRNA genes in the genus *Plasmodium*. Mol Phylogenet Evol. 2008;47:45–53.

2. Li J, Wirtz RA, McConkey GA, Sattabongkot J, McCutchan TF. Transition of *Plasmodium vivax* ribosome types corresponds to sporozoite differentiation in the mosquito. Mol Biochem Parasitol. 1994;65:283–9.

3. Waters AP, Mccutchan TF. Partial sequence of the asexually expressed SU rRNA gene of *Plasmodium vivax*. Nucleic Acids Res. 1989;17:2135.

4. McCutchan TF, de la Cruz VF, Lal AA, Gunderson JH, Elwood HJ, Sogin ML. Primary sequences of two small subunit ribosomal RNA genes from *Plasmodium falciparum*. Mol Biochem Parasitol. 1988;28:63–8.
