# Supplementary material for: New vectors in northern Sarawak, Malaysian Borneo, for the zoonotic malaria parasite, Plasmodium knowlesi
Source: Parasit Vectors. 2020 Sep 15;13:472. doi: 10.1186/s13071-020-04345-2 (PMC7490903; doi:10.1186/s13071-020-04345-2)
Supplement: Supplementary file 2 — Additional file 2: Table S2. cox1 and ITS2 sequences of Anopheles mosquitoes and their GenBank accession numbers. [file 13071_2020_4345_MOESM2_ESM.docx]

**Additional file 2: Table S2.** CO1 and ITS2 sequences of *Anopheles* mosquitoes and their GenBank accession numbers.

| Species Subgroup | Species | GenBank Acc No. | | Geographic distribution | Reference |
| --- | --- | --- | --- | --- | --- |
|  |  | CO1 | ITS2 |  |  |
| Barbirostris Subgroup | *An. barbirostris s.s.* | EU797194 | - | Indonesia | [1] |
|  |  | EU797195 | EU812759 |  |  |
|  |  | EU797196 | EU812760 |  |  |
|  |  | EU797197 | - |  |  |
|  |  | EU797198 | EU812761 | Thailand |  |
|  |  | EU797199 | EU812764 |  |  |
|  |  | EU797200 | - |  |  |
|  |  | EU797201 | - |  |  |
|  |  | EU797202 | EU812762 |  |  |
|  |  | EU797203 | EU812763 |  |  |
|  | *An. vanderwulpi* | EU797204 | EU812765 | Indonesia |  |
|  |  | EU797204 | EU812766 |  |  |
|  |  | EU797204 | EU812767 |  |  |
|  |  | EU797204 | EU812768 |  |  |
|  | *An. dissidens* | EU797205 | EU812781 | Thailand |  |
|  |  | EU797205 | EU812769 |  |  |
|  |  | EU797205 | EU812771 |  |  |
|  |  | EU797205 | EU812773 |  |  |
|  |  | EU797205 | EU812774 |  |  |
|  |  | EU797205 | EU812776 |  |  |
|  |  | EU797205 | EU812777 |  |  |
|  |  | EU797206 | EU812778 |  |  |
|  |  | EU797206 | EU812783 |  |  |
|  |  | EU797207 | - |  |  |
|  |  | EU797208 | - |  |  |
|  |  | EU797209 | - |  |  |
|  |  | EU797210 | - |  |  |
|  |  | EU797211 | - |  |  |
|  |  | EU797212 | EU812775 |  |  |
|  |  | EU797213 | EU812790 |  |  |
|  |  | EU797214 | - |  |  |
|  |  | EU797215 | - |  |  |
|  |  | EU797216 | - |  |  |
|  |  | EU797217 | EU812782 |  |  |
|  |  | EU797218 | EU812780 |  |  |
|  |  | EU797219 | - |  |  |
|  |  | EU797220 | - |  |  |
|  |  | EU797221 | - |  |  |
|  |  | EU797222 | - |  |  |
|  |  | EU797223 | EU812779 |  |  |
|  |  | EU797223 | EU812788 |  |  |
|  |  | EU797224 | EU812787 |  |  |
|  |  | EU797224 | EU812786 |  |  |
|  |  | EU797225 | EU812785 |  |  |
|  |  | EU797226 | - |  |  |
|  |  | EU797227 | - |  |  |
|  |  | EU797228 | - |  |  |
|  |  | EU797229 | EU812770 |  |  |
|  |  | EU797230 | - |  |  |
|  |  | EU797231 | EU812772 |  |  |
|  |  | EU797231 | EU812784 |  |  |
|  |  | EU797231 | EU812789 |  |  |
|  | *An. saeungae* | EU797232 | - | Thailand |  |
|  |  | EU797233 | - |  |  |
|  |  | EU797234 | - |  |  |
|  |  | EU797235 | - |  |  |
|  |  | EU797236 | - |  |  |
|  |  | EU797237 | - |  |  |
|  |  | EU797238 | - |  |  |
|  |  | EU797239 | EU812800 |  |  |
|  |  | EU797240 | - |  |  |
|  |  | EU797241 | EU812807 |  |  |
|  |  | EU797242 | - |  |  |
|  |  | EU797243 | EU812803 |  |  |
|  |  | EU797244 | - |  |  |
|  |  | EU797245 | EU812791 | Indonesia |  |
|  |  | EU797246 | - | Thailand |  |
|  |  | EU797247 | - |  |  |
|  |  | EU797248 | EU812794 |  |  |
|  |  | EU797249 | - |  |  |
|  |  | EU797250 | - |  |  |
|  |  | EU797251 | - |  |  |
|  |  | EU797252 | - |  |  |
|  |  | EU797253 | - |  |  |
|  |  | EU797254 | EU812798 |  |  |
|  |  | EU797255 | EU812799 |  |  |
|  |  | EU797256 | - |  |  |
|  |  | EU797257 | EU812792 |  |  |
|  |  | EU797258 | EU812801 |  |  |
|  |  | EU797259 | EU812793 |  |  |
|  |  | EU797259 | EU812797 |  |  |
|  |  | EU797260 | EU812802 |  |  |
|  |  | EU797261 | - |  |  |
|  |  | EU797262 | EU812804 |  |  |
|  |  | EU797263 | EU812806 |  |  |
|  |  | EU797264 | - |  |  |
|  |  | EU797265 | - |  |  |
|  |  | EU797266 | - |  |  |
|  |  | EU797267 | - |  |  |
|  |  | EU797268 | EU812805 |  |  |
|  |  | EU797269 | - |  |  |
|  |  | EU797270 | - |  |  |
|  |  | EU797271 | - |  |  |
|  |  | EU797272 | - |  |  |
|  |  | EU797273 | - |  |  |
|  |  | EU797274 | - |  |  |
|  |  | - | EU812795 |  |  |
|  |  | - | EU812796 |  |  |
|  | *An. wejchoochotei* | EU797275 | - | Thailand |  |
|  |  | EU797276 | - |  |  |
|  |  | EU797277 | - |  |  |
|  |  | EU797278 | - |  |  |
|  |  | EU797279 | EU812808 |  |  |
|  |  | EU797280 | - |  |  |
|  |  | - | EU812809 |  |  |
|  | *An. barbirostris* s.l. | - | KJ462237 | Selangor | [2] |
|  |  | - | KJ462238 |  |  |
|  |  | - | KJ462239 |  |  |
|  |  | - | KJ462240 |  |  |
|  |  | - | KJ462241 |  |  |
|  |  | - | KJ462242 |  |  |
|  |  | - | KJ462243 |  |  |
|  |  | - | KJ462244 |  |  |
|  |  | - | KJ462245 |  |  |
|  |  | - | KJ462246 |  |  |
|  |  | - | KJ462247 |  |  |
|  |  | - | KJ462248 |  |  |
|  | *An. donaldi* | MT669935.1 | MT623054.1 | Sabah, Malaysia | Unpublished |
|  |  | MT669936.1 | MT623054.1 |  |  |
|  |  | MT669937.1 | MT623054.1 |  |  |
|  |  | MT669938.1 | MT623054.1 |  |  |
|  |  | MT669939.1 | MT623054.1 |  |  |
|  | LW44 | MN520342 | MN386201 | Sarawak | Present study |
|  | LW47 | MN520344 | MN386197 |  |  |
|  | LW48 | MN520345 | MN386198 |  |  |
|  | LW57 | MN520349 | MN386199 |  |  |
|  | LW58 | MN520350 | MN386200 |  |  |
| Leucosphyrus Subgroup | *An. baimaii* | DQ897952 | - | Thailand | [3] |
|  |  | DQ897953 | - |  |  |
|  |  | DQ897954 | - |  |  |
|  |  | DQ897955 | - | Myanmar |  |
|  | *An. balabacensis* | DQ897940 | - | Sabah, Malaysia |  |
|  |  | DQ897941 | - | South Kalimantan, Indonesia |  |
|  | *An. cracens* | DQ897947 | - | Terengganu, Malaysia |  |
|  |  | DQ897948 | - |  |  |
|  | *An. elegans* | DQ897957 | - | India |  |
|  |  | DQ897958 | - |  |  |
|  | *An. macarthuri* | DQ897969 | - | Thailand |  |
|  |  | DQ897970 | - |  |  |
|  |  | DQ897971 | - |  |  |
|  |  | DQ897972 | - |  |  |
|  | *An. mirans* | DQ897965 | - | Sri Lanka |  |
|  |  | DQ897966 | - | India |  |
|  | *An. scanloni* | DQ897949 | - | Thailand |  |
|  | *An. nemophilous* | DQ897961 | - |  |  |
|  | *An. takasagoensis* | DQ897962 | - | China |  |
|  |  | DQ897963 | - |  |  |
|  |  | DQ897964 | - |  |  |
|  | *An. dirus* | DQ897945 | - | Thailand |  |
|  |  | DQ897946 | - |  |  |
|  |  | AB518500 | - | Vietnam | [4] |
|  |  | AB518501 | - |  |  |
|  |  | AB518502 | - |  |  |
|  |  | AB518503 | - |  |  |
|  |  | AB518504 | - |  |  |
|  |  | AB518505 | - |  |  |
|  |  | AB518506 | - |  |  |
|  |  | AB518507 | - |  |  |
|  |  | AB518508 | - |  |  |
|  |  | AB518509 | - |  |  |
|  |  | AB518510 | - |  |  |
|  |  | AB518511 | - |  |  |
|  |  | AB518512 | - |  |  |
|  |  | AB518513 | - |  |  |
|  |  | AB518514 | - |  |  |
|  |  | AB518515 | - |  |  |
|  |  | AB518516 | - |  |  |
|  |  | AB518517 | - |  |  |
|  |  | AB518518 | - |  |  |
|  |  | AB518519 | - |  |  |
|  |  | AB518520 | - |  |  |
|  |  | AB518521 | - |  |  |
|  |  | AB518522 | - |  |  |
|  |  | AB518523 | - |  |  |
|  |  | AB518524 | - |  |  |
|  |  | AB518525 | - |  |  |
|  |  | AB518526 | - |  |  |
|  |  | AB518527 | - |  |  |
|  |  | AB518528 | - |  |  |
|  |  | AB518529 | - |  |  |
|  |  | AB518530 | - |  |  |
|  |  | AB518531 | - |  |  |
|  |  | AB518532 | - |  |  |
|  |  | AB518533 | - |  |  |
|  | *An. takasagoensis* cf | AB518534 | - |  |  |
|  |  | AB518535 | - |  |  |
|  |  | AB518536 | - |  |  |
|  |  | AB518537 | - |  |  |
|  |  | AB518538 | - |  |  |
|  | *An. introlatus* | KM032610 | - | Selangor, Malaysia | Unpublished |
|  |  | KM032611 | - |  |  |
|  |  | KM032612 | - |  |  |
|  | LW31 | MN520340 | - | Sarawak | Present study |
|  | LW32 | MN520341 | - |  |  |
|  | LW45 | MN520343 | - |  |  |
|  | LW49 | MN520346 | - |  |  |
|  | LW50 | MN520347 | - |  |  |
|  | LW51 | MN520348 | - |  |  |
|  | LW59 | MN520351 | - |  |  |
|  | LW67 | MN520352 | - |  |  |
|  | LW74 | MN520353 | - |  |  |
| Outgroups | *An. coustani* | AF417715 | - | Kenya | [5] |
|  | *An. gambiae* | L20934 | - | Africa | [6] |

**References**

1. Paredes-Esquivel C, Donnelly MJ, Harbach RE, Townson H. A molecular phylogeny of mosquitoes in the *Anopheles barbirostris* Subgroup reveals cryptic species: Implications for identification of disease vectors. Mol Phylogenet Evol. Elsevier Inc.; 2009;50:141–51.

2. Sum J-S, Lee W-C, Amir A, Braima K a, Jeffery J, Abdul-Aziz NM, et al. Phylogenetic study of six species of *Anopheles* mosquitoes in Peninsular Malaysia based on inter-transcribed spacer region 2 (ITS2) of ribosomal DNA. Parasit Vectors. 2014;7:309.

3. Sallum MAM, Foster PG, Li C, Sithiprasasna R, Wilkerson RC. Phylogeny of the *Leucosphyrus* Group of *Anopheles* (Cellia) (Diptera: Culcidae) based on mitochondrial gene sequences. Ann Entomol Soc Am. 2007;100:27–35.

4. Takano KT, Nguyen NTH, Nguyen BTH, Sunahara T, Yasunami M, Nguyen MD, et al. Partial mitochondrial DNA sequences suggest the existence of a cryptic species within the *Leucosphyrus* group of the genus *Anopheles* (Diptera: Culicidae), forest malaria vectors, in northern Vietnam. Parasites and Vectors. 2010;3.

5. Sallum MAM, Schultz TR, Foster PG, Aronstein K, Wirtz RA, Wilkerson RC. Phylogeny of Anophelinae (Diptera: Culicidae) based on nuclear ribosomal and mitochondrial DNA sequences. Syst Entomol. 2002;27:361–82.

6. Beard CB, Hamm DM, Collins FH. The mitochondrial genome of the mosquito *Anopheles gambiae*: DNA sequence, genome organization, and comparisons with mitochondrial sequences of other insects. Insect Mol Biol. 1993;2:103–24.
