# Supplementary material for: New vectors in northern Sarawak, Malaysian Borneo, for the zoonotic malaria parasite, Plasmodium knowlesi
Source: Parasit Vectors. 2020 Sep 15;13:472. doi: 10.1186/s13071-020-04345-2 (PMC7490903; doi:10.1186/s13071-020-04345-2)
Supplement: Supplementary file 4 — Additional file 4: Alignment S2. Multiple sequence alignment of the cox1 gene of An. leucosphyrus (s.l.) and an outgroup, An. gambiae. [file 13071_2020_4345_MOESM4_ESM.docx]

| Species* | Accession/  Sample  ID | Position of SNP (bp)** | | | | | | | | | | | | | | | | | | | | | | | | | | | | | | | | | | | | | | | | | | | | | | | | | | | | | | | | | | | |
| --- | --- | --- | --- | --- | --- | --- | --- | --- | --- | --- | --- | --- | --- | --- | --- | --- | --- | --- | --- | --- | --- | --- | --- | --- | --- | --- | --- | --- | --- | --- | --- | --- | --- | --- | --- | --- | --- | --- | --- | --- | --- | --- | --- | --- | --- | --- | --- | --- | --- | --- | --- | --- | --- | --- | --- | --- | --- | --- | --- | --- | --- |
|  |  |  |  |  |  |  |  |  |  |  | 1 | 1 | 1 | 1 | 1 | 1 | 1 | 1 | 1 | 1 | 2 | 2 | 2 | 2 | 2 | 2 | 2 | 2 | 2 | 2 | 3 | 3 | 3 | 3 | 3 | 3 | 3 | 3 | 3 | 3 | 4 | 4 | 4 | 4 | 4 | 4 | 4 | 4 | 4 | 4 | 5 | 5 | 5 | 5 | 5 | 5 | 5 | 5 | 5 | 5 | 6 |
|  |  | 1 | 2 | 3 | 4 | 5 | 6 | 7 | 8 | 9 | 0 | 1 | 2 | 3 | 4 | 5 | 6 | 7 | 8 | 9 | 0 | 1 | 2 | 3 | 4 | 5 | 6 | 7 | 8 | 9 | 0 | 1 | 2 | 3 | 4 | 5 | 6 | 7 | 8 | 9 | 0 | 1 | 2 | 3 | 4 | 5 | 6 | 7 | 8 | 9 | 0 | 1 | 2 | 3 | 4 | 5 | 6 | 7 | 8 | 9 | 0 |
| Bal | DQ897940 | A | T | T | A | G | C | A | G | G | A | A | T | A | C | C | T | C | G | A | C | G | A | T | A | C | T | C | G | G | A | T | T | T | T | C | C | T | G | A | T | A | G | T | T | A | C | T | T | A | A | C | A | T | G | A | A | A | T | A | T |
|  | DQ897941 | ● | ● | ● | ● | ● | ● | ● | ● | ● | ● | ● | ● | ● | ● | ● | ● | ● | ● | ● | ● | ● | ● | ● | ● | ● | ● | ● | ● | ● | ● | ● | ● | ● | ● | ● | ● | ● | ● | ● | ● | ● | ● | ● | ● | ● | ● | ● | ● | ● | ● | ● | ● | ● | ● | ● | ● | ● | ● | ● | ● |
|  | LW31 | ● | ● | ● | ● | ● | ● | ● | ● | ● | ● | ● | ● | ● | ● | ● | ● | ● | ● | ● | ● | ● | ● | ● | ● | ● | ● | ● | ● | ● | ● | ● | ● | ● | ● | ● | ● | ● | ● | ● | ● | ● | ● | ● | ● | ● | ● | ● | ● | ● | ● | ● | ● | ● | ● | ● | ● | ● | ● | ● | ● |
|  | LW32 | ● | ● | ● | ● | ● | ● | ● | ● | ● | ● | ● | ● | ● | ● | ● | ● | ● | ● | ● | ● | ● | ● | ● | ● | ● | ● | ● | ● | ● | ● | ● | ● | ● | ● | ● | ● | ● | ● | ● | ● | ● | ● | ● | ● | ● | ● | ● | ● | ● | ● | ● | ● | ● | ● | ● | ● | ● | ● | ● | ● |
|  | LW45 | ● | ● | ● | ● | ● | ● | ● | ● | ● | ● | ● | ● | ● | ● | ● | ● | ● | ● | ● | ● | ● | ● | ● | ● | ● | ● | ● | ● | ● | ● | ● | ● | ● | ● | ● | ● | ● | ● | ● | ● | ● | ● | ● | ● | ● | ● | ● | ● | ● | ● | ● | ● | ● | ● | ● | ● | ● | ● | ● | ● |
|  | LW49 | ● | ● | ● | ● | ● | ● | ● | ● | ● | ● | ● | ● | ● | ● | ● | ● | ● | ● | ● | ● | ● | ● | ● | ● | ● | ● | ● | ● | ● | ● | ● | ● | ● | ● | ● | ● | ● | ● | ● | ● | ● | ● | ● | ● | ● | ● | ● | ● | ● | ● | ● | ● | ● | ● | ● | ● | ● | ● | ● | ● |
|  | LW50 | ● | ● | ● | ● | ● | ● | ● | ● | ● | ● | ● | ● | ● | ● | ● | ● | ● | ● | ● | ● | ● | ● | ● | ● | ● | ● | ● | ● | ● | ● | ● | ● | ● | ● | ● | ● | ● | ● | ● | ● | ● | ● | ● | ● | ● | ● | ● | ● | ● | ● | ● | ● | ● | ● | ● | ● | ● | ● | ● | ● |
|  | LW51 | ● | ● | ● | ● | ● | ● | ● | ● | ● | ● | ● | ● | ● | ● | ● | ● | ● | ● | ● | ● | ● | ● | ● | ● | ● | ● | ● | ● | ● | ● | ● | ● | ● | ● | ● | ● | ● | ● | ● | ● | ● | ● | ● | ● | ● | ● | ● | ● | ● | ● | ● | ● | ● | ● | ● | ● | ● | ● | ● | ● |
|  | LW59 | ● | ● | ● | ● | ● | ● | ● | ● | ● | ● | ● | ● | ● | ● | ● | ● | ● | ● | ● | ● | ● | ● | ● | ● | ● | ● | ● | ● | ● | ● | ● | ● | ● | ● | ● | ● | ● | ● | ● | ● | ● | ● | ● | ● | ● | ● | ● | ● | ● | ● | ● | ● | ● | ● | ● | ● | ● | ● | ● | ● |
|  | LW67 | ● | ● | ● | ● | ● | ● | ● | ● | ● | ● | ● | ● | ● | ● | ● | ● | ● | ● | ● | ● | ● | ● | ● | ● | ● | ● | ● | ● | ● | ● | ● | ● | ● | ● | ● | ● | ● | ● | ● | ● | ● | ● | ● | ● | ● | ● | ● | ● | ● | ● | ● | ● | ● | ● | ● | ● | ● | ● | ● | ● |
|  | LW74 | ● | ● | ● | ● | ● | ● | ● | ● | ● | ● | ● | ● | ● | ● | ● | ● | ● | ● | ● | ● | ● | ● | ● | ● | ● | ● | ● | ● | ● | ● | ● | ● | ● | ● | ● | ● | ● | ● | ● | ● | ● | ● | ● | ● | ● | ● | ● | ● | ● | ● | ● | ● | ● | ● | ● | ● | ● | ● | ● | ● |
| Bai | DQ897952 | ● | ● | ● | ● | ● | ● | C | ● | ● | G | ● | ● | ● | ● | ● | ● | ● | ● | ● | ● | ● | ● | ● | ● | ● | ● | ● | A | ● | ● | ● | ● | ● | ● | ● | ● | ● | ● | ● | ● | ● | ● | C | ● | ● | ● | ● | ● | ● | ● | ● | ● | ● | ● | ● | ● | ● | ● | ● | ● |
|  | DQ897953 | ● | ● | ● | ● | ● | ● | C | ● | ● | G | ● | ● | ● | ● | ● | ● | ● | ● | ● | ● | ● | ● | ● | ● | ● | ● | ● | A | ● | ● | ● | ● | ● | ● | ● | ● | ● | ● | ● | ● | ● | ● | C | ● | ● | ● | ● | ● | ● | ● | ● | ● | ● | ● | ● | ● | ● | ● | ● | ● |
|  | DQ897954 | ● | ● | ● | ● | ● | ● | C | ● | ● | G | ● | ● | ● | ● | ● | ● | ● | ● | ● | ● | ● | ● | ● | ● | ● | ● | ● | A | ● | ● | ● | ● | ● | ● | ● | ● | ● | ● | ● | ● | ● | ● | C | ● | ● | ● | ● | ● | ● | ● | ● | ● | ● | ● | ● | ● | ● | ● | ● | ● |
|  | DQ897955 | ● | ● | ● | ● | ● | ● | C | ● | ● | G | ● | ● | ● | ● | ● | ● | ● | ● | ● | ● | ● | ● | ● | ● | ● | ● | ● | A | ● | ● | ● | ● | ● | ● | ● | ● | ● | ● | ● | ● | ● | ● | C | ● | ● | T | ● | ● | ● | ● | ● | ● | ● | ● | ● | ● | ● | ● | ● | ● |
| Cra | DQ897947 | ● | ● | ● | ● | ● | ● | T | ● | ● | ● | ● | ● | ● | ● | ● | ● | ● | ● | ● | ● | ● | ● | ● | ● | ● | ● | ● | A | ● | ● | ● | ● | ● | ● | ● | ● | ● | ● | ● | ● | ● | ● | ● | ● | ● | ● | ● | ● | ● | ● | ● | ● | ● | ● | ● | ● | ● | ● | ● | ● |
|  | DQ897948 | ● | ● | ● | ● | ● | ● | T | ● | ● | ● | ● | ● | ● | ● | ● | ● | ● | ● | ● | ● | ● | ● | ● | ● | ● | ● | ● | A | ● | ● | ● | ● | ● | ● | ● | ● | ● | ● | ● | ● | ● | ● | ● | ● | ● | ● | ● | ● | ● | ● | ● | ● | ● | ● | ● | ● | ● | ● | ● | ● |
| Dir | AB518499 | ● | ● | ● | ● | ● | ● | C | ● | ● | ● | ● | ● | ● | ● | ● | ● | ● | ● | ● | ● | ● | ● | ● | ● | ● | ● | ● | A | ● | ● | ● | ● | ● | ● | ● | ● | ● | ● | ● | ● | ● | ● | C | ● | ● | ● | ● | ● | ● | ● | ● | ● | ● | ● | ● | ● | ● | ● | ● | ● |
|  | AB518500 | ● | ● | ● | ● | ● | ● | C | ● | ● | G | ● | ● | ● | ● | ● | ● | ● | ● | ● | ● | ● | ● | ● | ● | ● | ● | ● | A | ● | ● | ● | ● | ● | ● | ● | ● | ● | ● | ● | ● | ● | ● | C | ● | ● | ● | ● | ● | ● | ● | ● | ● | ● | ● | ● | ● | ● | ● | ● | ● |
|  | AB518501 | ● | ● | ● | ● | ● | ● | ● | ● | ● | G | ● | ● | ● | ● | ● | ● | ● | ● | ● | ● | ● | ● | ● | ● | ● | ● | ● | A | ● | ● | ● | ● | ● | ● | ● | ● | ● | ● | ● | ● | ● | ● | C | ● | ● | ● | ● | ● | ● | ● | ● | ● | ● | ● | ● | ● | ● | ● | ● | ● |
|  | AB518502 | ● | ● | ● | ● | ● | ● | ● | ● | ● | G | ● | ● | ● | ● | ● | ● | ● | ● | ● | ● | ● | ● | ● | ● | ● | ● | ● | A | ● | ● | ● | ● | ● | ● | ● | ● | ● | ● | ● | ● | ● | ● | C | ● | ● | ● | ● | ● | ● | ● | ● | ● | ● | ● | ● | ● | ● | ● | ● | ● |
|  | AB518503 | ● | ● | ● | ● | ● | ● | C | ● | ● | ● | ● | ● | ● | ● | ● | ● | ● | ● | ● | ● | ● | ● | ● | ● | ● | ● | ● | A | ● | ● | ● | ● | ● | ● | ● | ● | ● | ● | ● | ● | ● | ● | C | ● | ● | ● | ● | ● | ● | ● | ● | ● | ● | ● | ● | ● | ● | ● | ● | ● |
|  | AB518504 | ● | ● | ● | ● | ● | ● | C | ● | ● | ● | ● | ● | ● | ● | ● | ● | ● | ● | ● | ● | ● | ● | ● | ● | ● | ● | ● | A | ● | ● | ● | ● | ● | ● | ● | ● | ● | ● | ● | ● | ● | ● | C | ● | ● | ● | ● | ● | ● | ● | ● | ● | ● | ● | ● | ● | ● | ● | ● | ● |
|  | AB518505 | ● | ● | ● | ● | ● | ● | C | ● | ● | ● | ● | ● | ● | ● | ● | ● | ● | ● | ● | ● | ● | ● | ● | ● | ● | ● | ● | A | ● | ● | ● | ● | ● | ● | ● | ● | ● | ● | ● | ● | ● | ● | C | ● | ● | ● | ● | ● | ● | ● | ● | ● | ● | ● | ● | ● | ● | ● | ● | ● |
|  | AB518506 | ● | ● | ● | ● | ● | ● | C | ● | ● | G | ● | ● | ● | ● | ● | ● | ● | ● | ● | ● | ● | ● | ● | ● | ● | ● | ● | A | ● | ● | ● | ● | ● | ● | ● | ● | ● | ● | ● | ● | ● | ● | C | ● | ● | ● | ● | ● | ● | ● | ● | ● | ● | ● | ● | ● | ● | ● | ● | ● |
|  | AB518507 | ● | ● | ● | ● | ● | ● | C | ● | ● | G | ● | ● | ● | ● | ● | ● | ● | ● | ● | ● | ● | ● | ● | ● | ● | ● | ● | A | ● | ● | ● | ● | ● | ● | ● | ● | ● | ● | ● | ● | ● | ● | C | ● | ● | ● | ● | ● | ● | ● | ● | ● | ● | ● | ● | ● | ● | ● | ● | ● |
|  | AB518508 | ● | ● | ● | ● | ● | ● | C | ● | ● | G | ● | ● | ● | ● | ● | ● | ● | ● | ● | ● | ● | ● | ● | ● | ● | ● | ● | A | ● | ● | ● | ● | ● | ● | ● | ● | ● | ● | ● | ● | ● | ● | C | ● | ● | ● | ● | ● | ● | ● | ● | ● | ● | ● | ● | ● | ● | ● | ● | ● |
|  | AB518509 | ● | ● | ● | ● | ● | ● | C | ● | ● | G | ● | ● | ● | ● | ● | ● | ● | ● | ● | ● | ● | ● | ● | ● | ● | ● | ● | A | ● | ● | ● | ● | ● | ● | ● | ● | ● | ● | ● | ● | ● | ● | C | ● | ● | ● | ● | ● | ● | ● | ● | ● | ● | ● | ● | ● | ● | ● | ● | ● |
|  | AB518510 | ● | ● | ● | ● | ● | ● | C | ● | ● | G | ● | ● | ● | ● | ● | ● | ● | ● | ● | ● | ● | ● | ● | ● | ● | ● | ● | A | ● | ● | ● | ● | ● | ● | ● | ● | ● | ● | ● | ● | ● | ● | C | ● | ● | ● | ● | ● | ● | ● | ● | ● | ● | ● | ● | ● | ● | ● | ● | ● |
|  | AB518511 | ● | ● | ● | ● | ● | ● | C | ● | ● | G | ● | ● | ● | ● | ● | ● | ● | ● | ● | ● | ● | ● | ● | ● | ● | ● | ● | A | ● | ● | ● | ● | ● | ● | ● | ● | ● | ● | ● | ● | ● | ● | C | ● | ● | ● | ● | ● | ● | ● | ● | ● | ● | ● | ● | ● | ● | ● | ● | ● |
|  | AB518512 | ● | ● | ● | ● | ● | ● | C | ● | ● | ● | ● | ● | ● | ● | ● | ● | ● | ● | ● | ● | ● | ● | ● | ● | ● | ● | ● | A | ● | ● | ● | ● | ● | ● | ● | ● | ● | ● | ● | ● | ● | ● | C | ● | ● | ● | ● | ● | ● | ● | ● | ● | ● | ● | ● | ● | ● | ● | ● | ● |
|  | AB518513 | ● | ● | ● | ● | ● | ● | ● | ● | ● | G | ● | ● | ● | ● | ● | ● | ● | ● | ● | ● | ● | ● | ● | ● | ● | ● | ● | A | ● | ● | ● | ● | ● | ● | ● | ● | ● | ● | ● | ● | ● | ● | C | ● | ● | ● | ● | ● | ● | ● | ● | ● | ● | ● | ● | ● | ● | ● | ● | ● |
|  | AB518514 | ● | ● | ● | ● | ● | ● | ● | ● | ● | G | ● | ● | ● | ● | ● | ● | ● | ● | ● | ● | ● | ● | ● | ● | ● | ● | ● | A | ● | ● | ● | ● | ● | ● | ● | ● | ● | ● | ● | ● | ● | ● | C | ● | ● | ● | ● | ● | ● | ● | ● | ● | ● | ● | ● | ● | ● | ● | ● | ● |
|  | AB518515 | ● | ● | ● | ● | ● | ● | ● | ● | ● | G | ● | ● | ● | ● | ● | ● | ● | ● | ● | ● | ● | ● | ● | ● | ● | ● | ● | A | ● | ● | ● | ● | ● | ● | ● | ● | ● | ● | ● | ● | ● | ● | C | ● | ● | ● | ● | ● | ● | ● | ● | ● | ● | ● | ● | ● | ● | ● | ● | ● |
|  | AB518516 | ● | ● | ● | ● | ● | ● | ● | ● | ● | G | ● | ● | ● | ● | ● | ● | ● | ● | ● | ● | ● | ● | ● | ● | ● | ● | ● | A | ● | ● | ● | ● | ● | ● | ● | ● | ● | ● | ● | ● | ● | ● | C | ● | ● | ● | ● | ● | ● | ● | ● | ● | ● | ● | ● | ● | ● | ● | ● | ● |
|  | AB518517 | ● | ● | ● | ● | ● | ● | C | ● | ● | G | ● | ● | ● | ● | ● | ● | ● | ● | ● | ● | ● | ● | ● | ● | ● | ● | ● | A | ● | ● | ● | ● | ● | ● | ● | ● | ● | ● | ● | ● | ● | ● | C | ● | ● | ● | ● | ● | ● | ● | ● | ● | ● | ● | ● | ● | ● | ● | ● | ● |
|  | AB518518 | ● | ● | ● | ● | ● | ● | C | ● | ● | G | ● | ● | ● | ● | ● | ● | ● | ● | ● | ● | ● | ● | ● | ● | ● | ● | ● | A | ● | ● | ● | ● | ● | ● | ● | ● | ● | ● | ● | ● | ● | ● | C | ● | ● | ● | ● | ● | ● | ● | ● | ● | ● | ● | ● | ● | ● | ● | ● | ● |
|  | AB518519 | ● | ● | ● | ● | ● | ● | C | ● | ● | G | ● | ● | ● | ● | ● | ● | ● | ● | ● | ● | ● | ● | ● | ● | ● | ● | ● | A | ● | ● | ● | ● | ● | ● | ● | ● | ● | ● | ● | ● | ● | ● | C | ● | ● | ● | ● | ● | ● | ● | ● | ● | ● | ● | ● | ● | ● | ● | ● | ● |
|  | AB518520 | ● | ● | ● | ● | ● | ● | C | ● | ● | ● | ● | ● | ● | ● | ● | ● | ● | ● | ● | ● | ● | ● | ● | ● | ● | ● | ● | A | ● | ● | ● | ● | ● | ● | ● | ● | ● | ● | ● | ● | ● | ● | C | ● | ● | ● | ● | ● | ● | ● | ● | ● | ● | ● | ● | ● | ● | ● | ● | ● |
|  | AB518521 | ● | ● | ● | ● | ● | ● | C | ● | ● | G | ● | ● | ● | ● | ● | ● | ● | ● | ● | ● | ● | ● | ● | ● | ● | ● | ● | A | ● | ● | ● | ● | ● | ● | ● | ● | ● | ● | ● | ● | ● | ● | C | ● | ● | ● | ● | ● | ● | ● | ● | ● | ● | ● | ● | ● | ● | ● | ● | ● |
|  | AB518522 | ● | ● | ● | ● | ● | ● | C | ● | ● | G | ● | ● | ● | ● | ● | ● | ● | ● | ● | ● | ● | ● | ● | ● | ● | ● | ● | A | ● | ● | ● | ● | ● | ● | ● | ● | ● | ● | ● | ● | ● | ● | C | ● | ● | ● | ● | ● | ● | ● | ● | ● | ● | ● | ● | ● | ● | ● | ● | ● |
|  | AB518523 | ● | ● | ● | ● | ● | ● | C | ● | ● | G | ● | ● | ● | ● | ● | ● | ● | ● | ● | ● | ● | ● | ● | ● | ● | ● | ● | A | ● | ● | ● | ● | ● | ● | ● | ● | ● | ● | ● | ● | ● | ● | C | ● | ● | ● | ● | ● | ● | ● | ● | ● | ● | ● | ● | ● | ● | ● | ● | ● |
|  | AB518524 | ● | ● | ● | ● | ● | ● | C | ● | ● | G | ● | ● | ● | ● | ● | ● | ● | ● | ● | ● | ● | ● | ● | ● | ● | ● | ● | A | ● | ● | ● | ● | ● | ● | ● | ● | ● | ● | ● | ● | ● | ● | C | ● | ● | ● | ● | ● | ● | ● | ● | ● | ● | ● | ● | ● | ● | ● | ● | ● |
|  | AB518525 | ● | ● | ● | ● | ● | ● | C | ● | ● | G | ● | ● | ● | ● | ● | ● | ● | ● | ● | ● | ● | ● | ● | ● | ● | ● | ● | A | ● | ● | ● | ● | ● | ● | ● | ● | ● | ● | ● | ● | ● | ● | C | ● | ● | ● | ● | ● | ● | ● | ● | ● | ● | ● | ● | ● | ● | ● | ● | ● |
|  | AB518526 | ● | ● | ● | ● | ● | ● | C | ● | ● | ● | ● | ● | ● | ● | ● | ● | ● | ● | ● | ● | ● | ● | ● | ● | ● | ● | ● | A | ● | ● | ● | ● | ● | ● | ● | ● | ● | ● | ● | ● | ● | ● | C | ● | ● | ● | ● | ● | ● | ● | ● | ● | ● | ● | ● | ● | ● | ● | ● | ● |
|  | AB518527 | ● | ● | ● | ● | ● | ● | C | ● | ● | G | ● | ● | ● | ● | ● | ● | ● | ● | ● | ● | ● | ● | ● | ● | ● | ● | ● | A | ● | ● | ● | ● | ● | ● | ● | ● | ● | ● | ● | ● | ● | ● | C | ● | ● | ● | ● | ● | ● | ● | ● | ● | ● | ● | ● | ● | ● | ● | ● | ● |
|  | AB518528 | ● | ● | ● | ● | ● | ● | ● | ● | ● | G | ● | ● | ● | ● | ● | ● | ● | ● | ● | ● | ● | ● | ● | ● | ● | ● | ● | A | ● | ● | ● | ● | ● | ● | ● | ● | ● | ● | ● | ● | ● | ● | C | ● | ● | ● | ● | ● | ● | ● | ● | ● | ● | ● | ● | ● | ● | ● | ● | ● |
|  | AB518529 | ● | ● | ● | ● | ● | ● | C | ● | ● | G | ● | ● | ● | ● | ● | ● | ● | ● | ● | ● | ● | ● | ● | ● | ● | ● | ● | A | ● | ● | ● | ● | ● | ● | ● | ● | ● | ● | ● | ● | ● | ● | C | ● | ● | ● | ● | ● | ● | ● | ● | ● | ● | ● | ● | ● | ● | ● | ● | ● |
|  | AB518530 | ● | ● | ● | ● | ● | ● | C | ● | ● | G | ● | ● | ● | ● | ● | ● | ● | ● | ● | ● | ● | ● | ● | ● | ● | ● | ● | A | ● | ● | ● | ● | ● | ● | ● | ● | ● | ● | ● | ● | ● | ● | A | ● | ● | ● | ● | ● | ● | ● | ● | ● | ● | ● | ● | ● | ● | ● | ● | ● |
|  | AB518531 | ● | ● | ● | ● | ● | ● | ● | ● | ● | ● | ● | ● | ● | ● | ● | ● | ● | ● | ● | ● | ● | ● | ● | ● | ● | ● | ● | A | ● | ● | ● | ● | ● | ● | ● | ● | ● | ● | ● | ● | ● | ● | C | ● | ● | ● | ● | ● | ● | ● | ● | ● | ● | ● | ● | ● | ● | ● | ● | ● |
|  | AB518532 | ● | ● | ● | ● | ● | ● | ● | ● | ● | ● | ● | ● | ● | ● | ● | ● | ● | ● | ● | ● | ● | ● | ● | ● | ● | ● | ● | A | ● | ● | ● | ● | ● | ● | ● | ● | ● | ● | ● | ● | ● | ● | C | ● | ● | ● | ● | ● | ● | ● | ● | ● | ● | ● | ● | ● | ● | ● | ● | ● |
|  | AB518533 | G | ● | ● | ● | ● | ● | C | ● | ● | G | ● | ● | ● | ● | ● | ● | ● | ● | ● | ● | ● | ● | ● | ● | ● | ● | ● | A | ● | ● | ● | ● | ● | ● | ● | ● | ● | ● | ● | ● | ● | ● | C | ● | ● | ● | ● | ● | ● | ● | ● | ● | ● | ● | ● | ● | ● | ● | ● | ● |
|  | DQ897944 | ● | ● | ● | ● | ● | ● | C | ● | ● | G | ● | ● | ● | ● | ● | ● | ● | ● | ● | ● | ● | ● | ● | ● | ● | ● | ● | A | ● | ● | ● | ● | ● | ● | ● | ● | ● | ● | ● | ● | ● | ● | C | ● | ● | ● | ● | ● | ● | ● | ● | ● | ● | ● | ● | ● | ● | ● | ● | ● |
|  | DQ897945 | ● | ● | ● | ● | ● | ● | C | ● | ● | G | ● | ● | ● | ● | ● | ● | ● | ● | ● | ● | ● | ● | ● | ● | ● | ● | ● | A | ● | ● | ● | ● | ● | ● | ● | ● | ● | ● | ● | ● | ● | ● | C | ● | ● | ● | ● | ● | ● | ● | ● | ● | ● | ● | ● | ● | ● | ● | ● | ● |
|  | DQ897946 | ● | ● | ● | ● | ● | ● | C | ● | ● | G | ● | ● | ● | ● | ● | ● | ● | ● | ● | ● | ● | ● | ● | ● | ● | ● | ● | A | ● | ● | ● | ● | ● | ● | ● | ● | ● | ● | ● | ● | ● | ● | C | ● | ● | ● | ● | ● | ● | ● | ● | ● | ● | ● | ● | ● | ● | ● | ● | ● |
| Ele | DQ897957 | ● | ● | ● | ● | ● | ● | C | ● | ● | G | ● | ● | ● | ● | ● | ● | ● | ● | ● | ● | ● | ● | ● | ● | T | ● | ● | A | ● | ● | ● | ● | ● | ● | ● | ● | ● | ● | ● | C | ● | ● | C | ● | ● | ● | ● | ● | ● | ● | ● | ● | ● | ● | ● | ● | ● | C | ● | ● |
|  | DQ897958 | ● | ● | ● | ● | ● | ● | C | ● | ● | G | ● | ● | ● | ● | ● | ● | ● | ● | ● | ● | ● | ● | ● | ● | T | ● | ● | A | ● | ● | ● | ● | ● | ● | ● | ● | ● | ● | ● | C | ● | ● | C | ● | ● | ● | ● | ● | ● | ● | ● | ● | ● | ● | ● | ● | ● | C | ● | ● |
| Gam | L20934 | T | ● | ● | ● | ● | ● | C | ● | ● | ● | ● | ● | ● | ● | ● | ● | ● | ● | ● | ● | ● | ● | ● | ● | T | ● | ● | A | ● | ● | C | ● | ● | ● | ● | ● | A | ● | ● | ● | ● | ● | C | ● | ● | T | ● | ● | ● | ● | ● | ● | ● | ● | ● | ● | ● | ● | G | ● |
| Int | KM032605 | ● | ● | ● | G | ● | ● | T | ● | ● | ● | ● | ● | ● | ● | ● | A | ● | ● | ● | ● | ● | ● | ● | ● | T | ● | ● | A | ● | ● | ● | ● | ● | ● | ● | ● | ● | ● | ● | ● | ● | ● | ● | ● | ● | T | ● | ● | ● | ● | ● | ● | ● | ● | ● | ● | ● | ● | ● | ● |
|  | KM032606 | ● | ● | ● | G | ● | ● | T | ● | ● | ● | ● | ● | ● | ● | ● | A | ● | ● | ● | ● | ● | ● | ● | ● | T | ● | ● | A | ● | ● | ● | ● | ● | ● | ● | ● | ● | ● | ● | ● | ● | ● | ● | ● | ● | T | ● | ● | ● | ● | ● | ● | ● | ● | ● | ● | ● | ● | ● | ● |
|  | KM032607 | ● | ● | ● | G | ● | ● | T | ● | ● | ● | ● | ● | ● | ● | ● | A | ● | ● | ● | ● | ● | ● | ● | ● | T | ● | ● | A | ● | ● | ● | ● | ● | ● | ● | ● | ● | ● | ● | ● | ● | ● | ● | ● | ● | T | ● | ● | ● | ● | ● | ● | ● | ● | ● | ● | ● | ● | ● | ● |
|  | KM032608 | ● | ● | ● | G | ● | ● | T | ● | ● | ● | ● | ● | ● | ● | ● | A | ● | ● | ● | ● | ● | ● | ● | ● | T | ● | ● | A | ● | ● | ● | ● | ● | ● | ● | ● | ● | ● | ● | ● | ● | ● | ● | ● | ● | T | ● | ● | ● | ● | ● | ● | ● | ● | ● | ● | ● | ● | ● | ● |
|  | KM032609 | ● | ● | ● | G | ● | ● | T | ● | ● | ● | ● | ● | ● | ● | ● | A | ● | ● | ● | ● | ● | ● | ● | ● | T | ● | ● | A | ● | ● | ● | ● | ● | ● | ● | ● | ● | ● | ● | ● | ● | ● | ● | ● | ● | T | ● | ● | ● | ● | ● | ● | ● | ● | ● | ● | ● | ● | ● | ● |
|  | KM032610 | ● | ● | ● | ● | ● | ● | T | ● | ● | ● | ● | ● | ● | ● | ● | A | ● | ● | ● | ● | ● | ● | ● | ● | T | ● | ● | A | ● | ● | ● | ● | ● | ● | ● | ● | ● | ● | ● | ● | ● | ● | ● | ● | ● | T | ● | ● | ● | ● | ● | ● | ● | ● | ● | ● | ● | ● | ● | ● |
|  | KM032611 | ● | ● | ● | G | ● | ● | T | ● | ● | ● | ● | ● | ● | ● | ● | A | ● | ● | ● | ● | ● | ● | ● | ● | T | ● | ● | A | ● | ● | ● | ● | ● | ● | ● | ● | ● | ● | ● | ● | ● | ● | ● | ● | ● | T | ● | ● | ● | ● | ● | ● | ● | ● | ● | ● | ● | ● | ● | ● |
|  | KM032612 | ● | ● | ● | ● | ● | ● | T | ● | ● | ● | ● | ● | ● | ● | ● | A | ● | ● | ● | ● | ● | ● | ● | ● | T | ● | ● | A | ● | ● | ● | ● | ● | ● | ● | ● | ● | ● | ● | ● | ● | ● | ● | ● | ● | T | ● | ● | ● | ● | ● | ● | ● | ● | ● | ● | ● | ● | ● | ● |
| Lat | DQ897936 | ● | ● | ● | ● | ● | ● | C | ● | ● | ● | ● | ● | ● | ● | ● | ● | ● | ● | ● | ● | ● | ● | ● | ● | ● | ● | ● | A | ● | ● | ● | ● | ● | C | ● | ● | ● | ● | ● | ● | ● | ● | ● | ● | ● | T | ● | ● | ● | ● | ● | ● | ● | ● | ● | ● | ● | ● | ● | ● |
|  | DQ897937 | ● | ● | ● | ● | ● | ● | C | ● | ● | ● | ● | ● | ● | ● | ● | ● | ● | ● | ● | ● | ● | G | ● | ● | ● | ● | ● | A | ● | ● | ● | ● | ● | C | ● | ● | ● | ● | ● | ● | ● | ● | ● | ● | ● | T | ● | ● | ● | ● | ● | ● | ● | ● | ● | ● | ● | ● | ● | ● |
| Leu | DQ897939 | ● | ● | ● | ● | ● | ● | C | ● | ● | T | ● | ● | ● | ● | ● | ● | ● | ● | ● | ● | ● | ● | ● | ● | ● | ● | ● | ● | ● | ● | ● | ● | ● | C | ● | ● | ● | ● | ● | ● | ● | ● | ● | ● | ● | T | ● | ● | ● | ● | ● | ● | ● | ● | ● | ● | ● | ● | ● | ● |
| Mac | DQ897969 | T | ● | ● | ● | ● | ● | T | ● | ● | ● | ● | ● | ● | ● | ● | ● | ● | ● | ● | ● | ● | ● | ● | ● | ● | ● | ● | A | ● | ● | ● | ● | ● | ● | ● | ● | ● | ● | ● | ● | ● | ● | ● | ● | ● | T | ● | ● | ● | ● | ● | ● | ● | ● | ● | ● | ● | ● | ● | ● |
|  | DQ897970 | T | ● | ● | ● | ● | ● | T | ● | ● | ● | ● | ● | ● | ● | ● | ● | ● | ● | ● | ● | ● | ● | ● | ● | ● | ● | ● | A | ● | ● | ● | ● | ● | ● | ● | ● | ● | ● | ● | ● | ● | ● | ● | ● | ● | T | ● | ● | ● | ● | ● | ● | ● | ● | ● | ● | ● | ● | ● | ● |
|  | DQ897971 | T | ● | ● | ● | ● | ● | T | ● | ● | ● | ● | ● | ● | ● | ● | ● | ● | ● | ● | ● | ● | ● | ● | ● | ● | ● | ● | A | ● | ● | ● | ● | ● | ● | ● | ● | ● | ● | ● | ● | ● | ● | ● | ● | ● | T | ● | ● | ● | ● | ● | ● | ● | ● | ● | ● | ● | ● | ● | ● |
|  | DQ897972 | T | ● | ● | ● | ● | ● | T | ● | ● | ● | ● | ● | ● | ● | ● | ● | ● | ● | ● | ● | ● | ● | ● | ● | ● | ● | ● | A | ● | ● | ● | ● | ● | ● | ● | ● | ● | ● | ● | ● | ● | ● | ● | ● | ● | T | ● | ● | ● | ● | ● | ● | ● | ● | ● | ● | ● | ● | ● | ● |
| Mir | DQ897965 | ● | ● | ● | ● | ● | ● | ● | ● | ● | ● | ● | ● | ● | ● | ● | A | ● | ● | ● | ● | ● | ● | ● | ● | T | ● | ● | A | ● | ● | ● | ● | ● | ● | ● | ● | ● | ● | ● | ● | ● | ● | ● | ● | ● | T | ● | ● | ● | ● | ● | ● | ● | ● | ● | ● | ● | ● | ● | ● |
|  | DQ897966 | ● | ● | ● | ● | ● | ● | G | ● | ● | ● | ● | ● | ● | ● | ● | A | ● | ● | ● | ● | ● | ● | ● | ● | ● | ● | ● | A | ● | ● | ● | ● | ● | ● | ● | ● | ● | ● | ● | ● | ● | ● | ● | ● | ● | T | ● | ● | ● | ● | ● | ● | ● | ● | ● | ● | ● | ● | ● | ● |
| Nem | DQ897959 | ● | ● | ● | ● | ● | ● | T | ● | ● | ● | ● | ● | ● | ● | ● | A | ● | ● | ● | ● | ● | ● | ● | ● | ● | ● | ● | A | ● | ● | ● | ● | ● | ● | ● | ● | ● | ● | ● | ● | ● | ● | ● | ● | ● | T | ● | ● | ● | ● | ● | ● | ● | ● | ● | ● | ● | ● | ● | ● |
|  | DQ897960 | ● | ● | ● | ● | ● | ● | T | ● | ● | ● | ● | ● | ● | ● | ● | A | ● | ● | ● | ● | ● | ● | ● | ● | T | ● | ● | A | ● | ● | ● | ● | ● | ● | ● | ● | ● | ● | ● | ● | ● | ● | ● | ● | ● | T | ● | ● | ● | ● | ● | ● | ● | ● | ● | ● | ● | ● | ● | ● |
|  | DQ897961 | ● | ● | ● | ● | ● | ● | C | ● | ● | ● | ● | ● | ● | ● | ● | ● | ● | ● | ● | ● | ● | ● | ● | ● | ● | ● | ● | A | ● | ● | ● | ● | ● | ● | ● | ● | ● | ● | ● | ● | ● | ● | ● | ● | ● | ● | ● | ● | ● | ● | ● | ● | ● | ● | ● | ● | ● | ● | ● | ● |
| Sca | DQ897949 | ● | ● | ● | ● | ● | ● | C | ● | ● | ● | ● | ● | ● | ● | ● | ● | ● | ● | ● | ● | ● | ● | ● | ● | ● | ● | ● | A | ● | ● | ● | ● | ● | ● | ● | ● | ● | ● | ● | ● | ● | ● | ● | ● | ● | ● | ● | ● | ● | ● | ● | ● | ● | ● | ● | ● | ● | ● | ● | ● |
| Tak cf | AB518534 | ● | ● | ● | ● | ● | ● | C | ● | ● | G | ● | ● | ● | ● | ● | ● | ● | ● | ● | ● | ● | ● | ● | ● | ● | ● | ● | A | ● | ● | ● | ● | ● | ● | ● | ● | ● | ● | ● | ● | ● | ● | ● | ● | ● | T | ● | ● | ● | ● | ● | ● | ● | ● | ● | ● | ● | ● | ● | ● |
|  | AB518535 | ● | ● | ● | ● | ● | ● | C | ● | ● | G | ● | ● | ● | ● | ● | ● | ● | ● | ● | ● | ● | ● | ● | ● | ● | ● | ● | A | ● | ● | ● | ● | ● | ● | ● | ● | ● | ● | ● | ● | ● | ● | ● | ● | ● | T | ● | ● | ● | ● | ● | ● | ● | ● | ● | ● | ● | ● | ● | ● |
|  | AB518536 | ● | ● | ● | ● | ● | ● | C | ● | ● | G | ● | ● | ● | ● | ● | ● | ● | ● | ● | ● | ● | ● | ● | ● | ● | ● | ● | A | ● | ● | ● | ● | ● | ● | ● | ● | ● | ● | ● | ● | ● | ● | ● | ● | ● | T | ● | ● | ● | ● | ● | ● | ● | ● | ● | ● | ● | ● | ● | ● |
|  | AB518537 | ● | ● | ● | ● | ● | ● | C | ● | ● | ● | ● | ● | ● | ● | ● | ● | ● | ● | ● | ● | ● | ● | ● | ● | ● | ● | ● | A | ● | ● | ● | ● | ● | ● | ● | ● | ● | ● | ● | ● | ● | ● | ● | ● | ● | T | ● | ● | ● | ● | ● | ● | ● | ● | ● | ● | ● | ● | ● | ● |
|  | AB518538 | ● | ● | ● | ● | ● | ● | C | ● | ● | G | ● | ● | ● | ● | ● | A | ● | ● | ● | ● | ● | ● | ● | ● | ● | ● | ● | A | ● | ● | ● | ● | ● | ● | ● | ● | ● | ● | ● | ● | ● | ● | ● | ● | ● | T | ● | ● | ● | ● | ● | ● | ● | ● | ● | ● | ● | ● | ● | ● |
| Tak | DQ897962 | ● | ● | ● | ● | ● | ● | C | ● | ● | ● | ● | ● | ● | ● | ● | ● | ● | ● | ● | ● | ● | ● | ● | ● | T | ● | ● | A | ● | ● | ● | ● | ● | ● | ● | ● | ● | ● | ● | ● | ● | ● | C | ● | ● | ● | ● | ● | ● | ● | ● | ● | ● | ● | ● | ● | ● | ● | ● | ● |
|  | DQ897963 | ● | ● | ● | ● | ● | ● | C | ● | ● | ● | ● | ● | ● | ● | ● | ● | ● | ● | ● | ● | ● | ● | ● | ● | T | ● | ● | A | ● | ● | ● | ● | ● | ● | ● | ● | ● | ● | ● | ● | ● | ● | C | ● | ● | ● | ● | ● | ● | ● | ● | ● | ● | ● | ● | ● | ● | ● | ● | ● |
|  | DQ897964 | ● | ● | ● | ● | ● | ● | C | ● | ● | ● | ● | ● | ● | ● | ● | ● | ● | ● | ● | ● | ● | ● | ● | ● | T | ● | ● | A | ● | ● | ● | ● | ● | ● | ● | ● | ● | ● | ● | ● | ● | ● | C | ● | ● | ● | ● | ● | ● | ● | ● | ● | ● | ● | ● | ● | ● | ● | ● | ● |

**Additional file 4: Alignment S2.** Multiple sequence alignment of the CO1 gene of *An. leucosphyrus* (*s.l*.) and an outgroup, *An. gambiae* (L20934).

*Bal = *An. balabacensi*s, Bai = *An. baimaii*, Cra = *An. cracens*, Dir = *An. dirus*, Ele = *An. elegans*, Gam = *An. gambiae*, Int = *An. introlatus*, Lat = *An. latens*, Leu = *An. leucosphyrus*, Mac = *An. macarthuri*, Mir = *An. mirans*, Nem = *An. nemophilous*, Sca = *An. scanloni*, Tak = *An. takasagoensis*, cf = confer

**● = nucleotide identical to sequence DQ897940

| Species* | Accession/  Sample  ID | Position of SNP (bp)* | | | | | | | | | | | | | | | | | | | | | | | | | | | | | | | | | | | | | | | | | | | | | | | | | | | | | | | | | | | |
| --- | --- | --- | --- | --- | --- | --- | --- | --- | --- | --- | --- | --- | --- | --- | --- | --- | --- | --- | --- | --- | --- | --- | --- | --- | --- | --- | --- | --- | --- | --- | --- | --- | --- | --- | --- | --- | --- | --- | --- | --- | --- | --- | --- | --- | --- | --- | --- | --- | --- | --- | --- | --- | --- | --- | --- | --- | --- | --- | --- | --- | --- |
|  |  |  |  |  |  |  |  |  |  |  |  |  |  |  |  |  |  |  |  |  |  |  |  |  |  |  |  |  |  |  |  |  |  |  |  |  |  |  |  |  | 1 | 1 | 1 | 1 | 1 | 1 | 1 | 1 | 1 | 1 | 1 | 1 | 1 | 1 | 1 | 1 | 1 | 1 | 1 | 1 | 1 |
|  |  | 6 | 6 | 6 | 6 | 6 | 6 | 6 | 6 | 6 | 7 | 7 | 7 | 7 | 7 | 7 | 7 | 7 | 7 | 7 | 8 | 8 | 8 | 8 | 8 | 8 | 8 | 8 | 8 | 8 | 9 | 9 | 9 | 9 | 9 | 9 | 9 | 9 | 9 | 9 | 0 | 0 | 0 | 0 | 0 | 0 | 0 | 0 | 0 | 0 | 1 | 1 | 1 | 1 | 1 | 1 | 1 | 1 | 1 | 1 | 2 |
|  |  | 1 | 2 | 3 | 4 | 5 | 6 | 7 | 8 | 9 | 0 | 1 | 2 | 3 | 4 | 5 | 6 | 7 | 8 | 9 | 0 | 1 | 2 | 3 | 4 | 5 | 6 | 7 | 8 | 9 | 0 | 1 | 2 | 3 | 4 | 5 | 6 | 7 | 8 | 9 | 0 | 1 | 2 | 3 | 4 | 5 | 6 | 7 | 8 | 9 | 0 | 1 | 2 | 3 | 4 | 5 | 6 | 7 | 8 | 9 | 0 |
| Bal | DQ897940 | T | G | T | A | T | C | T | A | C | T | T | T | A | G | G | T | A | G | A | A | C | A | A | T | T | T | C | A | C | T | A | T | T | T | G | C | T | A | T | T | T | T | A | T | A | C | T | T | T | T | T | A | T | T | T | A | T | T | A | T |
|  | DQ897941 | ● | ● | ● | ● | ● | ● | ● | ● | ● | ● | ● | ● | ● | ● | ● | ● | ● | ● | ● | ● | ● | ● | ● | ● | ● | ● | ● | ● | ● | ● | ● | ● | ● | ● | ● | ● | ● | ● | ● | ● | ● | ● | ● | ● | ● | ● | ● | ● | ● | ● | ● | ● | ● | ● | ● | ● | ● | ● | ● | ● |
|  | LW31 | ● | ● | ● | ● | ● | ● | ● | ● | ● | ● | ● | ● | ● | ● | ● | ● | ● | ● | ● | ● | ● | ● | ● | ● | ● | ● | ● | ● | ● | ● | ● | ● | ● | ● | ● | ● | ● | ● | ● | ● | ● | ● | ● | ● | ● | ● | ● | ● | ● | ● | ● | ● | ● | ● | ● | ● | ● | ● | ● | ● |
|  | LW32 | ● | ● | ● | ● | ● | ● | ● | ● | ● | ● | ● | ● | ● | ● | ● | ● | ● | ● | ● | ● | ● | ● | ● | ● | ● | ● | ● | ● | ● | ● | ● | ● | ● | ● | ● | ● | ● | ● | ● | ● | ● | ● | ● | ● | ● | ● | ● | ● | ● | ● | ● | ● | ● | ● | ● | ● | ● | ● | ● | ● |
|  | LW45 | ● | ● | ● | ● | ● | ● | ● | ● | ● | ● | ● | ● | ● | ● | ● | ● | ● | ● | ● | ● | ● | ● | ● | ● | ● | ● | ● | ● | ● | ● | ● | ● | ● | ● | ● | ● | ● | ● | ● | ● | ● | ● | ● | ● | ● | ● | ● | ● | ● | ● | ● | ● | ● | ● | ● | ● | ● | ● | ● | ● |
|  | LW49 | ● | ● | ● | ● | ● | ● | ● | ● | ● | ● | ● | ● | ● | ● | ● | ● | ● | ● | ● | ● | ● | ● | ● | ● | ● | ● | ● | ● | ● | ● | ● | ● | ● | ● | ● | ● | ● | ● | ● | ● | ● | ● | ● | ● | ● | ● | ● | ● | ● | ● | ● | ● | ● | ● | ● | ● | ● | ● | ● | ● |
|  | LW50 | ● | ● | ● | ● | ● | ● | ● | ● | ● | ● | ● | ● | ● | ● | ● | ● | ● | ● | ● | ● | ● | ● | ● | ● | ● | ● | ● | ● | ● | ● | ● | ● | ● | ● | ● | ● | ● | ● | ● | ● | ● | ● | ● | ● | ● | ● | ● | ● | ● | ● | ● | ● | ● | ● | ● | ● | ● | ● | ● | ● |
|  | LW51 | ● | ● | ● | ● | ● | ● | ● | ● | ● | ● | ● | ● | ● | ● | ● | ● | ● | ● | ● | ● | ● | ● | ● | ● | ● | ● | ● | ● | ● | ● | ● | ● | ● | ● | ● | ● | ● | ● | ● | ● | ● | ● | ● | ● | ● | ● | ● | ● | ● | ● | ● | ● | ● | ● | ● | ● | ● | ● | ● | ● |
|  | LW59 | ● | ● | ● | ● | ● | ● | ● | ● | ● | ● | ● | ● | ● | ● | ● | ● | ● | ● | ● | ● | ● | ● | ● | ● | ● | ● | ● | ● | ● | ● | ● | ● | ● | ● | ● | ● | ● | ● | ● | ● | ● | ● | ● | ● | ● | ● | ● | ● | ● | ● | ● | ● | ● | ● | ● | ● | ● | ● | ● | ● |
|  | LW67 | ● | ● | ● | ● | ● | ● | ● | ● | ● | ● | ● | ● | ● | ● | ● | ● | ● | ● | ● | ● | ● | ● | ● | ● | ● | ● | ● | ● | ● | ● | ● | ● | ● | ● | ● | ● | ● | ● | ● | ● | ● | ● | ● | ● | ● | ● | ● | ● | ● | ● | ● | ● | ● | ● | ● | ● | ● | ● | ● | ● |
|  | LW74 | ● | ● | ● | ● | ● | ● | ● | ● | ● | ● | ● | ● | ● | ● | ● | ● | ● | ● | ● | ● | ● | ● | ● | ● | ● | ● | ● | ● | ● | ● | ● | ● | ● | ● | ● | ● | ● | ● | ● | ● | ● | ● | ● | ● | ● | ● | ● | ● | ● | ● | ● | ● | ● | ● | ● | ● | ● | ● | ● | ● |
| Bai | DQ897952 | ● | ● | ● | ● | ● | ● | ● | ● | ● | ● | ● | ● | ● | ● | ● | ● | ● | ● | ● | ● | ● | ● | ● | ● | ● | ● | ● | ● | ● | ● | ● | ● | ● | ● | ● | ● | ● | ● | ● | ● | ● | ● | ● | ● | ● | ● | ● | ● | ● | ● | ● | ● | ● | ● | ● | ● | ● | ● | ● | ● |
|  | DQ897953 | ● | ● | ● | ● | ● | ● | ● | ● | ● | ● | ● | ● | ● | ● | ● | ● | ● | ● | ● | ● | ● | ● | ● | ● | ● | ● | ● | ● | ● | ● | ● | ● | ● | ● | ● | ● | ● | ● | ● | ● | ● | ● | ● | ● | ● | ● | ● | ● | ● | ● | ● | ● | ● | ● | ● | ● | ● | ● | ● | ● |
|  | DQ897954 | ● | ● | ● | ● | ● | ● | ● | ● | ● | ● | ● | ● | ● | ● | ● | ● | ● | ● | ● | ● | ● | ● | ● | ● | ● | ● | ● | ● | ● | ● | ● | ● | ● | ● | ● | ● | ● | ● | ● | ● | ● | ● | ● | ● | ● | ● | ● | ● | ● | ● | ● | ● | ● | ● | ● | ● | ● | ● | ● | ● |
|  | DQ897955 | ● | ● | ● | ● | ● | ● | ● | ● | ● | ● | ● | ● | ● | ● | ● | ● | ● | ● | ● | ● | ● | ● | ● | ● | ● | ● | ● | ● | ● | ● | ● | ● | ● | ● | ● | ● | ● | ● | ● | ● | ● | ● | ● | ● | ● | ● | ● | ● | ● | ● | ● | ● | ● | ● | ● | ● | ● | ● | ● | ● |
| Cra | DQ897947 | ● | ● | ● | ● | ● | ● | ● | ● | ● | ● | ● | ● | ● | ● | ● | ● | ● | ● | ● | ● | ● | ● | ● | ● | ● | ● | ● | ● | ● | ● | ● | ● | ● | ● | ● | ● | ● | ● | ● | ● | ● | ● | ● | ● | ● | ● | ● | ● | ● | ● | ● | ● | ● | ● | ● | ● | ● | ● | ● | ● |
|  | DQ897948 | ● | ● | ● | ● | ● | ● | ● | ● | ● | ● | ● | ● | ● | ● | ● | ● | ● | ● | ● | ● | ● | ● | ● | ● | ● | ● | ● | ● | ● | ● | ● | ● | ● | ● | ● | ● | ● | ● | ● | ● | ● | ● | ● | ● | ● | ● | ● | ● | ● | ● | ● | ● | ● | ● | ● | ● | ● | ● | ● | ● |
| Dir | AB518499 | ● | ● | ● | ● | ● | ● | ● | ● | ● | ● | ● | ● | ● | ● | ● | ● | ● | ● | ● | ● | ● | ● | ● | ● | ● | ● | ● | ● | ● | ● | ● | ● | ● | ● | ● | ● | ● | ● | ● | ● | ● | ● | ● | ● | ● | ● | ● | ● | ● | ● | ● | ● | ● | ● | ● | ● | ● | ● | ● | ● |
|  | AB518500 | ● | ● | ● | ● | ● | ● | ● | ● | ● | ● | ● | ● | ● | ● | ● | ● | ● | ● | ● | ● | ● | ● | ● | ● | ● | ● | ● | ● | ● | ● | ● | ● | ● | ● | ● | ● | ● | ● | ● | ● | ● | ● | ● | ● | ● | ● | ● | ● | ● | ● | ● | ● | ● | ● | ● | ● | ● | ● | ● | ● |
|  | AB518501 | ● | ● | ● | ● | ● | ● | ● | ● | ● | ● | ● | ● | ● | ● | ● | ● | ● | ● | ● | ● | ● | ● | ● | ● | ● | ● | ● | ● | ● | ● | ● | ● | ● | ● | ● | ● | ● | ● | ● | ● | ● | ● | ● | ● | ● | ● | ● | ● | ● | ● | ● | ● | ● | ● | ● | ● | ● | ● | ● | ● |
|  | AB518502 | ● | ● | ● | ● | ● | ● | ● | ● | ● | ● | ● | ● | ● | ● | ● | ● | ● | ● | ● | ● | ● | ● | ● | ● | ● | ● | ● | ● | ● | ● | ● | ● | ● | ● | ● | ● | ● | ● | ● | ● | ● | ● | ● | ● | ● | ● | ● | ● | ● | ● | ● | ● | ● | ● | ● | ● | ● | ● | ● | ● |
|  | AB518503 | ● | ● | ● | ● | ● | ● | ● | ● | ● | ● | ● | ● | ● | ● | ● | ● | ● | ● | ● | ● | ● | ● | ● | ● | ● | ● | ● | ● | ● | ● | ● | ● | ● | ● | ● | ● | ● | ● | ● | ● | ● | ● | ● | ● | ● | ● | ● | ● | ● | ● | ● | ● | ● | ● | ● | ● | ● | ● | ● | ● |
|  | AB518504 | ● | ● | ● | ● | ● | ● | ● | ● | ● | ● | ● | ● | ● | ● | ● | ● | ● | ● | ● | ● | ● | ● | ● | ● | ● | ● | ● | ● | ● | ● | ● | ● | ● | ● | ● | ● | ● | ● | ● | ● | ● | ● | ● | ● | ● | ● | ● | ● | ● | ● | ● | ● | ● | ● | ● | ● | ● | ● | ● | ● |
|  | AB518505 | ● | ● | ● | ● | ● | ● | ● | ● | ● | ● | ● | ● | ● | ● | ● | ● | ● | ● | ● | ● | ● | ● | ● | ● | ● | ● | ● | ● | ● | ● | ● | ● | ● | ● | ● | ● | ● | ● | ● | ● | ● | ● | ● | ● | ● | ● | ● | ● | ● | ● | ● | ● | ● | ● | ● | ● | ● | ● | ● | ● |
|  | AB518506 | ● | ● | ● | ● | ● | ● | ● | ● | ● | ● | ● | ● | ● | ● | ● | ● | ● | ● | ● | ● | ● | ● | ● | ● | ● | ● | ● | ● | ● | ● | ● | ● | ● | ● | ● | ● | ● | ● | ● | ● | ● | ● | ● | ● | ● | ● | ● | ● | ● | ● | ● | ● | ● | ● | ● | ● | ● | ● | ● | ● |
|  | AB518507 | ● | ● | ● | ● | ● | ● | ● | ● | ● | ● | ● | ● | ● | ● | ● | ● | ● | ● | ● | ● | ● | ● | ● | ● | ● | ● | ● | ● | ● | ● | ● | ● | ● | ● | ● | ● | ● | ● | ● | ● | ● | ● | ● | ● | ● | ● | ● | ● | ● | ● | ● | ● | ● | ● | ● | ● | ● | ● | ● | ● |
|  | AB518508 | ● | ● | ● | ● | ● | ● | ● | ● | ● | ● | ● | ● | ● | ● | ● | ● | ● | ● | ● | ● | ● | ● | ● | ● | ● | ● | ● | ● | ● | ● | ● | ● | ● | ● | ● | ● | ● | ● | ● | ● | ● | ● | ● | ● | ● | ● | ● | ● | ● | ● | ● | ● | ● | ● | ● | ● | ● | ● | ● | ● |
|  | AB518509 | ● | ● | ● | ● | ● | ● | ● | ● | ● | ● | ● | ● | ● | ● | ● | ● | ● | ● | ● | ● | ● | ● | ● | ● | ● | ● | ● | ● | ● | ● | ● | ● | ● | ● | ● | ● | ● | ● | ● | ● | ● | ● | ● | ● | ● | ● | ● | ● | ● | ● | ● | ● | ● | ● | ● | ● | ● | ● | ● | ● |
|  | AB518510 | ● | ● | ● | ● | ● | ● | ● | ● | ● | ● | ● | ● | ● | ● | ● | ● | ● | ● | ● | ● | ● | ● | ● | ● | ● | ● | ● | ● | ● | ● | ● | ● | ● | ● | ● | ● | ● | ● | ● | ● | ● | ● | ● | ● | ● | ● | ● | ● | ● | ● | ● | ● | ● | ● | ● | ● | ● | ● | ● | ● |
|  | AB518511 | ● | ● | ● | ● | ● | ● | ● | ● | ● | ● | ● | ● | ● | ● | ● | ● | ● | ● | ● | ● | ● | ● | ● | ● | ● | ● | ● | ● | ● | ● | ● | ● | ● | ● | ● | ● | ● | ● | ● | ● | ● | ● | ● | ● | ● | ● | ● | ● | ● | ● | ● | ● | ● | ● | ● | ● | ● | ● | ● | ● |
|  | AB518512 | ● | ● | ● | ● | ● | ● | ● | ● | ● | ● | ● | ● | ● | ● | ● | ● | ● | ● | ● | ● | ● | ● | ● | ● | ● | ● | ● | ● | ● | ● | ● | ● | ● | ● | ● | ● | ● | ● | ● | ● | ● | ● | ● | ● | ● | ● | ● | ● | ● | ● | ● | ● | ● | ● | ● | ● | ● | ● | ● | ● |
|  | AB518513 | ● | ● | ● | ● | ● | ● | ● | ● | ● | ● | ● | ● | ● | ● | ● | ● | ● | ● | ● | ● | ● | ● | ● | ● | ● | ● | ● | ● | ● | ● | ● | ● | ● | ● | ● | ● | ● | ● | ● | ● | ● | ● | ● | ● | ● | ● | ● | ● | ● | ● | ● | ● | ● | ● | ● | ● | ● | ● | ● | ● |
|  | AB518514 | ● | ● | ● | ● | ● | ● | ● | ● | ● | ● | ● | ● | ● | ● | ● | ● | ● | ● | ● | ● | ● | ● | ● | ● | ● | ● | ● | ● | ● | ● | ● | ● | ● | ● | ● | ● | ● | ● | ● | ● | ● | ● | ● | ● | ● | ● | ● | ● | ● | ● | ● | ● | ● | ● | ● | ● | ● | ● | ● | ● |
|  | AB518515 | ● | ● | ● | ● | ● | ● | ● | ● | ● | ● | ● | ● | ● | ● | ● | ● | ● | ● | ● | ● | ● | ● | ● | ● | ● | ● | ● | ● | ● | ● | ● | ● | ● | ● | ● | ● | ● | ● | ● | ● | ● | ● | ● | ● | ● | ● | ● | ● | ● | ● | ● | ● | ● | ● | ● | ● | ● | ● | ● | ● |
|  | AB518516 | ● | ● | ● | ● | ● | ● | ● | ● | ● | ● | ● | ● | ● | ● | ● | ● | ● | ● | ● | ● | ● | ● | ● | ● | ● | ● | ● | ● | ● | ● | ● | ● | ● | ● | ● | ● | ● | ● | ● | ● | ● | ● | ● | ● | ● | ● | ● | ● | ● | ● | ● | ● | ● | ● | ● | ● | ● | ● | ● | ● |
|  | AB518517 | ● | ● | ● | ● | ● | ● | ● | ● | ● | ● | ● | ● | ● | ● | ● | ● | ● | ● | ● | ● | ● | ● | ● | ● | ● | ● | ● | ● | ● | ● | ● | ● | ● | ● | ● | ● | ● | ● | ● | ● | ● | ● | ● | ● | ● | ● | ● | ● | ● | ● | ● | ● | ● | ● | ● | ● | ● | ● | ● | ● |
|  | AB518518 | ● | ● | ● | ● | ● | ● | ● | ● | ● | ● | ● | ● | ● | ● | ● | ● | ● | ● | ● | ● | ● | ● | ● | ● | ● | ● | ● | ● | ● | ● | ● | ● | ● | ● | ● | ● | ● | ● | ● | ● | ● | ● | ● | ● | ● | ● | ● | ● | ● | ● | ● | ● | ● | ● | ● | ● | ● | ● | ● | ● |
|  | AB518519 | ● | ● | ● | ● | ● | ● | ● | ● | ● | ● | ● | ● | ● | ● | ● | ● | ● | ● | ● | ● | ● | ● | ● | ● | ● | ● | ● | ● | ● | ● | ● | ● | ● | ● | ● | ● | ● | ● | ● | ● | ● | ● | ● | ● | ● | ● | ● | ● | ● | ● | ● | ● | ● | ● | ● | ● | ● | ● | ● | ● |
|  | AB518520 | ● | ● | ● | ● | ● | ● | ● | ● | ● | ● | ● | ● | ● | ● | ● | ● | ● | ● | ● | ● | ● | ● | ● | ● | ● | ● | ● | ● | ● | ● | ● | ● | ● | ● | ● | ● | ● | ● | ● | ● | ● | ● | ● | ● | ● | ● | ● | ● | ● | ● | ● | ● | ● | ● | ● | ● | ● | ● | ● | ● |
|  | AB518521 | ● | ● | ● | ● | ● | ● | ● | ● | ● | ● | ● | ● | ● | ● | ● | ● | ● | ● | ● | ● | ● | ● | ● | ● | ● | ● | ● | ● | ● | ● | ● | ● | ● | ● | ● | ● | ● | ● | ● | ● | ● | ● | ● | ● | ● | ● | ● | ● | ● | ● | ● | ● | ● | ● | ● | ● | ● | ● | ● | ● |
|  | AB518522 | ● | ● | ● | ● | ● | ● | ● | ● | ● | ● | ● | ● | ● | ● | ● | ● | ● | ● | ● | ● | ● | ● | ● | ● | ● | ● | ● | ● | ● | ● | ● | ● | ● | ● | ● | ● | ● | ● | ● | ● | ● | ● | ● | ● | ● | ● | ● | ● | ● | ● | ● | ● | ● | ● | ● | ● | ● | ● | ● | ● |
|  | AB518523 | ● | ● | ● | ● | ● | ● | ● | ● | ● | ● | ● | ● | ● | ● | ● | ● | ● | ● | ● | ● | ● | ● | ● | ● | ● | ● | ● | ● | ● | ● | ● | ● | ● | ● | ● | ● | ● | ● | ● | ● | ● | ● | ● | ● | ● | ● | ● | ● | ● | ● | ● | ● | ● | ● | ● | ● | ● | ● | ● | ● |
|  | AB518524 | ● | ● | ● | ● | ● | ● | ● | ● | ● | ● | ● | ● | ● | ● | ● | ● | ● | ● | ● | ● | ● | ● | ● | ● | ● | ● | ● | ● | ● | ● | ● | ● | ● | ● | ● | ● | ● | ● | ● | ● | ● | ● | ● | ● | ● | ● | ● | ● | ● | ● | ● | ● | ● | ● | ● | ● | ● | ● | ● | ● |
|  | AB518525 | ● | ● | ● | ● | ● | ● | ● | ● | ● | ● | ● | ● | ● | ● | ● | ● | ● | ● | ● | ● | ● | ● | ● | ● | ● | ● | ● | ● | ● | ● | ● | ● | ● | ● | ● | ● | ● | ● | ● | ● | ● | ● | ● | ● | ● | ● | ● | ● | ● | ● | ● | ● | ● | ● | ● | ● | ● | ● | ● | ● |
|  | AB518526 | ● | ● | ● | ● | ● | ● | ● | ● | ● | ● | ● | ● | ● | ● | ● | ● | ● | ● | ● | ● | ● | ● | ● | ● | ● | ● | ● | ● | ● | ● | ● | ● | ● | ● | ● | ● | ● | ● | ● | ● | ● | ● | ● | ● | ● | ● | ● | ● | ● | ● | ● | ● | ● | ● | ● | ● | ● | ● | ● | ● |
|  | AB518527 | ● | ● | ● | ● | ● | ● | ● | ● | ● | ● | ● | ● | ● | ● | ● | ● | ● | ● | ● | ● | ● | ● | ● | ● | ● | ● | ● | ● | ● | ● | ● | ● | ● | ● | ● | ● | ● | ● | ● | ● | ● | ● | ● | ● | ● | ● | ● | ● | ● | ● | ● | ● | ● | ● | ● | ● | ● | ● | ● | ● |
|  | AB518528 | ● | ● | ● | ● | ● | ● | ● | ● | ● | ● | ● | ● | ● | ● | ● | ● | ● | ● | ● | ● | ● | ● | ● | ● | ● | ● | ● | ● | ● | ● | ● | ● | ● | ● | ● | ● | ● | ● | ● | ● | ● | ● | ● | ● | ● | ● | ● | ● | ● | ● | ● | ● | ● | ● | ● | ● | ● | ● | ● | ● |
|  | AB518529 | ● | ● | ● | ● | ● | ● | ● | ● | ● | ● | ● | ● | ● | ● | ● | ● | ● | ● | ● | ● | ● | ● | ● | ● | ● | ● | ● | ● | ● | ● | ● | ● | ● | ● | ● | ● | ● | ● | ● | ● | ● | ● | ● | ● | ● | ● | ● | ● | ● | ● | ● | ● | ● | ● | ● | ● | ● | ● | ● | ● |
|  | AB518530 | ● | ● | ● | ● | ● | ● | ● | ● | ● | ● | ● | ● | ● | ● | ● | ● | ● | ● | ● | ● | ● | ● | ● | ● | ● | ● | ● | ● | ● | ● | ● | ● | ● | ● | ● | ● | ● | ● | ● | ● | ● | ● | ● | ● | ● | ● | ● | ● | ● | ● | ● | ● | ● | ● | ● | ● | ● | ● | ● | ● |
|  | AB518531 | ● | ● | ● | ● | ● | ● | ● | ● | ● | ● | ● | ● | ● | ● | ● | ● | ● | ● | ● | ● | ● | ● | ● | ● | ● | ● | ● | ● | ● | ● | ● | ● | ● | ● | ● | ● | ● | ● | ● | ● | ● | ● | ● | ● | ● | ● | ● | ● | ● | ● | ● | ● | ● | ● | ● | ● | ● | ● | ● | ● |
|  | AB518532 | ● | ● | ● | ● | ● | ● | ● | ● | ● | ● | ● | ● | ● | ● | ● | ● | ● | ● | ● | ● | ● | ● | ● | ● | ● | ● | ● | ● | ● | ● | ● | ● | ● | ● | ● | ● | ● | ● | ● | ● | ● | ● | ● | ● | ● | ● | ● | ● | ● | ● | ● | ● | ● | ● | ● | ● | ● | ● | ● | ● |
|  | AB518533 | ● | ● | ● | ● | ● | ● | ● | ● | ● | ● | ● | ● | ● | ● | ● | ● | ● | ● | ● | ● | ● | ● | ● | ● | ● | ● | ● | ● | ● | ● | ● | ● | ● | ● | ● | ● | ● | ● | ● | ● | ● | ● | ● | ● | ● | ● | ● | ● | ● | ● | ● | ● | ● | ● | ● | ● | ● | ● | ● | ● |
|  | DQ897944 | ● | ● | ● | ● | ● | ● | ● | ● | ● | ● | ● | ● | ● | ● | ● | ● | ● | ● | ● | ● | ● | ● | ● | ● | ● | ● | ● | ● | ● | ● | ● | ● | ● | ● | ● | ● | ● | ● | ● | ● | ● | ● | ● | ● | ● | ● | ● | ● | ● | ● | ● | ● | ● | ● | ● | ● | ● | ● | ● | ● |
|  | DQ897945 | ● | ● | ● | ● | ● | ● | ● | ● | ● | ● | ● | ● | ● | ● | ● | ● | ● | ● | ● | ● | ● | ● | ● | ● | ● | ● | ● | ● | ● | ● | ● | ● | ● | ● | ● | ● | ● | ● | ● | ● | ● | ● | ● | ● | ● | ● | ● | ● | ● | ● | ● | ● | ● | ● | ● | ● | ● | ● | ● | ● |
|  | DQ897946 | ● | ● | ● | ● | ● | ● | ● | ● | ● | ● | ● | ● | ● | ● | ● | ● | ● | ● | ● | ● | ● | ● | ● | ● | ● | ● | ● | ● | ● | ● | ● | ● | ● | ● | ● | ● | ● | ● | ● | ● | ● | ● | ● | ● | ● | ● | ● | ● | ● | ● | ● | ● | ● | ● | ● | ● | ● | ● | ● | ● |
| Ele | DQ897957 | ● | ● | ● | ● | ● | ● | ● | ● | ● | ● | ● | ● | ● | ● | ● | ● | ● | ● | ● | ● | ● | ● | ● | ● | ● | ● | ● | ● | ● | ● | ● | ● | ● | ● | ● | ● | ● | ● | ● | ● | ● | ● | ● | ● | ● | ● | ● | ● | ● | ● | ● | ● | ● | ● | ● | ● | ● | ● | ● | ● |
|  | DQ897958 | ● | ● | ● | ● | ● | ● | ● | ● | ● | ● | ● | ● | ● | ● | ● | ● | ● | ● | ● | ● | ● | ● | ● | ● | ● | ● | ● | ● | ● | ● | ● | ● | ● | ● | ● | ● | ● | ● | ● | ● | ● | ● | ● | ● | ● | ● | ● | ● | ● | ● | ● | ● | ● | ● | ● | ● | ● | ● | ● | ● |
| Gam | L20934 | A | ● | ● | T | ● | ● | ● | T | ● | ● | ● | ● | ● | ● | ● | ● | ● | ● | T | ● | ● | ● | ● | ● | C | ● | ● | ● | T | ● | ● | ● | ● | C | ● | ● | ● | ● | ● | ● | ● | ● | ● | ● | ● | ● | ● | ● | ● | ● | ● | ● | ● | ● | ● | ● | ● | ● | ● | ● |
| Int | KM032605 | ● | ● | ● | G | ● | ● | ● | ● | ● | C | ● | ● | ● | ● | ● | ● | ● | ● | ● | ● | ● | ● | ● | ● | ● | ● | ● | ● | T | ● | ● | ● | ● | ● | ● | ● | ● | ● | ● | ● | ● | ● | ● | ● | ● | ● | ● | ● | ● | ● | ● | ● | ● | ● | ● | ● | ● | ● | ● | ● |
|  | KM032606 | ● | ● | ● | G | ● | ● | ● | ● | ● | C | ● | ● | ● | ● | ● | ● | ● | ● | ● | ● | ● | ● | ● | ● | ● | ● | ● | ● | T | ● | ● | ● | ● | ● | ● | ● | ● | ● | ● | ● | ● | ● | ● | ● | ● | ● | ● | ● | ● | ● | ● | ● | ● | ● | ● | ● | ● | ● | ● | ● |
|  | KM032607 | ● | ● | ● | G | ● | ● | ● | ● | ● | C | ● | ● | ● | ● | ● | ● | ● | ● | ● | ● | ● | ● | ● | ● | ● | ● | ● | ● | T | ● | ● | ● | ● | ● | ● | ● | ● | ● | ● | ● | ● | ● | ● | ● | ● | ● | ● | ● | ● | ● | ● | ● | ● | ● | ● | ● | ● | ● | ● | ● |
|  | KM032608 | ● | ● | ● | G | ● | ● | ● | ● | ● | C | ● | ● | ● | ● | ● | ● | ● | ● | ● | ● | ● | ● | ● | ● | ● | ● | ● | ● | T | ● | ● | ● | ● | ● | ● | ● | ● | ● | ● | ● | ● | ● | ● | ● | ● | ● | ● | ● | ● | ● | ● | ● | ● | ● | ● | ● | ● | ● | ● | ● |
|  | KM032609 | ● | ● | ● | G | ● | ● | ● | ● | ● | C | ● | ● | ● | ● | ● | ● | ● | ● | ● | ● | ● | ● | ● | ● | ● | ● | ● | ● | T | ● | ● | ● | ● | ● | ● | ● | ● | ● | ● | ● | ● | ● | ● | ● | ● | ● | ● | ● | ● | ● | ● | ● | ● | ● | ● | ● | ● | ● | ● | ● |
|  | KM032610 | ● | ● | ● | ● | ● | ● | ● | ● | ● | C | ● | ● | ● | ● | ● | ● | ● | ● | ● | ● | ● | ● | ● | ● | ● | ● | ● | ● | T | ● | ● | ● | ● | ● | ● | ● | ● | ● | ● | ● | ● | ● | ● | ● | ● | ● | ● | ● | ● | ● | ● | ● | ● | ● | ● | ● | ● | ● | ● | ● |
|  | KM032611 | ● | ● | ● | G | ● | ● | ● | ● | ● | C | ● | ● | ● | ● | ● | ● | ● | ● | ● | ● | ● | ● | ● | ● | ● | ● | ● | ● | T | ● | ● | ● | ● | ● | ● | ● | ● | ● | ● | ● | ● | ● | ● | ● | ● | ● | ● | ● | ● | ● | ● | ● | ● | ● | ● | ● | ● | ● | ● | ● |
|  | KM032612 | ● | ● | ● | ● | ● | ● | ● | ● | ● | C | ● | ● | ● | ● | ● | ● | ● | ● | ● | ● | ● | ● | ● | ● | ● | ● | ● | ● | T | ● | ● | ● | ● | ● | ● | ● | ● | ● | ● | ● | ● | ● | ● | ● | ● | ● | ● | ● | ● | ● | ● | ● | ● | ● | ● | ● | ● | ● | ● | ● |
| Lat | DQ897936 | ● | ● | ● | ● | ● | ● | ● | ● | ● | ● | ● | ● | ● | ● | ● | ● | ● | ● | ● | ● | ● | ● | ● | ● | ● | ● | ● | ● | T | ● | ● | ● | ● | ● | ● | ● | ● | ● | ● | ● | ● | ● | ● | ● | ● | ● | ● | ● | ● | ● | ● | ● | ● | ● | ● | ● | ● | ● | ● | ● |
|  | DQ897937 | ● | ● | ● | ● | ● | ● | ● | ● | ● | ● | ● | ● | ● | ● | ● | ● | ● | ● | ● | ● | ● | ● | ● | ● | ● | ● | ● | ● | T | ● | ● | ● | ● | ● | ● | ● | ● | ● | ● | ● | ● | ● | ● | ● | ● | T | ● | ● | ● | ● | ● | ● | ● | ● | ● | ● | ● | ● | ● | ● |
| Leu | DQ897939 | ● | ● | ● | ● | ● | ● | ● | ● | ● | ● | ● | ● | ● | ● | ● | ● | ● | ● | ● | ● | ● | ● | ● | ● | ● | ● | ● | ● | T | ● | ● | ● | ● | ● | ● | ● | ● | ● | ● | ● | ● | ● | ● | ● | ● | ● | ● | ● | ● | ● | ● | ● | ● | ● | ● | ● | ● | ● | ● | ● |
| Mac | DQ897969 | ● | ● | ● | ● | ● | ● | ● | ● | ● | ● | ● | ● | ● | ● | ● | ● | ● | ● | ● | ● | ● | T | ● | ● | C | ● | ● | ● | T | ● | ● | ● | ● | ● | ● | ● | ● | ● | ● | ● | ● | ● | ● | ● | ● | T | ● | ● | ● | ● | ● | ● | ● | ● | ● | ● | ● | ● | ● | ● |
|  | DQ897970 | ● | ● | ● | ● | ● | ● | ● | ● | ● | ● | ● | ● | ● | ● | ● | ● | ● | ● | ● | ● | ● | T | ● | ● | C | ● | ● | ● | T | ● | ● | ● | ● | ● | ● | ● | ● | ● | ● | ● | ● | ● | ● | ● | ● | T | ● | ● | ● | ● | ● | ● | ● | ● | ● | ● | ● | ● | ● | ● |
|  | DQ897971 | ● | ● | ● | ● | ● | ● | ● | ● | ● | ● | ● | ● | ● | ● | ● | ● | ● | ● | ● | ● | ● | T | ● | ● | C | ● | ● | ● | T | ● | ● | ● | ● | ● | ● | ● | ● | ● | ● | ● | ● | ● | ● | ● | ● | T | ● | ● | ● | ● | ● | ● | ● | ● | ● | ● | ● | ● | ● | ● |
|  | DQ897972 | ● | ● | ● | ● | ● | ● | ● | ● | ● | ● | ● | ● | ● | ● | ● | ● | ● | ● | ● | ● | ● | T | ● | ● | C | ● | ● | ● | T | ● | ● | ● | ● | ● | ● | ● | ● | ● | ● | ● | ● | ● | ● | ● | ● | T | ● | ● | ● | ● | ● | ● | ● | ● | ● | ● | ● | ● | ● | ● |
| Mir | DQ897965 | ● | ● | ● | T | ● | ● | A | ● | ● | ● | ● | ● | ● | ● | ● | ● | ● | ● | ● | ● | ● | ● | ● | ● | ● | ● | ● | ● | T | ● | ● | ● | ● | ● | ● | ● | ● | ● | ● | ● | ● | ● | ● | ● | ● | ● | ● | ● | ● | ● | ● | ● | ● | ● | ● | ● | ● | ● | ● | ● |
|  | DQ897966 | ● | ● | ● | T | ● | ● | A | ● | ● | ● | ● | ● | ● | ● | ● | ● | ● | ● | ● | ● | ● | ● | ● | ● | ● | ● | ● | ● | T | ● | ● | ● | ● | ● | ● | ● | ● | ● | ● | ● | ● | ● | ● | ● | ● | ● | ● | ● | ● | ● | ● | ● | ● | ● | ● | ● | ● | ● | ● | ● |
| Nem | DQ897959 | ● | ● | ● | ● | ● | ● | ● | ● | ● | C | ● | ● | ● | ● | ● | ● | ● | ● | ● | ● | ● | ● | ● | ● | ● | ● | ● | ● | T | ● | ● | ● | ● | ● | ● | ● | ● | ● | ● | ● | ● | ● | ● | ● | ● | ● | ● | ● | ● | ● | ● | ● | ● | ● | ● | ● | ● | ● | ● | ● |
|  | DQ897960 | ● | ● | ● | ● | ● | ● | ● | ● | ● | C | ● | ● | G | ● | ● | ● | ● | ● | ● | ● | ● | ● | ● | ● | ● | ● | ● | ● | T | ● | ● | ● | ● | ● | ● | ● | ● | ● | ● | ● | ● | ● | ● | ● | ● | ● | ● | ● | ● | ● | ● | ● | ● | ● | ● | ● | ● | ● | ● | ● |
|  | DQ897961 | ● | ● | ● | ● | ● | ● | ● | ● | ● | ● | ● | ● | ● | ● | ● | ● | ● | ● | ● | ● | ● | ● | ● | ● | ● | ● | ● | ● | ● | ● | ● | ● | ● | ● | ● | ● | ● | ● | ● | ● | ● | ● | ● | ● | ● | ● | ● | ● | ● | ● | ● | ● | ● | ● | ● | ● | ● | ● | ● | ● |
| Sca | DQ897949 | ● | ● | ● | ● | ● | ● | ● | ● | ● | ● | ● | ● | ● | ● | ● | ● | ● | ● | ● | ● | ● | ● | ● | ● | ● | ● | ● | ● | ● | ● | ● | ● | ● | ● | ● | ● | ● | ● | ● | ● | ● | ● | ● | ● | ● | ● | ● | ● | ● | ● | ● | G | ● | ● | ● | ● | ● | ● | ● | ● |
| Tak cf | AB518534 | ● | ● | ● | ● | ● | ● | ● | ● | ● | ● | ● | ● | ● | ● | ● | ● | ● | ● | ● | ● | ● | ● | ● | ● | ● | ● | ● | ● | ● | ● | ● | ● | ● | ● | ● | ● | ● | ● | ● | ● | ● | ● | ● | ● | ● | ● | ● | ● | ● | ● | ● | ● | ● | ● | ● | ● | ● | ● | ● | ● |
|  | AB518535 | ● | A | ● | ● | ● | ● | ● | ● | ● | ● | ● | ● | ● | ● | ● | ● | ● | ● | ● | ● | ● | ● | ● | ● | ● | ● | ● | ● | ● | ● | ● | ● | ● | ● | ● | ● | ● | ● | ● | ● | ● | ● | ● | ● | ● | ● | ● | ● | ● | ● | ● | ● | ● | ● | ● | ● | ● | ● | ● | ● |
|  | AB518536 | ● | ● | ● | ● | ● | ● | ● | ● | ● | ● | ● | ● | ● | ● | ● | ● | ● | ● | ● | ● | ● | ● | ● | ● | ● | ● | ● | ● | ● | ● | ● | ● | ● | ● | ● | ● | ● | ● | ● | ● | ● | ● | ● | ● | ● | ● | ● | ● | ● | ● | ● | ● | ● | ● | ● | ● | ● | ● | ● | ● |
|  | AB518537 | ● | ● | ● | ● | ● | ● | ● | ● | ● | ● | ● | ● | ● | ● | ● | ● | ● | ● | ● | ● | ● | ● | ● | ● | ● | ● | ● | ● | ● | ● | ● | ● | ● | ● | ● | ● | ● | ● | ● | ● | ● | ● | ● | ● | ● | ● | ● | ● | ● | ● | ● | ● | ● | ● | ● | ● | ● | ● | ● | ● |
|  | AB518538 | ● | ● | ● | ● | ● | ● | ● | ● | ● | ● | ● | ● | ● | ● | ● | ● | ● | ● | ● | ● | ● | ● | ● | ● | ● | ● | ● | ● | ● | ● | ● | ● | ● | ● | ● | ● | ● | ● | ● | ● | ● | ● | ● | ● | ● | ● | ● | ● | ● | ● | ● | ● | ● | ● | ● | ● | ● | ● | ● | ● |
| Tak | DQ897962 | ● | ● | ● | ● | ● | ● | ● | ● | ● | ● | ● | ● | ● | ● | ● | ● | ● | ● | ● | ● | ● | ● | ● | ● | ● | ● | ● | ● | ● | ● | ● | ● | ● | ● | ● | ● | ● | ● | ● | ● | ● | ● | ● | ● | ● | ● | ● | ● | ● | ● | ● | ● | ● | ● | ● | ● | ● | ● | ● | ● |
|  | DQ897963 | ● | ● | ● | ● | ● | ● | ● | ● | ● | ● | ● | ● | ● | ● | ● | ● | ● | ● | ● | ● | ● | ● | ● | ● | ● | ● | ● | ● | ● | ● | ● | ● | ● | ● | ● | ● | ● | ● | ● | ● | ● | ● | ● | ● | ● | ● | ● | ● | ● | ● | ● | ● | ● | ● | ● | ● | ● | ● | ● | ● |
|  | DQ897964 | ● | ● | ● | ● | ● | ● | ● | ● | ● | ● | ● | ● | ● | ● | ● | ● | ● | ● | ● | ● | ● | ● | ● | ● | ● | ● | ● | ● | ● | ● | ● | ● | ● | ● | ● | ● | ● | ● | ● | ● | ● | ● | ● | ● | ● | ● | ● | ● | ● | ● | ● | ● | ● | ● | ● | ● | ● | ● | ● | ● |

Bal = *An. balabacensi*s, Bai = *An. baimaii*, Cra = *An. cracens*, Dir = *An. dirus*, Ele = *An. elegans*, Gam = *An. gambiae*, Int = *An. introlatus*, Lat = *An. latens*, Leu = *An. leucosphyrus*, Mac = *An. macarthuri*, Mir = *An. mirans*, Nem = *An. nemophilous*, Sca = *An. scanloni*, Tak = *An. takasagoensis*, cf = confer

**● = nucleotide identical to sequence DQ897940

| Species* | Accession/  Sample  ID | Position of SNP (bp)** | | | | | | | | | | | | | | | | | | | | | | | | | | | | | | | | | | | | | | | | | | | | | | | | | | | | | | | | | | | |
| --- | --- | --- | --- | --- | --- | --- | --- | --- | --- | --- | --- | --- | --- | --- | --- | --- | --- | --- | --- | --- | --- | --- | --- | --- | --- | --- | --- | --- | --- | --- | --- | --- | --- | --- | --- | --- | --- | --- | --- | --- | --- | --- | --- | --- | --- | --- | --- | --- | --- | --- | --- | --- | --- | --- | --- | --- | --- | --- | --- | --- | --- |
|  |  | 1 | 1 | 1 | 1 | 1 | 1 | 1 | 1 | 1 | 1 | 1 | 1 | 1 | 1 | 1 | 1 | 1 | 1 | 1 | 1 | 1 | 1 | 1 | 1 | 1 | 1 | 1 | 1 | 1 | 1 | 1 | 1 | 1 | 1 | 1 | 1 | 1 | 1 | 1 | 1 | 1 | 1 | 1 | 1 | 1 | 1 | 1 | 1 | 1 | 1 | 1 | 1 | 1 | 1 | 1 | 1 | 1 | 1 | 1 | 1 |
|  |  | 2 | 2 | 2 | 2 | 2 | 2 | 2 | 2 | 2 | 3 | 3 | 3 | 3 | 3 | 3 | 3 | 3 | 3 | 3 | 4 | 4 | 4 | 4 | 4 | 4 | 4 | 4 | 4 | 4 | 5 | 5 | 5 | 5 | 5 | 5 | 5 | 5 | 5 | 5 | 6 | 6 | 6 | 6 | 6 | 6 | 6 | 6 | 6 | 6 | 7 | 7 | 7 | 7 | 7 | 7 | 7 | 7 | 7 | 7 | 8 |
|  |  | 1 | 2 | 3 | 4 | 5 | 6 | 7 | 8 | 9 | 0 | 1 | 2 | 3 | 4 | 5 | 6 | 7 | 8 | 9 | 0 | 1 | 2 | 3 | 4 | 5 | 6 | 7 | 8 | 9 | 0 | 1 | 2 | 3 | 4 | 5 | 6 | 7 | 8 | 9 | 0 | 1 | 2 | 3 | 4 | 5 | 6 | 7 | 8 | 9 | 0 | 1 | 2 | 3 | 4 | 5 | 6 | 7 | 8 | 9 | 0 |
| Bal | DQ897940 | C | T | G | A | G | A | A | A | G | T | A | T | A | A | T | T | A | C | T | C | A | A | C | G | A | A | C | T | C | C | T | G | C | T | T | T | T | C | C | T | A | T | A | C | A | A | C | T | T | T | C | T | T | C | A | T | C | T | A | T |
|  | DQ897941 | ● | ● | ● | ● | ● | ● | ● | ● | ● | ● | ● | ● | ● | ● | ● | ● | ● | ● | ● | ● | ● | ● | ● | ● | ● | ● | ● | ● | ● | ● | ● | ● | ● | ● | ● | ● | ● | ● | ● | ● | ● | ● | ● | ● | ● | ● | ● | ● | ● | ● | ● | ● | ● | ● | ● | ● | ● | ● | ● | ● |
|  | LW31 | ● | ● | ● | ● | ● | ● | ● | ● | ● | ● | ● | ● | ● | ● | ● | ● | ● | ● | ● | ● | ● | ● | ● | ● | ● | ● | ● | ● | ● | ● | ● | ● | ● | ● | ● | ● | ● | ● | ● | ● | ● | ● | ● | ● | ● | ● | ● | ● | ● | ● | ● | ● | ● | ● | ● | ● | ● | ● | ● | ● |
|  | LW32 | ● | ● | ● | ● | ● | ● | ● | ● | ● | ● | ● | ● | ● | ● | ● | ● | ● | ● | ● | ● | ● | ● | ● | ● | ● | ● | ● | ● | ● | ● | ● | ● | ● | ● | ● | ● | ● | ● | ● | ● | ● | ● | ● | ● | ● | ● | ● | ● | ● | ● | ● | ● | ● | ● | ● | ● | ● | ● | ● | ● |
|  | LW45 | ● | ● | ● | ● | ● | ● | ● | ● | ● | ● | ● | ● | ● | ● | ● | ● | ● | ● | ● | ● | ● | ● | ● | ● | ● | ● | ● | ● | ● | ● | ● | ● | ● | ● | ● | ● | ● | ● | ● | ● | ● | ● | ● | ● | ● | ● | ● | ● | ● | ● | ● | ● | ● | ● | ● | ● | ● | ● | ● | ● |
|  | LW49 | ● | ● | ● | ● | ● | ● | ● | ● | ● | ● | ● | ● | ● | ● | ● | ● | ● | ● | ● | ● | ● | ● | ● | ● | ● | ● | ● | ● | ● | ● | ● | ● | ● | ● | ● | ● | ● | ● | ● | ● | ● | ● | ● | ● | ● | ● | ● | ● | ● | ● | ● | ● | ● | ● | ● | ● | ● | ● | ● | ● |
|  | LW50 | ● | ● | ● | ● | ● | ● | ● | ● | ● | ● | ● | ● | ● | ● | ● | ● | ● | ● | ● | ● | ● | ● | ● | ● | ● | ● | ● | ● | ● | ● | ● | ● | ● | ● | ● | ● | ● | ● | ● | ● | ● | ● | ● | ● | ● | ● | ● | ● | ● | ● | ● | ● | ● | ● | ● | ● | ● | ● | ● | ● |
|  | LW51 | ● | ● | ● | ● | ● | ● | ● | ● | ● | ● | ● | ● | ● | ● | ● | ● | ● | ● | ● | ● | ● | ● | ● | ● | ● | ● | ● | ● | ● | ● | ● | ● | ● | ● | ● | ● | ● | ● | ● | ● | ● | ● | ● | ● | ● | ● | ● | ● | ● | ● | ● | ● | ● | ● | ● | ● | ● | ● | ● | ● |
|  | LW59 | ● | ● | ● | ● | ● | ● | ● | ● | ● | ● | ● | ● | ● | ● | ● | ● | ● | ● | ● | ● | ● | ● | ● | ● | ● | ● | ● | ● | ● | ● | ● | ● | ● | ● | ● | ● | ● | ● | ● | ● | ● | ● | ● | ● | ● | ● | ● | ● | ● | ● | ● | ● | ● | ● | ● | ● | ● | ● | ● | ● |
|  | LW67 | ● | ● | ● | ● | ● | ● | ● | ● | ● | ● | ● | ● | ● | ● | ● | ● | ● | ● | ● | ● | ● | ● | ● | ● | ● | ● | ● | ● | ● | ● | ● | ● | ● | ● | ● | ● | ● | ● | ● | ● | ● | ● | ● | ● | ● | ● | ● | ● | ● | ● | ● | ● | ● | ● | ● | ● | ● | ● | ● | ● |
|  | LW74 | ● | ● | ● | ● | ● | ● | ● | ● | ● | ● | ● | ● | ● | ● | ● | ● | ● | ● | ● | ● | ● | ● | ● | ● | ● | ● | ● | ● | ● | ● | ● | ● | ● | ● | ● | ● | ● | ● | ● | ● | ● | ● | ● | ● | ● | ● | ● | ● | ● | ● | ● | ● | ● | ● | ● | ● | ● | ● | ● | ● |
| Bai | DQ897952 | T | ● | ● | ● | ● | ● | ● | ● | ● | ● | ● | ● | ● | ● | ● | ● | ● | ● | ● | ● | ● | ● | ● | ● | ● | ● | ● | ● | ● | ● | ● | ● | ● | ● | ● | ● | ● | ● | ● | ● | ● | ● | ● | ● | ● | ● | T | ● | A | ● | ● | ● | ● | ● | ● | ● | ● | ● | ● | ● |
|  | DQ897953 | T | ● | ● | ● | ● | ● | ● | ● | ● | ● | ● | ● | ● | ● | ● | ● | ● | ● | ● | ● | ● | ● | ● | ● | ● | ● | ● | ● | ● | ● | ● | ● | ● | ● | ● | ● | ● | ● | ● | ● | ● | ● | ● | ● | ● | ● | T | ● | A | ● | ● | ● | ● | ● | ● | ● | ● | ● | ● | ● |
|  | DQ897954 | T | ● | ● | ● | ● | ● | ● | ● | ● | ● | ● | ● | ● | ● | ● | ● | ● | ● | ● | ● | ● | ● | ● | ● | ● | ● | ● | ● | ● | ● | ● | ● | ● | ● | ● | ● | ● | ● | ● | ● | ● | ● | ● | ● | ● | ● | T | ● | A | ● | ● | ● | ● | ● | ● | ● | ● | ● | ● | ● |
|  | DQ897955 | T | ● | ● | ● | ● | ● | ● | ● | ● | ● | ● | ● | ● | ● | ● | ● | ● | ● | ● | ● | ● | ● | ● | ● | ● | ● | ● | ● | ● | ● | ● | ● | ● | ● | ● | ● | ● | ● | ● | ● | ● | ● | ● | ● | ● | ● | T | ● | A | ● | ● | ● | ● | ● | ● | ● | ● | ● | ● | ● |
| Cra | DQ897947 | T | ● | ● | ● | ● | ● | ● | ● | ● | ● | ● | ● | ● | ● | ● | ● | ● | ● | ● | ● | ● | ● | ● | ● | ● | ● | ● | ● | ● | ● | ● | ● | ● | ● | ● | ● | ● | ● | ● | ● | ● | ● | ● | ● | ● | ● | T | ● | A | ● | ● | C | ● | ● | ● | ● | ● | ● | ● | ● |
|  | DQ897948 | T | ● | ● | ● | ● | ● | ● | ● | ● | ● | ● | ● | ● | ● | ● | ● | ● | ● | ● | ● | ● | ● | ● | ● | ● | ● | ● | ● | ● | ● | ● | ● | ● | ● | ● | ● | ● | ● | ● | ● | ● | ● | ● | ● | ● | ● | T | ● | A | ● | ● | C | ● | ● | ● | ● | ● | ● | ● | ● |
| Dir | AB518499 | T | ● | ● | ● | ● | ● | ● | ● | ● | ● | ● | ● | ● | ● | ● | ● | ● | ● | ● | ● | ● | ● | ● | ● | ● | ● | ● | ● | ● | ● | ● | ● | ● | ● | ● | ● | ● | ● | ● | ● | ● | ● | ● | ● | ● | ● | ● | ● | A | ● | ● | ● | ● | ● | ● | ● | ● | ● | ● | ● |
|  | AB518500 | T | ● | ● | ● | ● | ● | ● | ● | ● | ● | ● | ● | ● | ● | ● | ● | ● | ● | ● | ● | ● | ● | ● | ● | ● | ● | ● | ● | ● | ● | ● | ● | ● | ● | ● | ● | ● | ● | ● | ● | ● | ● | ● | ● | ● | ● | T | ● | A | ● | ● | ● | ● | ● | ● | ● | ● | ● | ● | ● |
|  | AB518501 | T | ● | ● | ● | ● | ● | ● | ● | ● | ● | ● | ● | ● | ● | ● | ● | ● | ● | ● | ● | ● | ● | ● | ● | ● | ● | ● | ● | ● | ● | ● | ● | ● | ● | ● | ● | ● | ● | ● | ● | ● | ● | ● | ● | ● | ● | T | ● | A | ● | ● | ● | ● | ● | ● | ● | ● | ● | ● | ● |
|  | AB518502 | T | ● | ● | ● | ● | ● | ● | ● | ● | ● | ● | ● | ● | ● | ● | ● | ● | ● | ● | ● | ● | ● | ● | ● | ● | ● | ● | ● | ● | ● | ● | ● | ● | ● | ● | ● | ● | ● | ● | ● | ● | ● | ● | ● | ● | ● | T | ● | A | ● | ● | ● | ● | ● | ● | ● | ● | ● | ● | ● |
|  | AB518503 | T | ● | ● | ● | ● | ● | ● | ● | ● | ● | ● | ● | ● | ● | ● | ● | ● | ● | ● | ● | ● | ● | ● | ● | ● | ● | ● | ● | ● | ● | ● | ● | ● | ● | ● | ● | ● | ● | ● | ● | ● | ● | ● | ● | ● | ● | T | ● | A | ● | ● | ● | ● | ● | ● | ● | ● | ● | ● | ● |
|  | AB518504 | T | ● | ● | ● | ● | ● | ● | ● | ● | ● | ● | ● | ● | ● | ● | ● | ● | ● | ● | ● | ● | ● | ● | ● | ● | ● | ● | ● | ● | ● | ● | ● | ● | ● | ● | ● | ● | ● | ● | ● | ● | ● | ● | ● | ● | ● | T | ● | A | ● | ● | ● | ● | ● | ● | ● | ● | ● | ● | ● |
|  | AB518505 | T | ● | ● | ● | ● | ● | ● | ● | ● | ● | ● | ● | ● | ● | ● | ● | ● | ● | ● | ● | ● | ● | ● | ● | ● | ● | ● | ● | ● | ● | ● | ● | ● | ● | ● | ● | ● | ● | ● | ● | ● | ● | ● | ● | ● | ● | T | ● | A | ● | ● | ● | ● | ● | ● | ● | ● | ● | ● | ● |
|  | AB518506 | T | ● | ● | ● | ● | ● | ● | ● | ● | ● | ● | ● | ● | ● | ● | ● | ● | ● | ● | ● | ● | ● | ● | ● | ● | ● | ● | ● | ● | ● | ● | ● | ● | ● | ● | ● | ● | ● | ● | ● | ● | ● | ● | ● | ● | ● | T | ● | A | ● | ● | ● | ● | ● | ● | ● | ● | ● | ● | ● |
|  | AB518507 | T | ● | ● | ● | ● | ● | ● | ● | ● | ● | ● | ● | ● | ● | ● | ● | ● | ● | ● | ● | ● | ● | ● | ● | ● | ● | ● | ● | ● | ● | ● | ● | ● | ● | ● | ● | ● | ● | ● | ● | ● | ● | ● | ● | ● | ● | T | ● | A | ● | ● | ● | ● | ● | ● | ● | ● | ● | ● | ● |
|  | AB518508 | T | ● | ● | ● | ● | ● | ● | ● | ● | ● | ● | ● | ● | ● | ● | ● | ● | ● | ● | ● | ● | ● | ● | ● | ● | ● | ● | ● | ● | ● | ● | ● | ● | ● | ● | ● | ● | ● | ● | ● | ● | ● | ● | ● | ● | ● | T | ● | A | ● | ● | ● | ● | ● | ● | ● | ● | ● | ● | ● |
|  | AB518509 | T | ● | ● | ● | ● | ● | ● | ● | ● | ● | ● | ● | ● | ● | ● | ● | ● | ● | ● | ● | ● | ● | ● | ● | ● | ● | ● | ● | ● | ● | ● | ● | ● | ● | ● | ● | ● | ● | ● | ● | ● | ● | ● | ● | ● | ● | T | ● | A | ● | ● | ● | ● | ● | ● | ● | ● | ● | ● | ● |
|  | AB518510 | T | ● | ● | ● | ● | ● | ● | ● | ● | ● | ● | ● | ● | ● | ● | ● | ● | ● | ● | ● | ● | ● | ● | ● | ● | ● | ● | ● | ● | ● | ● | ● | ● | ● | ● | ● | ● | ● | ● | ● | ● | ● | ● | ● | ● | ● | T | ● | A | ● | ● | ● | ● | ● | ● | ● | ● | ● | ● | ● |
|  | AB518511 | T | ● | ● | ● | ● | ● | ● | ● | ● | ● | ● | ● | ● | ● | ● | ● | ● | ● | ● | ● | ● | ● | ● | ● | ● | ● | ● | ● | ● | ● | ● | ● | ● | ● | ● | ● | ● | ● | ● | ● | ● | ● | ● | ● | ● | ● | T | ● | A | ● | ● | ● | ● | ● | ● | ● | ● | ● | ● | ● |
|  | AB518512 | T | ● | ● | ● | ● | ● | ● | ● | ● | ● | ● | ● | ● | ● | ● | ● | ● | ● | ● | ● | ● | ● | ● | ● | ● | ● | ● | ● | ● | ● | ● | ● | ● | ● | ● | ● | ● | ● | ● | ● | ● | ● | ● | ● | ● | ● | T | ● | A | ● | ● | ● | ● | ● | ● | ● | ● | ● | ● | ● |
|  | AB518513 | T | ● | ● | ● | ● | ● | ● | ● | ● | ● | ● | ● | ● | ● | ● | ● | ● | ● | ● | ● | ● | ● | ● | ● | ● | ● | ● | ● | ● | ● | ● | ● | ● | ● | ● | ● | ● | ● | ● | ● | ● | ● | ● | ● | ● | ● | T | ● | A | ● | ● | ● | ● | ● | ● | ● | ● | ● | ● | ● |
|  | AB518514 | T | ● | ● | ● | ● | ● | ● | ● | ● | ● | ● | ● | ● | ● | ● | ● | ● | ● | ● | ● | ● | ● | ● | ● | ● | ● | ● | ● | ● | ● | ● | ● | ● | ● | ● | ● | ● | ● | ● | ● | ● | ● | ● | ● | ● | ● | T | ● | A | ● | ● | ● | ● | ● | ● | ● | ● | ● | ● | ● |
|  | AB518515 | T | ● | ● | ● | ● | ● | ● | ● | ● | ● | ● | ● | ● | ● | ● | ● | ● | ● | ● | ● | ● | ● | ● | ● | ● | ● | ● | ● | ● | ● | ● | ● | ● | ● | ● | ● | ● | ● | ● | ● | ● | ● | ● | ● | ● | ● | T | ● | A | ● | ● | ● | ● | ● | ● | ● | ● | ● | ● | ● |
|  | AB518516 | T | ● | ● | ● | ● | ● | ● | ● | ● | ● | ● | ● | ● | ● | ● | ● | ● | ● | ● | ● | ● | ● | ● | ● | ● | ● | ● | ● | ● | ● | ● | ● | ● | ● | ● | ● | ● | ● | ● | ● | ● | ● | ● | ● | ● | ● | T | ● | A | ● | ● | ● | ● | ● | ● | ● | ● | ● | ● | ● |
|  | AB518517 | T | ● | ● | ● | ● | ● | ● | ● | ● | ● | ● | ● | ● | ● | ● | ● | ● | ● | ● | ● | ● | ● | ● | ● | ● | ● | ● | ● | ● | ● | ● | ● | ● | ● | ● | ● | ● | ● | ● | ● | ● | ● | ● | ● | ● | ● | T | ● | A | ● | ● | ● | ● | ● | ● | ● | ● | ● | ● | ● |
|  | AB518518 | T | ● | ● | ● | ● | ● | ● | ● | ● | ● | ● | ● | ● | ● | ● | ● | ● | ● | ● | ● | ● | ● | ● | ● | ● | ● | ● | ● | ● | ● | ● | ● | ● | ● | ● | ● | ● | ● | ● | ● | ● | ● | ● | ● | ● | ● | T | ● | A | ● | ● | ● | ● | ● | ● | ● | ● | ● | ● | ● |
|  | AB518519 | T | ● | ● | ● | ● | ● | ● | ● | ● | ● | ● | ● | ● | ● | ● | ● | ● | ● | ● | ● | ● | ● | ● | ● | ● | ● | ● | ● | ● | ● | ● | ● | ● | ● | ● | ● | ● | ● | ● | ● | ● | ● | ● | ● | ● | ● | T | ● | A | ● | ● | ● | ● | ● | ● | ● | ● | ● | ● | ● |
|  | AB518520 | T | ● | ● | ● | ● | ● | ● | ● | ● | ● | ● | ● | ● | ● | ● | ● | ● | ● | ● | ● | ● | ● | ● | ● | ● | ● | ● | ● | ● | ● | ● | ● | ● | ● | ● | ● | ● | ● | ● | ● | ● | ● | ● | ● | ● | ● | T | ● | A | ● | ● | ● | ● | ● | ● | ● | ● | ● | ● | ● |
|  | AB518521 | T | ● | ● | ● | ● | ● | ● | ● | ● | ● | ● | ● | ● | ● | ● | ● | ● | ● | ● | ● | ● | ● | ● | ● | ● | ● | ● | C | ● | ● | ● | ● | ● | ● | ● | ● | ● | ● | ● | ● | ● | ● | ● | ● | ● | ● | T | ● | A | ● | ● | ● | ● | ● | G | ● | ● | ● | ● | ● |
|  | AB518522 | T | ● | ● | ● | ● | ● | ● | ● | ● | ● | ● | ● | ● | ● | ● | ● | ● | ● | ● | ● | ● | ● | ● | ● | ● | ● | ● | ● | ● | ● | ● | ● | ● | ● | ● | ● | ● | ● | ● | ● | ● | ● | ● | ● | ● | ● | T | ● | A | ● | ● | ● | ● | ● | ● | ● | ● | ● | ● | ● |
|  | AB518523 | T | ● | ● | ● | ● | ● | ● | ● | ● | ● | ● | ● | ● | ● | ● | ● | ● | ● | ● | ● | ● | ● | ● | ● | ● | ● | ● | ● | ● | ● | ● | ● | ● | ● | ● | ● | ● | ● | ● | ● | ● | ● | ● | ● | ● | ● | T | ● | A | ● | ● | ● | ● | ● | ● | ● | ● | ● | ● | ● |
|  | AB518524 | T | ● | ● | ● | ● | ● | ● | ● | ● | ● | ● | ● | ● | ● | ● | ● | ● | ● | ● | ● | ● | ● | ● | ● | ● | ● | ● | ● | ● | ● | ● | ● | ● | ● | ● | ● | ● | ● | ● | ● | ● | ● | ● | ● | ● | ● | T | ● | A | ● | ● | ● | ● | ● | ● | ● | ● | ● | ● | ● |
|  | AB518525 | T | ● | ● | ● | ● | ● | ● | ● | ● | ● | ● | ● | ● | ● | ● | ● | ● | ● | ● | ● | ● | ● | ● | ● | ● | ● | ● | ● | ● | ● | ● | ● | ● | ● | ● | ● | ● | ● | ● | ● | ● | ● | ● | ● | ● | ● | T | ● | A | ● | ● | ● | ● | ● | ● | ● | ● | ● | ● | ● |
|  | AB518526 | T | ● | ● | ● | ● | ● | ● | ● | ● | ● | ● | ● | ● | ● | ● | ● | ● | ● | ● | ● | ● | ● | ● | ● | ● | ● | ● | ● | ● | ● | ● | ● | ● | ● | ● | ● | ● | ● | ● | ● | ● | ● | ● | ● | ● | ● | T | ● | A | ● | ● | ● | ● | ● | ● | ● | ● | ● | ● | ● |
|  | AB518527 | T | ● | ● | ● | ● | ● | ● | ● | ● | ● | ● | ● | ● | ● | ● | ● | ● | ● | ● | ● | ● | ● | ● | ● | ● | ● | ● | ● | ● | ● | ● | ● | ● | ● | ● | ● | ● | ● | ● | ● | ● | ● | ● | ● | ● | ● | T | ● | A | ● | ● | ● | ● | ● | ● | ● | ● | ● | ● | ● |
|  | AB518528 | T | ● | ● | ● | ● | ● | ● | ● | ● | ● | ● | ● | ● | ● | ● | ● | ● | ● | ● | ● | ● | ● | ● | ● | ● | ● | ● | ● | ● | ● | ● | ● | ● | ● | ● | ● | ● | ● | ● | ● | ● | ● | ● | ● | ● | ● | T | ● | A | ● | ● | ● | ● | ● | ● | ● | ● | ● | ● | ● |
|  | AB518529 | T | ● | ● | ● | ● | ● | ● | ● | ● | ● | ● | ● | ● | ● | ● | ● | ● | ● | ● | ● | ● | ● | ● | ● | ● | ● | ● | ● | ● | ● | ● | ● | ● | ● | ● | ● | ● | ● | ● | ● | ● | ● | ● | ● | ● | ● | T | ● | A | ● | ● | ● | ● | ● | ● | ● | ● | ● | ● | ● |
|  | AB518530 | T | ● | ● | ● | ● | ● | ● | ● | ● | ● | ● | ● | ● | ● | ● | ● | ● | ● | ● | ● | ● | ● | ● | ● | ● | ● | ● | ● | ● | ● | ● | ● | ● | ● | ● | ● | ● | ● | ● | ● | ● | ● | ● | ● | ● | ● | T | ● | A | ● | ● | ● | ● | ● | ● | ● | ● | ● | ● | ● |
|  | AB518531 | T | ● | ● | ● | ● | ● | ● | ● | ● | ● | ● | ● | ● | ● | ● | ● | ● | ● | ● | ● | ● | ● | ● | ● | ● | ● | ● | ● | ● | ● | ● | ● | ● | ● | ● | ● | ● | ● | ● | ● | ● | ● | ● | ● | ● | ● | T | ● | A | ● | ● | ● | ● | ● | ● | ● | ● | ● | ● | ● |
|  | AB518532 | T | ● | ● | ● | ● | ● | ● | ● | ● | ● | ● | ● | ● | ● | ● | ● | ● | ● | ● | ● | ● | ● | ● | ● | ● | ● | ● | ● | ● | ● | ● | ● | ● | ● | ● | ● | ● | ● | ● | ● | ● | ● | ● | ● | ● | ● | T | ● | A | ● | ● | ● | ● | ● | ● | ● | ● | ● | ● | ● |
|  | AB518533 | T | ● | ● | ● | ● | ● | ● | ● | ● | ● | ● | ● | ● | ● | ● | ● | ● | ● | ● | ● | ● | ● | ● | ● | ● | ● | ● | ● | ● | ● | ● | ● | ● | ● | ● | ● | ● | ● | ● | ● | ● | ● | ● | ● | ● | ● | T | ● | A | ● | ● | ● | ● | ● | ● | ● | ● | ● | ● | ● |
|  | DQ897944 | T | ● | ● | ● | ● | ● | ● | ● | ● | ● | ● | ● | ● | ● | ● | ● | ● | ● | ● | ● | ● | ● | ● | ● | ● | ● | ● | ● | ● | ● | ● | ● | ● | ● | ● | ● | ● | ● | ● | ● | ● | ● | ● | ● | ● | ● | T | ● | A | ● | ● | ● | ● | ● | ● | ● | ● | ● | ● | ● |
|  | DQ897945 | T | ● | ● | ● | ● | ● | ● | ● | ● | ● | ● | ● | ● | ● | ● | ● | ● | ● | ● | ● | ● | ● | ● | ● | ● | ● | ● | ● | ● | ● | ● | ● | ● | ● | ● | ● | ● | ● | ● | ● | ● | ● | ● | ● | ● | ● | T | ● | A | ● | ● | ● | ● | ● | ● | ● | ● | ● | ● | ● |
|  | DQ897946 | T | ● | ● | ● | ● | ● | ● | ● | ● | ● | ● | ● | ● | ● | ● | ● | ● | ● | ● | ● | ● | ● | ● | ● | ● | ● | ● | ● | ● | ● | ● | ● | ● | ● | ● | ● | ● | ● | ● | ● | ● | ● | ● | ● | ● | ● | T | ● | A | ● | ● | ● | ● | ● | ● | ● | ● | ● | ● | ● |
| Ele | DQ897957 | T | ● | ● | ● | ● | ● | ● | ● | ● | ● | ● | ● | ● | ● | ● | ● | ● | ● | ● | ● | ● | ● | ● | ● | ● | ● | ● | ● | ● | ● | C | ● | ● | ● | ● | ● | ● | ● | ● | ● | ● | ● | ● | ● | ● | ● | T | ● | A | ● | ● | ● | ● | ● | ● | ● | ● | ● | ● | ● |
|  | DQ897958 | T | ● | ● | ● | ● | ● | ● | ● | ● | ● | ● | ● | ● | ● | ● | ● | ● | ● | ● | ● | ● | ● | ● | ● | ● | ● | ● | ● | ● | ● | C | ● | ● | ● | ● | ● | ● | ● | ● | ● | ● | ● | ● | ● | ● | ● | T | ● | A | ● | ● | ● | ● | ● | ● | ● | ● | ● | ● | ● |
| Gam | L20934 | T | ● | ● | ● | ● | ● | ● | ● | ● | ● | ● | ● | ● | ● | ● | C | ● | ● | ● | ● | ● | ● | ● | ● | ● | ● | ● | ● | ● | ● | A | ● | ● | ● | ● | ● | C | ● | ● | ● | ● | ● | ● | ● | ● | ● | T | ● | A | ● | ● | A | ● | ● | ● | ● | ● | A | ● | ● |
| Int | KM032605 | T | ● | ● | ● | ● | ● | ● | ● | ● | ● | ● | ● | ● | ● | ● | ● | ● | ● | ● | ● | ● | ● | ● | ● | ● | ● | ● | ● | ● | ● | ● | ● | ● | ● | ● | ● | ● | ● | ● | ● | ● | ● | ● | ● | ● | G | T | ● | A | ● | ● | ● | ● | ● | ● | ● | ● | ● | ● | ● |
|  | KM032606 | T | ● | ● | ● | ● | ● | ● | ● | ● | ● | ● | ● | ● | ● | ● | ● | ● | ● | ● | ● | ● | ● | ● | ● | ● | ● | ● | ● | ● | ● | ● | ● | ● | ● | ● | ● | ● | ● | ● | ● | ● | ● | ● | ● | ● | G | T | ● | A | ● | ● | ● | ● | ● | ● | ● | ● | ● | ● | ● |
|  | KM032607 | T | ● | ● | ● | ● | ● | ● | ● | ● | ● | ● | ● | ● | ● | ● | ● | ● | ● | ● | ● | ● | ● | ● | ● | ● | ● | ● | ● | ● | ● | ● | ● | ● | ● | ● | ● | ● | ● | ● | ● | ● | ● | ● | ● | ● | G | T | ● | A | ● | ● | ● | ● | ● | ● | ● | ● | ● | ● | ● |
|  | KM032608 | T | ● | ● | ● | ● | ● | ● | ● | ● | ● | ● | ● | ● | ● | ● | ● | ● | ● | ● | ● | ● | ● | ● | ● | ● | ● | ● | ● | ● | ● | ● | ● | ● | ● | ● | ● | ● | ● | ● | ● | ● | ● | ● | ● | ● | G | T | ● | A | ● | ● | ● | ● | ● | ● | ● | ● | ● | ● | ● |
|  | KM032609 | T | ● | ● | ● | ● | ● | ● | ● | ● | ● | ● | ● | ● | ● | ● | ● | ● | ● | ● | ● | ● | ● | ● | ● | ● | ● | ● | ● | ● | ● | ● | ● | ● | ● | ● | ● | ● | ● | ● | ● | ● | ● | ● | ● | ● | G | T | ● | A | ● | ● | ● | ● | ● | ● | ● | ● | ● | ● | ● |
|  | KM032610 | T | ● | ● | ● | ● | ● | ● | ● | ● | ● | ● | ● | ● | ● | ● | ● | ● | ● | ● | ● | ● | ● | ● | ● | ● | ● | ● | ● | ● | ● | ● | ● | ● | ● | ● | ● | ● | ● | ● | ● | ● | ● | ● | ● | ● | G | T | ● | A | ● | ● | ● | ● | ● | ● | ● | ● | ● | ● | ● |
|  | KM032611 | T | ● | ● | ● | ● | ● | ● | ● | ● | ● | ● | ● | ● | ● | ● | ● | ● | ● | ● | ● | ● | ● | ● | ● | ● | ● | ● | ● | ● | ● | ● | ● | ● | ● | ● | ● | ● | ● | ● | ● | ● | ● | ● | ● | ● | G | T | ● | A | ● | ● | ● | ● | ● | ● | ● | ● | ● | ● | ● |
|  | KM032612 | T | ● | ● | ● | ● | ● | ● | ● | ● | ● | ● | ● | ● | ● | ● | ● | ● | ● | ● | ● | ● | ● | ● | ● | ● | ● | ● | ● | ● | ● | ● | ● | ● | ● | ● | ● | ● | ● | ● | ● | ● | ● | ● | ● | ● | G | T | ● | A | ● | ● | ● | ● | ● | ● | ● | ● | ● | ● | ● |
| Lat | DQ897936 | T | ● | ● | ● | ● | ● | ● | ● | ● | ● | ● | ● | ● | ● | ● | ● | ● | ● | ● | ● | ● | ● | ● | ● | ● | ● | ● | ● | ● | ● | A | ● | ● | ● | ● | ● | C | ● | ● | ● | ● | ● | ● | ● | ● | ● | T | ● | A | ● | ● | ● | ● | ● | ● | ● | ● | A | ● | ● |
|  | DQ897937 | T | ● | ● | ● | ● | ● | ● | ● | ● | ● | ● | ● | ● | ● | ● | ● | ● | ● | ● | ● | ● | ● | ● | ● | T | ● | ● | C | ● | ● | A | ● | ● | ● | ● | ● | C | ● | ● | ● | ● | ● | ● | ● | ● | ● | T | ● | A | ● | ● | ● | ● | ● | ● | ● | ● | A | ● | ● |
| Leu | DQ897939 | T | ● | ● | ● | ● | ● | ● | ● | ● | ● | ● | ● | ● | ● | ● | ● | ● | ● | ● | ● | ● | ● | ● | ● | ● | ● | ● | ● | ● | ● | G | ● | ● | ● | ● | ● | C | ● | ● | ● | ● | ● | ● | ● | ● | ● | T | ● | A | ● | ● | ● | ● | ● | ● | ● | ● | A | ● | ● |
| Mac | DQ897969 | T | ● | ● | ● | ● | ● | ● | ● | ● | ● | ● | ● | ● | ● | ● | ● | ● | ● | ● | ● | ● | ● | ● | ● | ● | ● | ● | ● | ● | ● | ● | ● | ● | A | ● | ● | ● | ● | ● | ● | ● | ● | ● | ● | ● | ● | ● | ● | ● | ● | ● | ● | ● | ● | ● | ● | ● | ● | ● | ● |
|  | DQ897970 | T | ● | ● | ● | ● | ● | ● | ● | ● | ● | ● | ● | ● | ● | ● | ● | ● | ● | ● | ● | ● | ● | ● | ● | ● | ● | ● | ● | ● | ● | ● | ● | ● | A | ● | ● | ● | ● | ● | ● | ● | ● | ● | ● | ● | ● | ● | ● | ● | ● | ● | ● | ● | ● | ● | ● | ● | ● | ● | ● |
|  | DQ897971 | T | ● | ● | ● | ● | ● | ● | ● | ● | ● | ● | ● | ● | ● | ● | ● | ● | ● | ● | ● | ● | ● | ● | ● | ● | ● | ● | ● | ● | ● | ● | ● | ● | A | ● | ● | ● | ● | ● | ● | ● | ● | ● | ● | ● | ● | ● | ● | ● | ● | ● | ● | ● | ● | ● | ● | ● | ● | ● | ● |
|  | DQ897972 | T | ● | ● | ● | ● | ● | ● | ● | ● | ● | ● | ● | ● | ● | ● | ● | ● | ● | ● | ● | ● | ● | ● | ● | ● | ● | ● | ● | ● | ● | ● | ● | ● | A | ● | ● | ● | ● | ● | ● | ● | ● | ● | ● | ● | ● | ● | ● | ● | ● | ● | ● | ● | ● | ● | ● | ● | ● | ● | ● |
| Mir | DQ897965 | T | ● | ● | ● | ● | ● | ● | ● | ● | ● | ● | ● | ● | ● | ● | ● | ● | ● | ● | ● | ● | ● | ● | ● | ● | ● | ● | C | ● | ● | ● | ● | ● | ● | ● | ● | ● | ● | ● | A | ● | ● | ● | ● | ● | ● | ● | ● | ● | ● | ● | ● | ● | ● | ● | ● | ● | ● | ● | ● |
|  | DQ897966 | T | ● | ● | ● | ● | ● | ● | ● | ● | ● | ● | ● | ● | ● | ● | ● | ● | ● | ● | ● | ● | ● | ● | ● | ● | ● | ● | C | ● | ● | ● | ● | ● | ● | ● | ● | ● | ● | ● | A | ● | ● | ● | ● | ● | ● | ● | ● | ● | ● | ● | ● | ● | ● | ● | ● | ● | ● | ● | ● |
| Nem | DQ897959 | T | ● | ● | ● | ● | ● | ● | ● | ● | ● | ● | ● | ● | ● | ● | ● | ● | ● | ● | ● | ● | ● | ● | ● | ● | ● | ● | ● | ● | ● | ● | ● | ● | ● | ● | ● | ● | ● | ● | ● | ● | ● | ● | ● | ● | ● | T | ● | A | ● | ● | ● | ● | ● | ● | ● | ● | ● | ● | ● |
|  | DQ897960 | T | ● | ● | ● | ● | ● | ● | ● | ● | ● | ● | ● | ● | ● | ● | ● | ● | ● | ● | ● | ● | ● | ● | ● | ● | ● | ● | ● | ● | ● | ● | ● | ● | ● | ● | ● | ● | ● | ● | ● | ● | ● | ● | ● | ● | G | T | ● | A | ● | ● | ● | ● | ● | ● | ● | ● | ● | ● | ● |
|  | DQ897961 | T | ● | ● | ● | ● | ● | ● | ● | ● | ● | ● | ● | ● | ● | ● | ● | ● | ● | ● | ● | ● | ● | ● | ● | ● | ● | ● | C | ● | ● | ● | ● | ● | ● | ● | ● | ● | ● | ● | ● | ● | ● | ● | ● | ● | ● | T | ● | A | ● | ● | ● | ● | ● | ● | ● | ● | ● | ● | ● |
| Sca | DQ897949 | T | ● | ● | ● | ● | ● | ● | ● | ● | ● | ● | ● | ● | ● | ● | ● | ● | ● | ● | ● | ● | ● | ● | ● | ● | ● | ● | ● | ● | ● | ● | ● | ● | ● | ● | ● | ● | ● | ● | ● | ● | ● | ● | ● | ● | ● | T | ● | A | ● | ● | ● | ● | ● | ● | ● | ● | ● | ● | ● |
| Tak cf | AB518534 | T | ● | ● | ● | ● | ● | ● | ● | ● | ● | ● | ● | ● | ● | ● | ● | ● | ● | ● | ● | ● | ● | ● | ● | ● | ● | ● | ● | ● | ● | ● | ● | ● | ● | ● | ● | ● | ● | ● | ● | ● | ● | ● | ● | ● | ● | ● | ● | A | ● | ● | ● | ● | ● | ● | ● | ● | ● | ● | ● |
|  | AB518535 | T | ● | ● | ● | ● | ● | ● | ● | ● | ● | ● | ● | ● | ● | ● | ● | ● | ● | ● | ● | ● | ● | ● | ● | ● | ● | ● | ● | ● | ● | ● | ● | ● | ● | ● | ● | ● | ● | ● | ● | ● | ● | ● | ● | ● | ● | ● | ● | A | ● | ● | ● | ● | ● | ● | ● | ● | ● | ● | ● |
|  | AB518536 | T | ● | ● | ● | ● | ● | ● | ● | ● | ● | ● | ● | ● | ● | ● | ● | ● | ● | ● | ● | ● | ● | ● | ● | ● | ● | ● | ● | ● | ● | ● | ● | ● | ● | ● | ● | ● | ● | ● | ● | ● | ● | ● | ● | ● | ● | ● | ● | A | ● | ● | ● | ● | ● | ● | ● | ● | ● | ● | ● |
|  | AB518537 | T | ● | ● | ● | ● | ● | ● | ● | ● | ● | ● | ● | ● | ● | ● | ● | ● | ● | ● | ● | ● | ● | ● | ● | ● | ● | ● | ● | ● | ● | ● | ● | ● | ● | ● | ● | ● | ● | ● | ● | ● | ● | ● | ● | ● | ● | ● | ● | A | ● | ● | ● | ● | ● | ● | ● | ● | ● | ● | ● |
|  | AB518538 | T | ● | ● | ● | ● | ● | ● | ● | ● | ● | ● | ● | ● | ● | ● | ● | ● | ● | ● | ● | ● | ● | ● | ● | ● | ● | ● | ● | ● | ● | ● | ● | ● | ● | ● | ● | ● | ● | ● | ● | ● | ● | ● | ● | ● | ● | ● | ● | A | ● | ● | ● | ● | ● | ● | ● | ● | ● | ● | ● |
| Tak | DQ897962 | ● | ● | ● | ● | ● | ● | ● | ● | ● | ● | ● | ● | ● | ● | ● | ● | ● | ● | ● | ● | ● | ● | ● | ● | ● | ● | ● | ● | ● | ● | ● | ● | ● | ● | ● | ● | ● | ● | ● | ● | ● | ● | ● | ● | ● | ● | T | ● | A | ● | ● | ● | ● | ● | ● | ● | ● | ● | ● | ● |
|  | DQ897963 | ● | ● | ● | ● | ● | ● | ● | ● | ● | ● | ● | ● | ● | ● | ● | ● | ● | ● | ● | ● | ● | ● | ● | ● | ● | ● | ● | ● | ● | ● | ● | ● | ● | ● | ● | ● | ● | ● | ● | ● | ● | ● | ● | ● | ● | ● | T | ● | A | ● | ● | ● | ● | ● | ● | ● | ● | ● | ● | ● |
|  | DQ897964 | ● | ● | ● | ● | ● | ● | ● | ● | ● | ● | ● | ● | ● | ● | G | ● | ● | ● | ● | ● | ● | ● | ● | ● | ● | ● | ● | ● | ● | ● | ● | ● | ● | ● | ● | ● | ● | ● | ● | ● | ● | ● | ● | ● | ● | ● | T | ● | A | ● | ● | ● | ● | ● | ● | ● | ● | ● | ● | ● |

Bal = *An. balabacensi*s, Bai = *An. baimaii*, Cra = *An. cracens*, Dir = *An. dirus*, Ele = *An. elegans*, Gam = *An. gambiae*, Int = *An. introlatus*, Lat = *An. latens*, Leu = *An. leucosphyrus*, Mac = *An. macarthuri*, Mir = *An. mirans*, Nem = *An. nemophilous*, Sca = *An. scanloni*, Tak = *An. takasagoensis*, cf = confer

**● = nucleotide identical to sequence DQ897940

| Species* | Accession/  Sample  ID | Position of SNP (bp)** | | | | | | | | | | | | | | | | | | | | | | | | | | | | | | | | | | | | | | | | | | | | | | | | | | | | | | | | | | | |
| --- | --- | --- | --- | --- | --- | --- | --- | --- | --- | --- | --- | --- | --- | --- | --- | --- | --- | --- | --- | --- | --- | --- | --- | --- | --- | --- | --- | --- | --- | --- | --- | --- | --- | --- | --- | --- | --- | --- | --- | --- | --- | --- | --- | --- | --- | --- | --- | --- | --- | --- | --- | --- | --- | --- | --- | --- | --- | --- | --- | --- | --- |
|  |  | 1 | 1 | 1 | 1 | 1 | 1 | 1 | 1 | 1 | 1 | 1 | 1 | 1 | 1 | 1 | 1 | 1 | 1 | 1 | 2 | 2 | 2 | 2 | 2 | 2 | 2 | 2 | 2 | 2 | 2 | 2 | 2 | 2 | 2 | 2 | 2 | 2 | 2 | 2 | 2 | 2 |  |  |  |  |  |  |  |  |  |  |  |  |  |  |  |  |  |  |  |
|  |  | 8 | 8 | 8 | 8 | 8 | 8 | 8 | 8 | 8 | 9 | 9 | 9 | 9 | 9 | 9 | 9 | 9 | 9 | 9 | 0 | 0 | 0 | 0 | 0 | 0 | 0 | 0 | 0 | 0 | 1 | 1 | 1 | 1 | 1 | 1 | 1 | 1 | 1 | 1 | 2 | 2 |  |  |  |  |  |  |  |  |  |  |  |  |  |  |  |  |  |  |  |
|  |  | 1 | 2 | 3 | 4 | 5 | 6 | 7 | 8 | 9 | 0 | 1 | 2 | 3 | 4 | 5 | 6 | 7 | 8 | 9 | 0 | 1 | 2 | 3 | 4 | 5 | 6 | 7 | 8 | 9 | 0 | 1 | 2 | 3 | 4 | 5 | 6 | 7 | 8 | 9 | 0 | 1 |  |  |  |  |  |  |  |  |  |  |  |  |  |  |  |  |  |  |  |
| Bal | DQ897940 | T | G | A | A | T | G | A | T | A | C | C | A | T | A | C | A | T | T | A | C | C | A | C | C | A | G | C | A | G | A | G | C | A | C | A | C | A | T | A | C | G |  |  |  |  |  |  |  |  |  |  |  |  |  |  |  |  |  |  |  |
|  | DQ897941 | ● | ● | ● | ● | ● | ● | ● | ● | ● | T | ● | ● | C | ● | ● | ● | ● | ● | ● | ● | ● | C | ● | ● | ● | ● | ● | ● | ● | ● | A | ● | ● | ● | ● | ● | ● | ● | ● | ● | ● |  |  |  |  |  |  |  |  |  |  |  |  |  |  |  |  |  |  |  |
|  | LW31 | ● | ● | ● | ● | ● | ● | ● | ● | ● | ● | ● | ● | ● | ● | ● | ● | ● | ● | ● | ● | ● | ● | ● | ● | ● | ● | ● | ● | ● | ● | ● | ● | ● | ● | ● | ● | ● | ● | ● | ● | ● |  |  |  |  |  |  |  |  |  |  |  |  |  |  |  |  |  |  |  |
|  | LW32 | ● | ● | ● | ● | ● | ● | ● | ● | ● | ● | ● | ● | ● | ● | ● | ● | ● | ● | ● | ● | ● | ● | ● | ● | ● | ● | ● | ● | ● | ● | ● | ● | ● | ● | ● | ● | ● | ● | ● | ● | ● |  |  |  |  |  |  |  |  |  |  |  |  |  |  |  |  |  |  |  |
|  | LW45 | ● | ● | ● | ● | ● | ● | ● | ● | ● | ● | ● | ● | ● | ● | ● | ● | ● | ● | ● | ● | ● | ● | ● | ● | ● | ● | ● | ● | ● | ● | ● | ● | ● | ● | ● | ● | ● | ● | ● | ● | ● |  |  |  |  |  |  |  |  |  |  |  |  |  |  |  |  |  |  |  |
|  | LW49 | ● | ● | ● | ● | ● | ● | ● | ● | ● | ● | ● | ● | ● | ● | ● | ● | ● | ● | ● | ● | ● | ● | ● | ● | ● | ● | ● | ● | ● | ● | ● | ● | ● | ● | ● | ● | ● | ● | ● | ● | ● |  |  |  |  |  |  |  |  |  |  |  |  |  |  |  |  |  |  |  |
|  | LW50 | ● | ● | ● | ● | ● | ● | ● | ● | ● | ● | ● | ● | ● | ● | ● | ● | ● | ● | ● | ● | ● | ● | ● | ● | ● | ● | ● | ● | ● | ● | ● | ● | ● | ● | ● | ● | ● | ● | ● | ● | ● |  |  |  |  |  |  |  |  |  |  |  |  |  |  |  |  |  |  |  |
|  | LW51 | ● | ● | ● | ● | ● | ● | ● | ● | ● | ● | ● | ● | ● | ● | ● | ● | ● | ● | ● | ● | ● | ● | ● | ● | ● | ● | ● | ● | ● | ● | ● | ● | ● | ● | ● | ● | ● | ● | ● | ● | ● |  |  |  |  |  |  |  |  |  |  |  |  |  |  |  |  |  |  |  |
|  | LW59 | ● | ● | ● | ● | ● | ● | ● | ● | ● | ● | ● | ● | ● | ● | ● | ● | ● | ● | ● | ● | ● | ● | ● | ● | ● | ● | ● | ● | ● | ● | ● | ● | ● | ● | ● | ● | ● | ● | ● | ● | ● |  |  |  |  |  |  |  |  |  |  |  |  |  |  |  |  |  |  |  |
|  | LW67 | ● | ● | ● | ● | ● | ● | ● | ● | ● | ● | ● | ● | ● | ● | ● | ● | ● | ● | ● | ● | ● | ● | ● | ● | ● | ● | ● | ● | ● | ● | ● | ● | ● | ● | ● | ● | ● | ● | ● | ● | ● |  |  |  |  |  |  |  |  |  |  |  |  |  |  |  |  |  |  |  |
|  | LW74 | ● | ● | ● | ● | ● | ● | ● | ● | ● | ● | ● | ● | ● | ● | ● | ● | ● | ● | ● | ● | ● | ● | ● | ● | ● | ● | ● | ● | ● | ● | ● | ● | ● | ● | ● | ● | ● | ● | ● | ● | ● |  |  |  |  |  |  |  |  |  |  |  |  |  |  |  |  |  |  |  |
| Bai | DQ897952 | ● | ● | ● | ● | ● | ● | ● | ● | ● | T | ● | ● | ● | ● | ● | ● | C | ● | ● | ● | ● | T | ● | ● | ● | ● | ● | ● | ● | ● | A | ● | ● | ● | ● | ● | ● | ● | ● | T | ● |  |  |  |  |  |  |  |  |  |  |  |  |  |  |  |  |  |  |  |
|  | DQ897953 | ● | ● | ● | ● | ● | ● | ● | ● | ● | T | ● | ● | ● | ● | ● | ● | C | ● | ● | ● | ● | T | ● | ● | ● | ● | ● | ● | ● | ● | A | ● | ● | ● | ● | ● | ● | ● | ● | T | ● |  |  |  |  |  |  |  |  |  |  |  |  |  |  |  |  |  |  |  |
|  | DQ897954 | ● | ● | ● | ● | ● | ● | ● | ● | ● | T | ● | ● | ● | ● | ● | ● | C | ● | ● | ● | ● | T | ● | ● | ● | ● | ● | ● | ● | ● | A | ● | ● | ● | ● | ● | ● | ● | ● | T | ● |  |  |  |  |  |  |  |  |  |  |  |  |  |  |  |  |  |  |  |
|  | DQ897955 | ● | ● | ● | ● | ● | ● | ● | ● | ● | T | ● | ● | ● | ● | ● | ● | C | ● | ● | ● | ● | T | ● | ● | ● | ● | ● | ● | ● | ● | A | ● | ● | ● | ● | ● | ● | ● | ● | T | ● |  |  |  |  |  |  |  |  |  |  |  |  |  |  |  |  |  |  |  |
| Cra | DQ897947 | ● | ● | ● | G | ● | ● | ● | ● | ● | ● | ● | ● | C | ● | ● | ● | ● | ● | ● | ● | ● | T | ● | ● | ● | ● | ● | ● | ● | ● | A | ● | ● | ● | ● | ● | ● | ● | ● | T | ● |  |  |  |  |  |  |  |  |  |  |  |  |  |  |  |  |  |  |  |
|  | DQ897948 | ● | ● | ● | G | ● | ● | ● | ● | ● | ● | ● | ● | C | ● | ● | ● | ● | ● | ● | ● | ● | T | ● | ● | ● | ● | ● | ● | ● | ● | A | ● | ● | ● | ● | ● | ● | ● | ● | T | ● |  |  |  |  |  |  |  |  |  |  |  |  |  |  |  |  |  |  |  |
| Dir | AB518499 | ● | ● | ● | ● | ● | ● | ● | ● | ● | T | ● | ● | ● | ● | ● | ● | C | ● | ● | ● | ● | T | ● | ● | ● | ● | ● | ● | ● | ● | A | ● | ● | ● | ● | ● | ● | ● | ● | T | ● |  |  |  |  |  |  |  |  |  |  |  |  |  |  |  |  |  |  |  |
|  | AB518500 | ● | ● | ● | ● | ● | ● | ● | ● | ● | T | ● | ● | ● | ● | ● | ● | C | ● | ● | ● | ● | C | ● | ● | ● | ● | ● | ● | ● | ● | A | ● | ● | ● | ● | ● | ● | ● | ● | T | ● |  |  |  |  |  |  |  |  |  |  |  |  |  |  |  |  |  |  |  |
|  | AB518501 | ● | ● | ● | ● | ● | ● | ● | ● | ● | T | ● | ● | ● | ● | ● | ● | C | ● | ● | ● | ● | T | ● | ● | ● | ● | ● | ● | ● | ● | A | ● | ● | ● | ● | ● | ● | ● | ● | T | ● |  |  |  |  |  |  |  |  |  |  |  |  |  |  |  |  |  |  |  |
|  | AB518502 | ● | ● | ● | ● | ● | ● | ● | ● | ● | T | ● | ● | ● | ● | ● | ● | C | ● | ● | ● | ● | T | ● | ● | ● | ● | ● | ● | ● | ● | A | ● | ● | ● | ● | ● | ● | ● | ● | T | ● |  |  |  |  |  |  |  |  |  |  |  |  |  |  |  |  |  |  |  |
|  | AB518503 | ● | ● | ● | ● | ● | ● | ● | ● | ● | T | ● | ● | ● | ● | ● | ● | C | ● | ● | ● | ● | T | ● | ● | ● | ● | ● | ● | ● | ● | A | ● | ● | ● | ● | ● | ● | ● | ● | T | ● |  |  |  |  |  |  |  |  |  |  |  |  |  |  |  |  |  |  |  |
|  | AB518504 | ● | ● | ● | ● | ● | ● | ● | ● | ● | T | ● | ● | ● | ● | ● | ● | C | ● | ● | ● | ● | T | ● | ● | ● | ● | ● | ● | ● | ● | A | ● | ● | ● | ● | ● | ● | ● | ● | T | ● |  |  |  |  |  |  |  |  |  |  |  |  |  |  |  |  |  |  |  |
|  | AB518505 | ● | ● | ● | ● | ● | ● | ● | ● | ● | T | ● | ● | ● | ● | ● | ● | C | ● | ● | ● | ● | T | ● | ● | ● | ● | ● | ● | ● | ● | A | ● | ● | ● | ● | ● | ● | ● | ● | T | ● |  |  |  |  |  |  |  |  |  |  |  |  |  |  |  |  |  |  |  |
|  | AB518506 | ● | ● | ● | ● | ● | ● | ● | ● | ● | T | ● | ● | ● | ● | ● | ● | C | ● | ● | ● | ● | T | ● | ● | ● | ● | ● | ● | ● | ● | A | ● | ● | ● | ● | ● | ● | ● | ● | T | ● |  |  |  |  |  |  |  |  |  |  |  |  |  |  |  |  |  |  |  |
|  | AB518507 | ● | ● | ● | ● | ● | ● | ● | ● | ● | T | ● | ● | ● | ● | ● | ● | C | ● | ● | ● | ● | T | ● | ● | ● | ● | ● | ● | ● | ● | A | ● | ● | ● | ● | ● | ● | ● | ● | T | ● |  |  |  |  |  |  |  |  |  |  |  |  |  |  |  |  |  |  |  |
|  | AB518508 | ● | ● | ● | ● | ● | ● | ● | ● | ● | T | ● | ● | ● | ● | ● | ● | C | ● | ● | ● | ● | T | ● | ● | ● | ● | ● | ● | ● | ● | A | ● | ● | ● | ● | ● | ● | ● | ● | T | ● |  |  |  |  |  |  |  |  |  |  |  |  |  |  |  |  |  |  |  |
|  | AB518509 | ● | ● | ● | ● | ● | ● | ● | ● | ● | T | ● | ● | ● | ● | ● | ● | C | ● | ● | ● | ● | T | ● | ● | ● | ● | ● | ● | ● | ● | A | ● | ● | ● | ● | ● | ● | ● | ● | T | ● |  |  |  |  |  |  |  |  |  |  |  |  |  |  |  |  |  |  |  |
|  | AB518510 | ● | ● | ● | ● | ● | ● | ● | ● | ● | T | ● | ● | ● | ● | ● | ● | C | ● | ● | ● | ● | T | ● | ● | ● | ● | ● | ● | ● | ● | A | ● | ● | ● | ● | ● | ● | ● | ● | T | ● |  |  |  |  |  |  |  |  |  |  |  |  |  |  |  |  |  |  |  |
|  | AB518511 | ● | ● | ● | ● | ● | ● | ● | ● | ● | T | ● | ● | ● | ● | ● | ● | C | ● | ● | ● | ● | T | ● | ● | ● | ● | ● | ● | ● | ● | A | ● | ● | ● | ● | ● | ● | ● | ● | T | ● |  |  |  |  |  |  |  |  |  |  |  |  |  |  |  |  |  |  |  |
|  | AB518512 | ● | ● | ● | ● | ● | ● | ● | ● | ● | T | ● | ● | ● | ● | ● | ● | C | ● | ● | ● | ● | T | ● | ● | ● | ● | ● | ● | ● | ● | A | ● | ● | ● | ● | ● | ● | ● | ● | T | ● |  |  |  |  |  |  |  |  |  |  |  |  |  |  |  |  |  |  |  |
|  | AB518513 | ● | ● | ● | ● | ● | ● | ● | ● | ● | T | ● | ● | ● | ● | ● | ● | C | ● | ● | ● | ● | T | ● | ● | ● | ● | ● | ● | ● | ● | A | ● | ● | ● | ● | ● | ● | ● | ● | T | ● |  |  |  |  |  |  |  |  |  |  |  |  |  |  |  |  |  |  |  |
|  | AB518514 | ● | ● | ● | ● | ● | ● | ● | ● | ● | T | ● | ● | ● | ● | ● | ● | C | ● | ● | ● | ● | T | ● | ● | ● | ● | ● | ● | ● | ● | A | ● | ● | ● | ● | ● | ● | ● | ● | T | ● |  |  |  |  |  |  |  |  |  |  |  |  |  |  |  |  |  |  |  |
|  | AB518515 | ● | ● | ● | ● | ● | ● | ● | ● | ● | T | ● | ● | ● | ● | ● | ● | C | ● | ● | ● | ● | T | ● | ● | ● | ● | ● | ● | ● | ● | A | ● | ● | ● | ● | ● | ● | ● | ● | T | ● |  |  |  |  |  |  |  |  |  |  |  |  |  |  |  |  |  |  |  |
|  | AB518516 | ● | ● | ● | ● | ● | ● | ● | ● | ● | T | ● | ● | ● | ● | ● | ● | C | ● | ● | ● | ● | T | ● | ● | ● | ● | ● | ● | ● | ● | A | ● | ● | ● | ● | ● | ● | ● | ● | T | ● |  |  |  |  |  |  |  |  |  |  |  |  |  |  |  |  |  |  |  |
|  | AB518517 | ● | ● | ● | ● | ● | ● | ● | ● | ● | T | ● | ● | ● | ● | ● | ● | C | ● | ● | ● | ● | T | ● | ● | ● | ● | ● | ● | ● | ● | A | ● | ● | ● | ● | ● | ● | ● | ● | T | ● |  |  |  |  |  |  |  |  |  |  |  |  |  |  |  |  |  |  |  |
|  | AB518518 | ● | ● | ● | ● | ● | ● | ● | ● | ● | T | ● | ● | ● | ● | ● | ● | C | ● | ● | ● | ● | T | ● | ● | ● | ● | ● | ● | ● | ● | A | ● | ● | ● | ● | ● | ● | ● | ● | T | ● |  |  |  |  |  |  |  |  |  |  |  |  |  |  |  |  |  |  |  |
|  | AB518519 | ● | ● | ● | ● | ● | ● | ● | ● | ● | T | ● | ● | ● | ● | ● | ● | C | ● | ● | ● | ● | T | ● | ● | ● | ● | ● | ● | ● | ● | A | ● | ● | ● | ● | ● | ● | ● | ● | T | ● |  |  |  |  |  |  |  |  |  |  |  |  |  |  |  |  |  |  |  |
|  | AB518520 | ● | ● | ● | ● | ● | ● | ● | ● | ● | T | ● | ● | ● | ● | ● | ● | C | ● | ● | ● | ● | T | ● | ● | ● | ● | ● | ● | ● | ● | A | ● | ● | ● | ● | ● | ● | ● | ● | T | ● |  |  |  |  |  |  |  |  |  |  |  |  |  |  |  |  |  |  |  |
|  | AB518521 | ● | ● | ● | ● | ● | ● | ● | ● | ● | T | ● | ● | ● | ● | ● | ● | C | ● | ● | ● | ● | T | ● | ● | ● | ● | ● | ● | ● | ● | A | ● | ● | ● | ● | ● | ● | ● | ● | T | ● |  |  |  |  |  |  |  |  |  |  |  |  |  |  |  |  |  |  |  |
|  | AB518522 | ● | ● | ● | ● | ● | ● | ● | ● | ● | T | ● | ● | ● | ● | ● | ● | ● | ● | ● | ● | ● | T | ● | ● | ● | ● | ● | ● | ● | ● | A | ● | ● | ● | ● | ● | ● | ● | ● | T | ● |  |  |  |  |  |  |  |  |  |  |  |  |  |  |  |  |  |  |  |
|  | AB518523 | ● | ● | ● | ● | ● | ● | ● | ● | ● | T | ● | ● | ● | ● | ● | ● | C | ● | ● | ● | ● | T | ● | ● | ● | ● | ● | ● | ● | ● | A | ● | ● | ● | ● | ● | ● | ● | ● | T | ● |  |  |  |  |  |  |  |  |  |  |  |  |  |  |  |  |  |  |  |
|  | AB518524 | ● | ● | ● | ● | ● | ● | ● | ● | ● | T | ● | ● | ● | ● | ● | ● | C | ● | ● | ● | ● | T | ● | ● | ● | ● | ● | ● | ● | ● | A | ● | ● | ● | ● | ● | ● | ● | ● | T | ● |  |  |  |  |  |  |  |  |  |  |  |  |  |  |  |  |  |  |  |
|  | AB518525 | ● | ● | ● | ● | ● | ● | ● | ● | ● | T | ● | ● | ● | ● | ● | ● | C | ● | ● | ● | ● | T | ● | ● | ● | ● | ● | ● | ● | ● | A | ● | ● | ● | ● | ● | ● | ● | ● | T | ● |  |  |  |  |  |  |  |  |  |  |  |  |  |  |  |  |  |  |  |
|  | AB518526 | ● | ● | ● | ● | ● | ● | ● | ● | ● | T | ● | ● | ● | ● | ● | ● | C | ● | ● | ● | ● | T | ● | ● | ● | ● | ● | ● | ● | ● | A | ● | ● | ● | ● | ● | ● | ● | ● | T | ● |  |  |  |  |  |  |  |  |  |  |  |  |  |  |  |  |  |  |  |
|  | AB518527 | ● | ● | ● | ● | ● | ● | ● | ● | ● | T | ● | ● | ● | ● | ● | ● | C | ● | ● | ● | ● | T | ● | ● | ● | ● | ● | ● | ● | ● | A | ● | ● | ● | ● | ● | ● | ● | ● | T | ● |  |  |  |  |  |  |  |  |  |  |  |  |  |  |  |  |  |  |  |
|  | AB518528 | ● | ● | ● | ● | ● | ● | ● | ● | ● | T | ● | ● | ● | ● | ● | ● | C | ● | ● | ● | ● | T | ● | ● | ● | ● | ● | ● | ● | ● | A | ● | ● | ● | ● | ● | ● | ● | ● | T | ● |  |  |  |  |  |  |  |  |  |  |  |  |  |  |  |  |  |  |  |
|  | AB518529 | ● | ● | ● | ● | ● | ● | ● | ● | ● | T | ● | ● | ● | ● | ● | ● | C | ● | ● | ● | ● | T | ● | ● | ● | ● | ● | ● | ● | ● | A | ● | ● | ● | ● | ● | ● | ● | ● | T | ● |  |  |  |  |  |  |  |  |  |  |  |  |  |  |  |  |  |  |  |
|  | AB518530 | ● | ● | ● | ● | ● | ● | ● | ● | ● | T | ● | ● | ● | ● | ● | ● | C | ● | ● | ● | ● | T | ● | ● | ● | ● | ● | ● | ● | ● | A | ● | ● | ● | ● | ● | ● | ● | ● | T | ● |  |  |  |  |  |  |  |  |  |  |  |  |  |  |  |  |  |  |  |
|  | AB518531 | ● | ● | ● | ● | ● | ● | ● | ● | ● | T | ● | ● | ● | ● | ● | ● | C | ● | ● | ● | ● | T | ● | ● | ● | ● | ● | ● | ● | ● | A | ● | ● | ● | ● | ● | ● | ● | ● | T | ● |  |  |  |  |  |  |  |  |  |  |  |  |  |  |  |  |  |  |  |
|  | AB518532 | ● | ● | ● | ● | ● | ● | ● | ● | ● | T | ● | ● | ● | ● | ● | ● | C | ● | ● | ● | ● | T | ● | ● | ● | ● | ● | ● | ● | ● | A | ● | ● | ● | ● | ● | ● | ● | ● | T | ● |  |  |  |  |  |  |  |  |  |  |  |  |  |  |  |  |  |  |  |
|  | AB518533 | ● | ● | ● | ● | ● | ● | ● | ● | ● | T | ● | ● | ● | ● | ● | ● | C | ● | ● | ● | ● | T | ● | ● | ● | ● | ● | ● | ● | ● | A | ● | ● | ● | ● | ● | ● | ● | ● | T | ● |  |  |  |  |  |  |  |  |  |  |  |  |  |  |  |  |  |  |  |
|  | DQ897944 | ● | ● | ● | ● | ● | ● | ● | ● | ● | T | ● | ● | ● | ● | ● | ● | C | ● | ● | ● | ● | T | ● | ● | ● | ● | ● | ● | ● | ● | A | ● | ● | ● | ● | ● | ● | ● | ● | T | ● |  |  |  |  |  |  |  |  |  |  |  |  |  |  |  |  |  |  |  |
|  | DQ897945 | ● | ● | ● | ● | ● | ● | ● | ● | ● | T | ● | ● | ● | ● | ● | ● | C | ● | ● | ● | ● | T | ● | ● | ● | ● | ● | ● | ● | ● | A | ● | ● | ● | ● | ● | ● | ● | ● | T | ● |  |  |  |  |  |  |  |  |  |  |  |  |  |  |  |  |  |  |  |
|  | DQ897946 | ● | ● | ● | ● | ● | ● | ● | ● | ● | T | ● | ● | ● | ● | ● | ● | C | ● | ● | ● | ● | T | ● | ● | ● | ● | ● | ● | ● | ● | A | ● | ● | ● | ● | ● | ● | ● | ● | T | ● |  |  |  |  |  |  |  |  |  |  |  |  |  |  |  |  |  |  |  |
| Ele | DQ897957 | ● | ● | ● | ● | ● | ● | ● | ● | ● | T | ● | ● | ● | ● | ● | ● | ● | ● | ● | ● | ● | T | ● | ● | ● | ● | ● | ● | ● | ● | A | ● | ● | ● | ● | ● | ● | ● | ● | T | ● |  |  |  |  |  |  |  |  |  |  |  |  |  |  |  |  |  |  |  |
|  | DQ897958 | ● | ● | ● | ● | ● | ● | ● | ● | ● | T | ● | ● | ● | ● | ● | ● | ● | ● | ● | ● | ● | T | ● | ● | ● | ● | ● | ● | ● | ● | A | ● | ● | ● | ● | ● | ● | ● | ● | T | ● |  |  |  |  |  |  |  |  |  |  |  |  |  |  |  |  |  |  |  |
| Gam | L20934 | ● | ● | ● | ● | ● | ● | ● | ● | ● | ● | ● | ● | ● | ● | ● | C | C | ● | T | ● | ● | T | ● | ● | T | ● | ● | ● | ● | ● | A | ● | ● | T | ● | ● | T | ● | ● | T | ● |  |  |  |  |  |  |  |  |  |  |  |  |  |  |  |  |  |  |  |
| Int | KM032605 | ● | ● | ● | ● | ● | ● | ● | ● | ● | T | ● | ● | ● | ● | ● | ● | ● | ● | ● | ● | ● | T | ● | ● | ● | ● | ● | ● | ● | ● | A | ● | ● | T | ● | ● | ● | ● | ● | T | ● |  |  |  |  |  |  |  |  |  |  |  |  |  |  |  |  |  |  |  |
|  | KM032606 | ● | ● | ● | ● | ● | ● | ● | ● | ● | T | ● | ● | ● | ● | ● | ● | ● | ● | ● | ● | ● | T | ● | ● | ● | ● | ● | ● | ● | ● | A | ● | ● | T | ● | ● | ● | ● | ● | T | ● |  |  |  |  |  |  |  |  |  |  |  |  |  |  |  |  |  |  |  |
|  | KM032607 | ● | ● | ● | ● | ● | ● | ● | ● | ● | T | ● | ● | ● | ● | ● | ● | ● | ● | ● | ● | ● | T | ● | ● | ● | ● | ● | ● | ● | ● | A | ● | ● | T | ● | ● | ● | ● | ● | T | ● |  |  |  |  |  |  |  |  |  |  |  |  |  |  |  |  |  |  |  |
|  | KM032608 | ● | ● | ● | ● | ● | ● | ● | ● | ● | T | ● | ● | ● | ● | ● | ● | ● | ● | ● | ● | ● | T | ● | ● | ● | ● | ● | ● | ● | ● | A | ● | ● | T | ● | ● | ● | ● | ● | T | ● |  |  |  |  |  |  |  |  |  |  |  |  |  |  |  |  |  |  |  |
|  | KM032609 | ● | ● | ● | ● | ● | ● | ● | ● | ● | T | ● | ● | ● | ● | ● | ● | ● | ● | ● | ● | ● | T | ● | ● | ● | ● | ● | ● | ● | ● | A | ● | ● | T | ● | ● | ● | ● | ● | T | ● |  |  |  |  |  |  |  |  |  |  |  |  |  |  |  |  |  |  |  |
|  | KM032610 | ● | ● | ● | ● | ● | ● | ● | ● | ● | T | ● | ● | ● | ● | ● | ● | ● | ● | ● | ● | ● | T | ● | ● | ● | ● | ● | ● | ● | ● | A | ● | ● | T | ● | ● | ● | ● | ● | T | ● |  |  |  |  |  |  |  |  |  |  |  |  |  |  |  |  |  |  |  |
|  | KM032611 | ● | ● | ● | ● | ● | ● | ● | ● | ● | T | ● | ● | ● | ● | ● | ● | ● | ● | ● | ● | ● | T | ● | ● | ● | ● | ● | ● | ● | ● | A | ● | ● | T | ● | ● | ● | ● | ● | T | ● |  |  |  |  |  |  |  |  |  |  |  |  |  |  |  |  |  |  |  |
|  | KM032612 | ● | ● | ● | ● | ● | ● | ● | ● | ● | T | ● | ● | ● | ● | ● | ● | ● | ● | ● | ● | ● | T | ● | ● | ● | ● | ● | ● | ● | ● | A | ● | ● | T | ● | ● | ● | ● | ● | T | ● |  |  |  |  |  |  |  |  |  |  |  |  |  |  |  |  |  |  |  |
| Lat | DQ897936 | ● | ● | ● | ● | ● | ● | ● | ● | ● | T | ● | ● | C | ● | ● | ● | ● | ● | ● | ● | ● | T | ● | ● | ● | ● | ● | ● | ● | ● | A | ● | ● | T | ● | ● | T | ● | ● | T | ● |  |  |  |  |  |  |  |  |  |  |  |  |  |  |  |  |  |  |  |
|  | DQ897937 | ● | ● | ● | ● | ● | ● | ● | ● | ● | T | ● | ● | C | ● | ● | ● | ● | ● | ● | ● | ● | T | ● | ● | ● | ● | ● | ● | ● | ● | A | ● | ● | ● | ● | ● | T | ● | ● | ● | ● |  |  |  |  |  |  |  |  |  |  |  |  |  |  |  |  |  |  |  |
| Leu | DQ897939 | ● | ● | ● | ● | ● | ● | ● | ● | ● | ● | ● | ● | ● | ● | ● | ● | C | ● | ● | ● | ● | T | ● | ● | ● | ● | ● | ● | ● | ● | A | ● | ● | T | ● | ● | T | ● | ● | ● | ● |  |  |  |  |  |  |  |  |  |  |  |  |  |  |  |  |  |  |  |
| Mac | DQ897969 | ● | ● | ● | ● | ● | ● | ● | ● | ● | ● | ● | ● | ● | ● | ● | ● | C | ● | T | ● | ● | C | ● | ● | ● | ● | ● | ● | ● | ● | A | ● | ● | ● | ● | ● | ● | ● | ● | T | ● |  |  |  |  |  |  |  |  |  |  |  |  |  |  |  |  |  |  |  |
|  | DQ897970 | ● | ● | ● | ● | ● | ● | ● | ● | ● | ● | ● | ● | ● | ● | ● | ● | C | ● | T | ● | ● | C | ● | ● | ● | ● | ● | ● | ● | ● | A | ● | ● | ● | ● | ● | ● | ● | ● | T | ● |  |  |  |  |  |  |  |  |  |  |  |  |  |  |  |  |  |  |  |
|  | DQ897971 | ● | ● | ● | ● | ● | ● | ● | ● | ● | ● | ● | ● | ● | ● | ● | ● | C | ● | T | ● | ● | C | ● | ● | ● | ● | ● | ● | ● | ● | A | ● | ● | ● | ● | ● | ● | ● | ● | T | ● |  |  |  |  |  |  |  |  |  |  |  |  |  |  |  |  |  |  |  |
|  | DQ897972 | ● | ● | ● | ● | ● | ● | ● | ● | ● | ● | ● | ● | ● | ● | ● | ● | C | ● | T | ● | ● | C | ● | ● | ● | ● | ● | ● | ● | ● | A | ● | ● | ● | ● | ● | ● | ● | ● | T | ● |  |  |  |  |  |  |  |  |  |  |  |  |  |  |  |  |  |  |  |
| Mir | DQ897965 | ● | ● | ● | G | ● | ● | ● | ● | ● | T | ● | ● | ● | C | ● | ● | ● | ● | ● | ● | ● | T | ● | ● | ● | ● | ● | ● | ● | ● | A | ● | ● | T | ● | ● | T | ● | ● | T | ● |  |  |  |  |  |  |  |  |  |  |  |  |  |  |  |  |  |  |  |
|  | DQ897966 | ● | ● | ● | G | ● | ● | ● | ● | ● | T | ● | ● | C | C | ● | ● | ● | ● | ● | ● | ● | C | ● | ● | ● | ● | ● | ● | ● | ● | A | ● | ● | T | ● | ● | T | ● | ● | T | ● |  |  |  |  |  |  |  |  |  |  |  |  |  |  |  |  |  |  |  |
| Nem | DQ897959 | ● | ● | ● | ● | ● | ● | ● | ● | ● | T | ● | ● | ● | ● | ● | ● | ● | ● | ● | ● | ● | T | ● | ● | ● | ● | ● | ● | ● | ● | A | ● | ● | T | ● | ● | ● | ● | ● | T | ● |  |  |  |  |  |  |  |  |  |  |  |  |  |  |  |  |  |  |  |
|  | DQ897960 | ● | ● | ● | ● | ● | ● | ● | ● | ● | T | ● | ● | ● | ● | ● | ● | ● | ● | G | ● | ● | T | ● | ● | ● | ● | ● | ● | ● | ● | A | ● | ● | T | ● | ● | ● | ● | ● | T | ● |  |  |  |  |  |  |  |  |  |  |  |  |  |  |  |  |  |  |  |
|  | DQ897961 | ● | ● | ● | ● | ● | ● | ● | ● | ● | ● | ● | ● | C | ● | ● | ● | ● | ● | ● | ● | ● | T | ● | ● | ● | ● | ● | ● | ● | ● | A | ● | ● | ● | ● | ● | ● | ● | ● | T | ● |  |  |  |  |  |  |  |  |  |  |  |  |  |  |  |  |  |  |  |
| Sca | DQ897949 | ● | ● | ● | ● | ● | ● | ● | ● | ● | ● | ● | ● | C | ● | ● | ● | ● | ● | ● | ● | ● | T | ● | ● | ● | ● | ● | ● | ● | ● | A | ● | ● | ● | ● | ● | ● | ● | ● | T | ● |  |  |  |  |  |  |  |  |  |  |  |  |  |  |  |  |  |  |  |
| Tak cf | AB518534 | ● | ● | ● | ● | ● | ● | ● | ● | ● | ● | ● | ● | C | ● | ● | ● | ● | ● | ● | ● | ● | C | ● | ● | ● | ● | ● | ● | ● | ● | A | ● | ● | ● | ● | ● | ● | ● | ● | ● | ● |  |  |  |  |  |  |  |  |  |  |  |  |  |  |  |  |  |  |  |
|  | AB518535 | ● | ● | ● | ● | ● | ● | ● | ● | ● | ● | ● | ● | C | ● | ● | ● | ● | ● | ● | ● | ● | C | ● | ● | ● | ● | ● | ● | ● | ● | A | ● | ● | ● | ● | ● | ● | ● | ● | ● | ● |  |  |  |  |  |  |  |  |  |  |  |  |  |  |  |  |  |  |  |
|  | AB518536 | ● | ● | ● | ● | ● | ● | ● | ● | ● | T | ● | ● | C | ● | ● | ● | ● | ● | ● | ● | ● | T | ● | ● | ● | ● | ● | ● | ● | ● | A | ● | ● | ● | ● | ● | ● | ● | ● | ● | ● |  |  |  |  |  |  |  |  |  |  |  |  |  |  |  |  |  |  |  |
|  | AB518537 | ● | ● | ● | ● | ● | ● | ● | ● | ● | T | ● | ● | C | ● | ● | ● | ● | ● | ● | ● | ● | T | ● | ● | ● | ● | ● | ● | ● | ● | A | ● | ● | ● | ● | ● | ● | ● | ● | ● | ● |  |  |  |  |  |  |  |  |  |  |  |  |  |  |  |  |  |  |  |
|  | AB518538 | ● | ● | ● | ● | ● | ● | ● | ● | ● | T | ● | ● | C | ● | ● | ● | ● | ● | ● | ● | ● | T | ● | ● | ● | ● | ● | ● | ● | ● | A | ● | ● | ● | ● | ● | ● | ● | ● | ● | ● |  |  |  |  |  |  |  |  |  |  |  |  |  |  |  |  |  |  |  |
| Tak | DQ897962 | ● | ● | ● | ● | ● | ● | ● | ● | ● | T | ● | ● | C | ● | ● | ● | ● | ● | ● | ● | ● | T | ● | ● | ● | ● | ● | ● | ● | ● | A | ● | ● | ● | ● | ● | ● | ● | ● | T | ● |  |  |  |  |  |  |  |  |  |  |  |  |  |  |  |  |  |  |  |
|  | DQ897963 | ● | ● | ● | ● | ● | ● | ● | ● | ● | T | ● | ● | C | ● | ● | ● | ● | ● | ● | ● | ● | T | ● | ● | ● | ● | ● | ● | ● | ● | A | ● | ● | ● | ● | ● | ● | ● | ● | T | ● |  |  |  |  |  |  |  |  |  |  |  |  |  |  |  |  |  |  |  |
|  | DQ897964 | ● | ● | ● | ● | ● | ● | ● | ● | ● | T | ● | ● | C | ● | ● | ● | ● | ● | ● | ● | ● | T | ● | ● | ● | ● | ● | ● | ● | ● | A | ● | ● | ● | ● | ● | ● | ● | ● | T | ● |  |  |  |  |  |  |  |  |  |  |  |  |  |  |  |  |  |  |  |

Bal = *An. balabacensi*s, Bai = *An. baimaii*, Cra = *An. cracens*, Dir = *An. dirus*, Ele = *An. elegans*, Gam = *An. gambiae*, Int = *An. introlatus*, Lat = *An. latens*, Leu = *An. leucosphyrus*, Mac = *An. macarthuri*, Mir = *An. mirans*, Nem = *An. nemophilous*, Sca = *An. scanloni*, Tak = *An. takasagoensis*, cf = confer

**● = nucleotide identical to sequence DQ897940
